# Bony labyrinth taxonomic identification and long-term evolution of livestock in the Southwest and Central Asia

**DOI:** 10.1098/rsos.261493

**Published:** 2026-10-07

**Authors:** Agathe Guignet, Hossein Davoudi, Adeline Hemelsdaël, Delphine Decruyenaere, Adeline Vautrin, Renaud Lebrun, Romain Peronnet, Homa Fathi, Hitomi Hongo, Sarieh Amiri, Sanaz Beizaee Doost, Roya Khazaeli, Karyne Debue, Asma Ayoob Khiljee, Safoora Kamjan, Azadeh F. Mohaseb, Yasmine Martin, Rémi Berthon, Emmanuelle Vila, Jacopo Bruno, Abolfazl Aali, Akbar Abedi, Ahmad Aliyari, Mohammad H. Azizi Kharanaghi, Fereidoun Biglari, Raffaele Biscione, Alisher Begmatov, Amridin Berdimuradov, Gennadiy Bogomolov, Nicole Boenke, Julien Cuny, Aurore Didier, Iulon Gagoshidze, Irina Gambashidze, Bruno Genito, Teramura Hirofumi, Mohsen Javeri, Hossein A. Kavosh, Morteza Khanipour, Haeedeh Laleh, Zahra Lorzadeh, Zurab Makharadze, Catherine Marro, Jamal Mirzaakhmedov, Niloufar Moghimi, Tomomi Murakami, Reza Naseri, Jebrael Nokandeh, Marina Puturidze, Babak Rafiei-Alavi, Sobir Raimkulov, Roghayeh Rahimi Sorkhani, Rocco Rante, Elena Rova, Muminxon Saidov, Alisher Sandiboev, Eberhard Sauer, Ali Sajadi, Ali Shojaee-Esfahani, Denis Sorokin, Thomas Stöllner, Kamal Taheri, Akira Tsuneki, Ali A. Vahdati, Dmitryi Voyakin, Liangren Zhang, Marjan Mashkour, Allowen Evin

**Affiliations:** ^1^ Université de Montpellier, Institut des sciences de l'évolution de Montpellier, Montpellier, Occitanie, France; ^2^ Bioarchaeology Laboratory, Central Laboratory, University of Tehran, Tehran, Iran; ^3^ National Museum of Iran, Tehran, Iran; ^4^ UMR 7209—BioArch, Muséum National d'Histoire Naturelle, Paris, Île-de-France, France; ^5^ Silk Road International University of Tourism and Cultural Heritage, Samarkand, Uzbekistan; ^6^ Ruhr-Universität Bochum, Bochum, Germany; ^7^ Institut des sciences de l'évolution de Montpellier, Montpellier, Occitanie, France; ^8^ The Graduate University for Advanced Studies Research Center for Integrative Evolutionary Science, Hayama, Kanagawa Prefecture, Japan; ^9^ University of Georgia, Athens, GA, USA; ^10^ Mission Archéologique Française du Bassin de l'Indus, Pakistan; ^11^ Pir Mehr Ali Shah Arid Agriculture University, Punjab, Pakistan; ^12^ Centre for Isotope Research, University of Groningen, Groningen, The Netherlands; ^13^ UMR 5133 - Archéorient, Environnements et sociétés de l'Orient ancien, Université Lyon 2, Lyon Auvergne-Rhône-Alpes, France; ^14^ Institute for Iranian Studies, Austrian Academy of Sciences, Vienna, Austria; ^15^ MCTH, Zanjan Archaeological Museum, Zanjan, Iran; ^16^ Department of Archaeology and Archaeometry, Tabriz Islamic Art University, Tabriz, Iran; ^17^ Department of Archaeology, Faculty of Literature and Human Sciences, University of Tehran, Tehran, Iran; ^18^ Iranian Center for Archaeological Research, RICHT, Tehran, Iran; ^19^ Istituto di Scienze del Patrimonio Culturale, Consiglio Nazionale delle Ricerche, Rome, Italy; ^20^ Berlin-Brandenburg Academy of Sciences and Humanities, Berlin, Germany; ^21^ Archaeological Institute of Samarkand, Samarkand, Uzbekistan; ^22^ National Center of Archaeology of the Academy of Sciences of the Republic of Uzbekistan, Tashkent, Tashkent Province, Uzbekistan; ^23^ Département des Antiquités orientales, Musée du Louvre, Paris, France; ^24^ UMR 7041—ArScAn, Archéologie de l'Asie Centrale, Archeologies et Sciences de l'Antiquite, Nanterre, Île-de-France, France; ^25^ Tamaz Beradze Institute of Georgian Studies, The University of Georgia, Tbilisi, Georgia; ^26^ Georgian National Museum, Tbilisi, Georgia; ^27^ Università degli Studi di Napoli L'Orientale Dipartimento Asia Africa e Mediterraneo, Naples, Campania, Italy; ^28^ Minpaku, Suita, Osaka Prefecture, Japan; ^29^ Department of Archaeology, University of Kashan, Kashan, Iran; ^30^ Department of Archaeology, University of Zabol, Zabol, Iran; ^31^ UMR 8086—TEMPS, CNRS, Paris, France; ^32^ Research Institute for the Dynamics of Civilizations, Okayama University, Okayama, Japan; ^33^ Ivane Javakhishvili State University, Tbilisi, Georgia; ^34^ Aga Khan Cultural Service-Afghanistan, Kabul, Afghanistan; ^35^ Faculty of Art and Architecture, University of Mazandaran, Babolsar, Iran; ^36^ Ca' Foscari University, Venice, Italy; ^37^ Samarkand State University named after Alisher Navoi, Samarkand, Samarkand Province, Uzbekistan; ^38^ The University of Edinburgh School of History Classics and Archaeology, Edinburgh, UK; ^39^ Department of Archaeology, Faculty of Conservation and Restoration, Isfahan University of Art, Isfahan, Iran; ^40^ International Institute for Central Asian Studies, Samarkand, Uzbekistan; ^41^ Kermanshah Regional Water Authority, Kermanshah, Iran; ^42^ West Asian Archaeology, University of Tsukuba, Tsukuba, Japan; ^43^ General Directorate of North Khorasan Province, MCHT, Bojnord, Iran; ^44^ Central Asian Archaeological Landscapes (CAAL), Institute of Archaeology, University College London, London, UK; ^45^ School of History, Nanjing University, Nanjing, Jiangsu, People's Republic of China; ^46^ CNRS, IRD, Université de Montpellier, Institut des Sciences de l'Evolution de Montpellier, Montpellier Occitanie, France

**Keywords:** geometric morphometrics, inner ear, bioarchaeology, archaeozoology, diachrony, domestication, Iranian plateau, Caucasus, Indus valley, Holocene

## Abstract

Southwest and Central Asia were pivotal regions for the domestication and dispersal of domestic herbivores. Here we investigate long-term variation in bony labyrinth morphology of domestic bovids (sheep, goats and cattle) across the last 10 000 years. We focused on the osseous cavity of the inner ear, located within the petrous bone. We analysed semicircular canal (SC) size and shape using micro-computed tomography and geometric morphometrics (GMM) on 78 modern reference specimens and 234 archaeological petrous bones from 51 sites spanning the Iranian Plateau, Central Asia, the Caucasus and the Indus Valley. Bony labyrinth morphometrics yielded a strong taxonomic signal, enabling species-level identification of 228 out of 234 specimens and revealing greater diversity than anticipated. Comparative analyses further documented contrasting, nonlinear diachronic trajectories in SC size and shape among sheep, goats and cattle, reflecting species-specific responses to domestication and long-term herd management. Together, these results demonstrate that bony labyrinth GMM provides a robust, non-destructive framework for taxonomic identification and evolutionary inference and underscore how long-term, interdisciplinary approaches can capture the complex, nonlinear dynamics of animal domestication across the longue durée.

## 1. Introduction

Southwest and Central Asia constitute a vast region encompassing several geographical and cultural entities in a generally arid to semi-arid zone, yet characterised by high ecological diversity that has shaped subsistence strategies through time, notably through the herding of bovids. The Fertile Crescent is recognised as the primary centre of domestication for major livestock species—cattle (*Bos taurus*), pig (*Sus scrofa*), goat (*Capra hircus*) and sheep (*Ovis aries*)*—*around 9000–8000 cal. BCE [1]. Together with early domestic plants such as wheat, barley and pulses (e.g. [2]), these species formed the foundation of Neolithic agro-pastoral economies and played a central role in the technical, socio-economic and demographic changes in human societies [3]. This context offers an ideal setting to explore how livestock were impacted by contrasting environments, cultural practices, herd management strategies and selection pressures over millennia.

Archaeological and genetic studies converge in identifying the Fertile Crescent as a cradle of domestication (review in [4,5]). Despite this consensus, the subsequent evolutionary trajectories of the domestic species, particularly in the neighbouring regions of the Iranian Plateau, South Central Asia, the Caucasus and the Indus Valley, remain insufficiently understood.

Key questions concern how these populations diversified morphologically and adapted to distinct environments and management systems over millennia. Ancient genomic analyses indicate that sheep were domesticated from the Asiatic mouflon and that early Iranian Plateau populations formed a genetically distinct ‘Neolithic East’ cluster, separate from western Neolithic flocks, suggesting partially independent evolutionary trajectories [6]. For goats, genomic evidence from Zagros Mountain sites demonstrates that managed herds, genetically distinct from wild bezoar goats, were established by 8200 cal. BCE, positioning western Iran as a primary centre of goat domestication [7]. For cattle, ancient genomic analysis of Near Eastern cattle reveals marked regional variation prior to the mid‑third millennium BCE, followed by a major Bronze Age demographic shift driven by the introgression of zebu [8].

In the study zone, the Iranian Plateau is a key region for studying early livestock evolution [9] (supplementary material). Archaeological evidence indicates that Neolithisation began in the Central Zagros in the ninth millennium BCE and spread across the Iranian Plateau and neighbouring regions by the mid-eighth millennium BCE [10]. The Zagros and Alborz mountains preserved wild progenitors and provided favourable contexts for the early development of agro-pastoral practices [11]. Analysis of two Early Neolithic sites (Abdul Hosein and Ganj Dareh in the Zagros) revealed the presence of both domestic- and wild-affinity goats, suggesting simultaneous management and wild-resource exploitation and indicating circulation across the Zagros regions [7]. As for sheep, despite their broad geographic distribution, it is interesting to note that the Neolithic sheep from Iran, Azerbaijan and Georgia genetically clustered closely, reflecting connectivity among pastoral communities during the sixth millennium BCE [6]. Such affinities may equally mirror the broader demographic landscape of the region, where early South Caucasian populations already displayed a mixed ancestry combining local Caucasus hunter‑gatherer‑related components with inputs from Iranian and Anatolian Neolithic groups, as demonstrated by recent palaeogenomic studies [12,13]. Over the following millennia, this region became a major hub of livestock diversification and management, shaped by cultural and economic networks linking Mesopotamia, the Iranian Plateau, the Caucasus, Central Asia and the Indus Valley (supplementary material). By at least 6000 BCE, early dispersals of domesticated sheep had reached Obishir V (Fergana Valley, modern Kyrgyzstan), a key corridor on routes leading toward China [14] (supplementary material). Capturing these dynamics requires robust analytical frameworks capable of tracing management practices and species differentiation through time. In parallel, decades of archaeozoological research on the exploitation of sheep, goats and cattle during the Holocene in the study area have built a solid foundation for understanding their use in prehistoric and historic communities over the last 10 000 years (e.g. [10,15–24]).

## 2. State of the art

Over recent decades of archaeozoological research, considerable effort has been devoted to distinguishing sheep from goats, as these species lie at the heart of early domestication processes in the region and, together with cattle, form the core of early agro-pastoral economies. Because of their close morphological similarity, this distinction has long represented a methodological challenge, which has increasingly been addressed through biomolecular and quantitative approaches, including paleo proteins [25], ancient DNA [6,7] and geometric morphometrics (GMM) (e.g. [26,27]). These methods have become essential tools for species identification and the reconstruction of biocultural histories, substantially improving the differentiation of morphologically similar taxa such as sheep and goats [28,29]. Accordingly, GMM, which quantifies variation in shape and size in skeletal elements such as bones and teeth, has been widely applied in bioarchaeology to distinguish species, establish the wild or domestic status and characterize population differences structured by time or geography (e.g. [30–32]).

Among the anatomical structures investigated using this approach, the bony labyrinth has attracted increasing attention. The bony labyrinth is an osseous structure housed within the petrous bone of the temporal bone that forms part of the inner ear. The inner ear comprises both the bony labyrinth and the membranous labyrinth, the latter consisting of interconnected ducts and sacs enclosed within the osseous cavity [33]. Because the membranous labyrinth rarely preserves and is generally inaccessible in osteological material, the bony labyrinth is commonly used as a proxy for investigating inner ear morphology and function (e.g. [34,35]). Within this osseous structure, two main functional regions are recognised: the inferior division, the cochlea, involved in auditory perception and the detection of linear acceleration [36], and the superior division, the vestibular system, formed partly by the semicircular canals (SCs), involved in the perception of head rotations and the maintenance of equilibrium [33]. Beyond its functional relevance, the petrous bone has been extensively targeted over the past decade for its exceptional preservation of biomolecules [37,38]. Systematic digital archiving through three-dimensional surface and microtomographic (µCT) scanning prior to any destructive analyses further enhances its value for integrative research [39–42]. Morphometric analyses of the bony labyrinth have proven highly effective for distinguishing closely related taxa [43,44], differentiating wild and domestic populations [45], identifying geographic origins [46] and exploring population history [47]. Among inner ear structures, the SCs have consistently been shown to provide particularly strong taxonomic discrimination. In equids, discrimination based on SCs morphology substantially outperformed cochlear morphology, whereas combining both structures only yielded a moderate increase in predictive accuracy [44]. These results suggest that SCs provide the most informative component of the bony labyrinth for taxonomic discrimination, while requiring considerably less processing effort than analyses incorporating the cochlea. The cochlea also poses inherent challenges for landmark-based GMM because differences in the degree of coiling among taxa complicate the establishment of homologous landmark positions and may influence generalised Procrustes superimposition independently of local shape variation. In sheep and goats, morphometrics of the SCs perfectly discriminate between the two species [48], allowing the identification of archaeological specimens whose taxonomic attribution was validated by ancient DNA data [49]. However, this powerful approach remains under-exploited in the investigation of livestock evolution over time. Despite this potential, bony labyrinth morphometrics remain underused in studies of long-term livestock evolution, owing to the relative scarcity of petrous bones in archaeological assemblages, limited access to high-resolution µCT facilities and the lack of extensive reference datasets.

The present study investigates bony labyrinth morphology in archaeological and modern specimens of sheep, goats and cattle spanning the last 11 000 years, with a primary focus on Southwest and Central Asia. The analysed material derives predominantly from the Iranian Plateau and the Zagros, encompassing the full extent of present-day Iran from the Late Palaeolithic to the Medieval period. Additional specimens from the South Caucasus, South Central Asia and the Indus Valley are less numerous, but together still represent 79 of the 234 analysed samples. Within this broad spatial and temporal framework, the following questions were addressed. (i) What is the taxonomic diversity represented in the studied material, and were taxa beyond the targeted domestic species present? (ii) How did bony labyrinth size and shape vary diachronically in goats, sheep and cattle? (iii) Do these three species follow distinct evolutionary trajectories?

## 3. Material and methods

### 3.1. Modern reference material

To establish a comparative framework for species identification, we assembled a modern reference dataset of 78 three-dimensional bony labyrinth models representing 21 taxa present in the region (supplementary material, table S1). Nineteen taxa were represented by one to four individuals: cattle (*Bos taurus*), dog (*Canis lupus familiaris*), roe deer (*Capreolus capreolus*), red deer (*Cervus elaphus*), fallow deer (*Dama mesopotamica*), donkey (*Equus asinus*), horse (*Equus caballus*), cat (*Felis catus*), Arabian sand gazelle (*Gazella marica*), goitered gazelle (*Gazella subgutturosa*), human (*Homo sapiens*), hare (*Lepus europaeus*), rabbit (*Oryctolagus cuniculus*), Asiatic mouflon (*Ovis orientalis*), urial (*Ovis vignei*), Saiga antelope (*Saiga tatarica*), wild boar (*Sus scrofa*), pig (*Sus scrofa domesticus*) and fox (*Vulpes vulpes*). The sheep (*Ovis aries*) and the goat (*Capra hircus*) were represented by 26 and 15 specimens, respectively. Models were newly generated or sourced from published datasets (supplementary material, table S1).

### 3.2. Archaeological material

A total of 234 complete bony labyrinths were newly extracted from petrous bones (supplementary material, table S1) originating from 51 archaeological sites (supplementary material, table S2) across the Iranian Plateau (Iran, *n* = 155) and adjacent regions: Upper Mesopotamia (Iraq, *n* = 14), Central Asia (Uzbekistan, *n* = 24), the Indus Valley (Pakistan, *n* = 7), the Caucasus (Georgia, *n* = 32; Azerbaijan, *n* = 1), and Anatolia (Türkiye, *n* = 1) (figure 1). The assemblage spans the Early Neolithic to the Medieval period (supplementary material, table S3).

**Figure 1.**
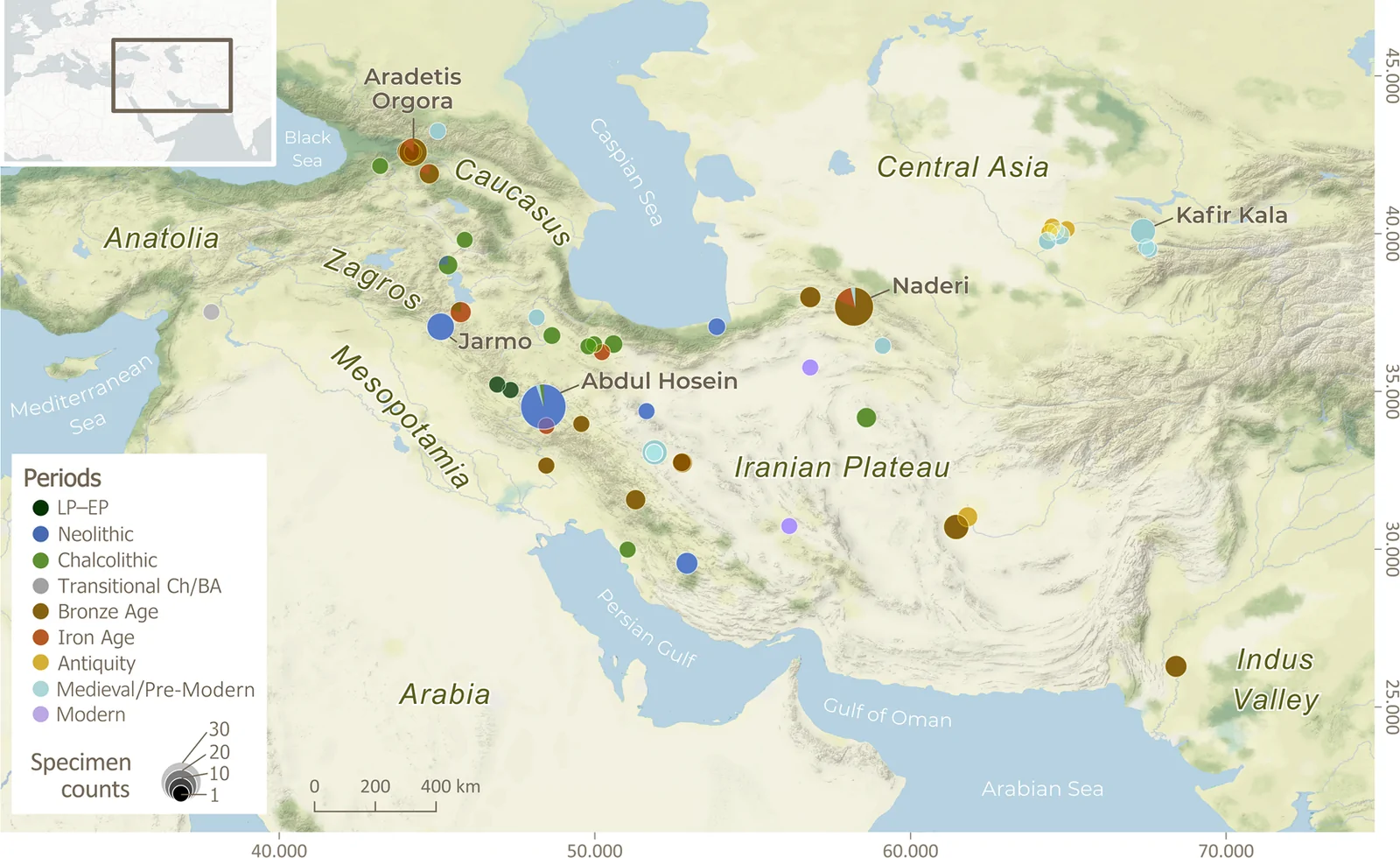
Geographical distribution of the 51 archaeological sites analysed. Dots indicate site locations and are coloured according to associated periods. Dot size is proportional to the number of specimens analysed per site, with the names of the larger assemblages mentioned in the study. The inset shows the broader geographical context of the study area. Period abbreviations: LP–EP, Late Palaeolithic–Epipalaeolithic; Ch, Chalcolithic; BA, Bronze Age. Detailed site information is provided in figure S1 and table S2 in the supplementary material.

Period attribution was based on stratigraphy and published radiocarbon or typological dating. Specimens were grouped into seven broad periods: Late Palaeolithic-Epipalaeolithic (20 000–10 000/9000 BCE [50]), Neolithic (approx. 9000–5000 BCE [51]), Chalcolithic (approx. 5000–3400 BCE [52]), Bronze Age (approx. 3400–1250 BCE [53]), Iron Age (approx. 1250–550 BCE [54]), Antiquity (approx. 550 BCE–650 CE [55,56]), and Medieval (approx. 650 to 15/16th century CE [57]) periods (supplementary material, table S3). For each specimen, the *terminus post quem* (TPQ) and *terminus ante quem* (TAQ) were recorded (supplementary material, table S1).

### 3.3. Preliminary taxonomic identification

Where preservation allowed, specimens were provisionally identified using diagnostic cranial traits or external petrous morphology [58,59]. Owing to substantial overlap between caprine taxa and domestic populations, many specimens remained indeterminate prior to morphometric analysis.

### 3.4. µCT acquisition and geometric morphometrics

Most of the archaeological petrous bones were scanned using an EasyTom 150 kV system (MRI platform, ISEM, Montpellier) at a voxel size of 53–68 µm, a voltage of 70–100 kV and an aluminium filter. Some of the modern and archaeological petrous bones were scanned using an EasyTom S 150 (AST-RX platform, MNHN, Paris) at a voxel size of 59 µm, a voltage of 150 kV and a copper filter. The bony labyrinth was segmented, and three-dimensional models were reconstructed using Avizo v2019.1, Amira v.6.7 (Thermo Fisher Scientific) and Biomedisa [60]. Landmarking was performed in MorphoDig v.1.6.8 64-bit [61] on right bony labyrinths; left specimens were mirrored prior to analysis.

The landmark protocol followed [46] and included six anatomical landmarks and four curves of sliding semi-landmarks (equidistant points), spread as follows: 16 for both the anterior and posterior SCs, 23 for the lateral SC, and five for the *common crus* (CC) (figure 2A; supplementary material, figure S2). All three-dimensional coordinates were subjected to a general Procrustes superimposition (using the R function Morpho::procSym() [62]), with semi-landmarks allowed to slide along curves.

**Figure 2.**
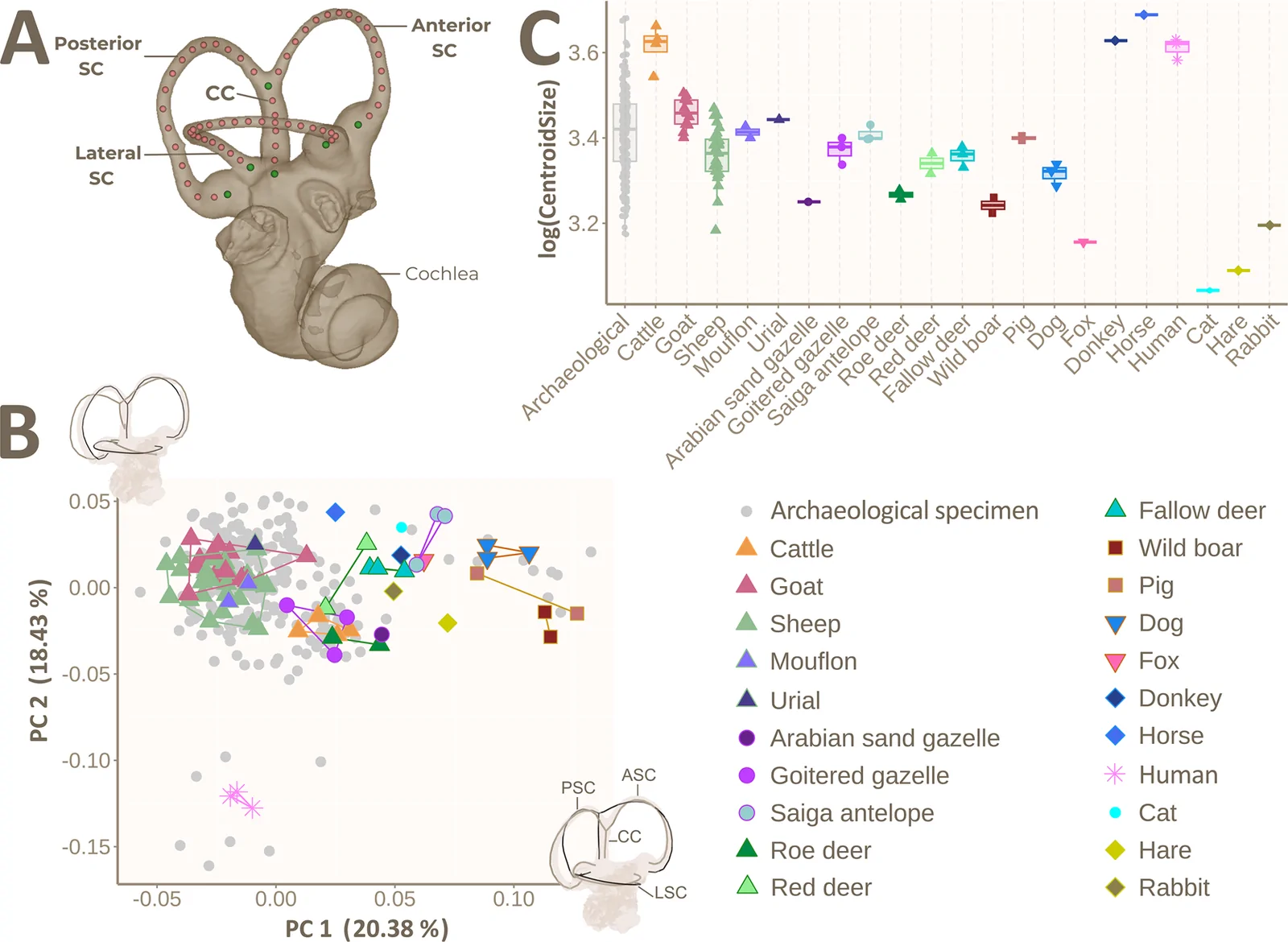
Bony labyrinth morphometric data and overall variation. (A) Landmarking protocol showing homologous landmarks (green) and sliding semi-landmarks (pink); analysed anatomical regions are indicated in bold. (B) Principal component analysis of semicircular canals (SCs) shape variation, with visualisation of reconstructed extreme shapes along PC1 and PC2. Shapes corresponding to minimum and maximum PC scores are shown in light brown and black, respectively. (C) Log-transformed centroid size variation of SCs. Abbreviations: ASC, anterior semicircular canal; PSC, posterior semicircular canal; LSC, lateral semicircular canal; CC, *common crus*.

### 3.5. Statistical analysis

All analyses were conducted in R [63] using RStudio v2023.12.1+402.

Measurement repeatability of landmark digitisation was assessed by repeated digitalisation of three modern sheep specimens (UM_BMC_2284, UM_BMC_2286 and UM_BMC_2287, already included in [48]) by two operators (AG and AH), with inter- and intra-operator error quantified following Claude [64].

Taxonomic identification relied on a combined supervised and unsupervised framework. Shape variation was first explored using principal component analysis (PCA) of Procrustes residuals. To visualise morphological variation, the specimen closest to the centroid of the PCA space (PC1–PC2) was identified for caprines and cattle. Extreme shapes along PCA and LDA axes were reconstructed from minimum and maximum scores (function Morpho::restoreShapes() [62]). Shape changes were visualised on a mean three-dimensional mesh aligned to the average landmark configuration, with deformation lines highlighting changes at opposing ends of each axis.

Discrimination between taxa (i.e., species or groups of species) was evaluated using resampling-based linear discriminant analyses (LDA) (100 iterations, *mevolCVP* function [65,66]), trained on modern reference taxa, with archaeological specimens treated as unknowns and identified using predictive LDA (pLDA) (100 iterations, *pldam* function [67]). Classification robustness was assessed using cross-validated prediction frequencies (CVP, with mean CVP and 90% interval provided). To limit data dimensionality, only the first six principal components (PCs) were used as input variables for LDA and pLDA. These PCs maximised group discrimination (with 88% of variance) while remaining a limited number.

To evaluate concordance between supervised and unsupervised approaches, a model-based clustering (R function mclust::Mclust() [68]) was performed on the same PC set.

For sheep, goats and cattle, centroid size (log-transformed) was used as a proxy for bony labyrinth size. Size and shape differences among species, periods and specimen status (archaeological versus modern) were visualised using boxplots tested using Wilcoxon rank‑sum tests for size (function ggubr::compare_means() [69]) and permutation-based linear models for shape (function geomorph::lm.rrpp() [70]). Diachronic trends were examined using both continuous time (TPQ–TAQ midpoints) and periods, incorporating time uncertainty through resampling procedures (function pataqu::spaghetti() [71]).

## 4. Results

### 4.1. Repeatability test

Intra-operator measurement error accounted for 3.4% and 4.5% of shape variance and 0.05% and 0.36% of size variance, while inter-operator error reached 9.7% for shape and 0.1% for size (supplementary material, figure S3 and table S4), supporting the reproducibility of landmark digitisation and validating the reuse of previously published coordinates [48].

### 4.2. Overall variation and sample identification

PCA—including all modern and archaeological specimens—revealed important variation in the shape of the SCs (figure 2B). Two main groups were apparent: (i) a small cluster comprising three modern humans and seven archaeological specimens; (ii) all remaining modern and archaeological non-hominid specimens (*n* = 303; figure 2B). Shape variation along PC1 involved the CC and posterior SC, which appeared wider at lower PC1 values. Along PC2, shape variation was more evenly distributed across the bony labyrinth. The orientation of the lateral SC largely contributed to the detection of this variation, which differed between human (minimal angle between LSC and CC) and non-human specimens (figure 2B).

Log-transformed centroid size distributions revealed pronounced interspecific differences, with archaeological specimens spanning the full range of modern taxa, except the small values of cat, hare and fox (figure 2C).

Using modern reference specimens, caprines (sheep, goats, Asiatic mouflon and urial) were fully discriminated from non-caprines (CVP = 100%). When applied to the archaeological specimens, the pLDA subsequently identified 173 archaeological specimens as caprines and 54 as non-caprines.

Within the caprine assemblage, pLDA assigned 105 specimens to goats and 68 to sheep (93 of pLDA iterations yielding CVP > 90%). Unsupervised and supervised results were highly consistent, with only eight specimens showing identification discrepancies between the two methods (supplementary material, table S1).

Iterative clustering of non-caprine specimens further resolved cattle, suids, gazelles and canids, consistent with modern reference groupings (supplementary material, table S1). Overall, 228 of 234 archaeological specimens were morphometrically identified as goats (*n* = 105), sheep (*n* = 68), cattle (*n* = 33), suids (*n* = 7), hominids (*n* = 7), gazelles (*n* = 5) and canids (*n* = 3), while six specimens remained unassigned and were retained as a mixed group.

Comparison with anatomical identifications revealed 16 discrepancies (supplementary material, table S1), most involving sheep-goat reassignments. Among the 19 previously unidentified specimens, morphometric analyses enabled confident taxonomic attribution for the majority (all except two): one cattle, six sheep, and 10 goats.

### 4.3. Interspecific comparison

Focusing on modern and archaeological cattle, sheep and goats (figure 3), cattle were clearly distinct from caprines in shape along PC1, whereas sheep and goats were clearly separated along PC2 (figure 3A). While the three species differed significantly in SC size (*p* < 2e-16), allometric relationships appeared consistent across species when modern and archaeological specimens were considered together (*p* = 0.174) and within each species between modern and archaeological assemblages (all *p* > 0.05). Shape changes associated with PC1 involved all three SCs structures, with cattle exhibiting rounder anterior and posterior SCs; while along PC2, shape variation was mainly localised on the posterior and anterior SCs, which were rounder and wider in sheep (figure 3A).

**Figure 3.**
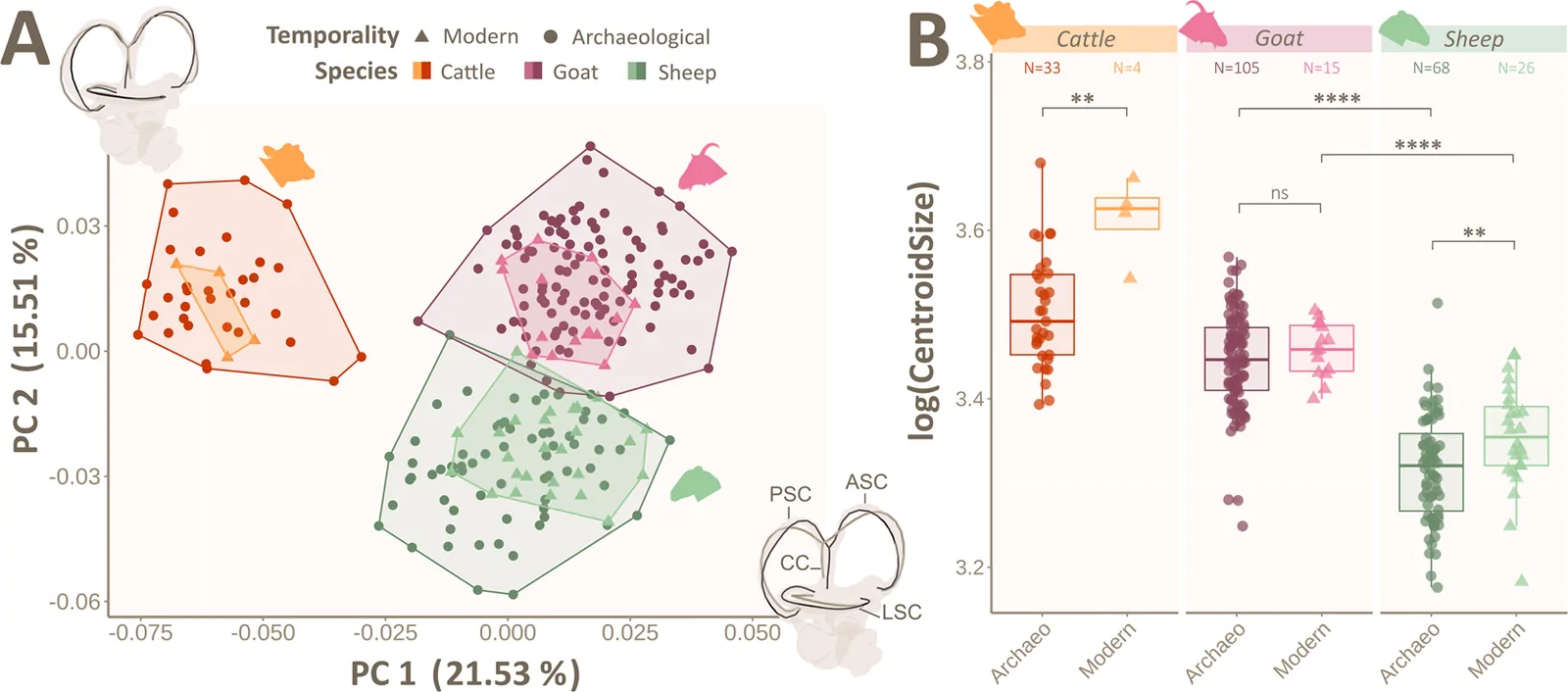
Interspecific differences in bony labyrinth morphology. (A) PCA of SCs shape variation among cattle, sheep and goats, with reconstructed extreme shapes along PC1 and PC2. Minimum and maximum PC scores are shown in light brown and black, respectively. (B) Log-transformed centroid size variation by species and specimen status (archaeological versus modern). Statistical significance of pairwise comparisons (Wilcoxon tests) is indicated: ns, not significant; **p≤ 0.01; ****p ≤ 0.0001. Abbreviations as in figure 2.

Modern and archaeological specimens largely overlapped within species and did not overlap among species except for two sheep exhibiting SC shapes resembling goats along the first two PCs. Shape variation along PC1 involved all three SCs structures, whereas PC2 primarily reflected variation in the posterior and anterior SCs (figure 3A).

For all three species, modern SCs' size variation fell within the archaeological range (figure 3B). Size comparisons revealed that archaeological cattle, all periods combined, had smaller SCs than modern cattle (*p* = 0.005; supplementary material, table S5; figure 3B), whereas no differences were detected between modern and archaeological goats (*p* = 0.5). Archaeological sheep exhibited significantly smaller SCs than modern sheep (*p* = 0.008). In both modern and archaeological samples, goats consistently displayed larger SCs than sheep (modern: *p* = 9.8e-7, archaeological: *p* = 6.8e-22).

No significant difference in mean shape was detected between archaeological and modern cattle (*p* = 0.973), whereas significant status differences in mean shape were observed in both goats (*p* = 0.002) and sheep (*p* = 0.001). All interspecific comparisons were highly significant regardless of the status (all *p* ≤ 0.001; supplementary material, table S6).

Morphological variance also differed among groups. Sheep exhibited the highest within-group variance (2.23e-3 in archaeological specimens; 1.92e-3 in modern specimens), whereas cattle showed the lowest values (1.62e-3 and 1.57e-3, respectively). Variance was significantly higher in archaeological than modern goats (*p* = 0.019), while no significant difference was detected between archaeological and modern cattle (*p* = 0.925) or sheep (*p* = 0.147; supplementary material, table S7). Allometric effects were significant and consistent across sheep and goats, with time-related allometry detected only in cattle (supplementary material, table S8).

Species-by-status (i.e., modern versus archaeological) size interactions were significant only in models including cattle (*p* = 0.024), indicating that the observed effect is primarily driven by cattle, which are represented by fewer specimens.

### 4.4. Diachronic study

Across periods, goats consistently exhibited larger SCs than sheep, with no significant species-by-period interaction (*p* = 0.8). The limited number of individuals per archaeological assemblage precludes robust inter-site comparisons within most periods, as some assemblages disproportionately dominate the samples; for example, Neolithic goats from Abdul Hosein account for 26 of the 47 specimens, Bronze Age sheep from Naderi for 11 of 37 and Bronze Age cattle from Aradetis Orgora for eight of the 19 specimens available for that period.

In cattle, the SCs size displayed substantial variability (figure 4A). Significant size reductions occurred between the Chalcolithic and the Bronze Age (*p* = 0.012) and between the Chalcolithic and the Iron Age (*p* = 0.016; figure 4B-1; supplementary material, table S9). This trend was followed by an increase between the Bronze Age and the modern period (*p* = 0.004) and between the Iron Age and the modern period (*p* = 0.016), although limited sample sizes restricted intermediate comparisons. No significant diachronic shape changes were detected in cattle (supplementary material, table S10).

**Figure 4.**
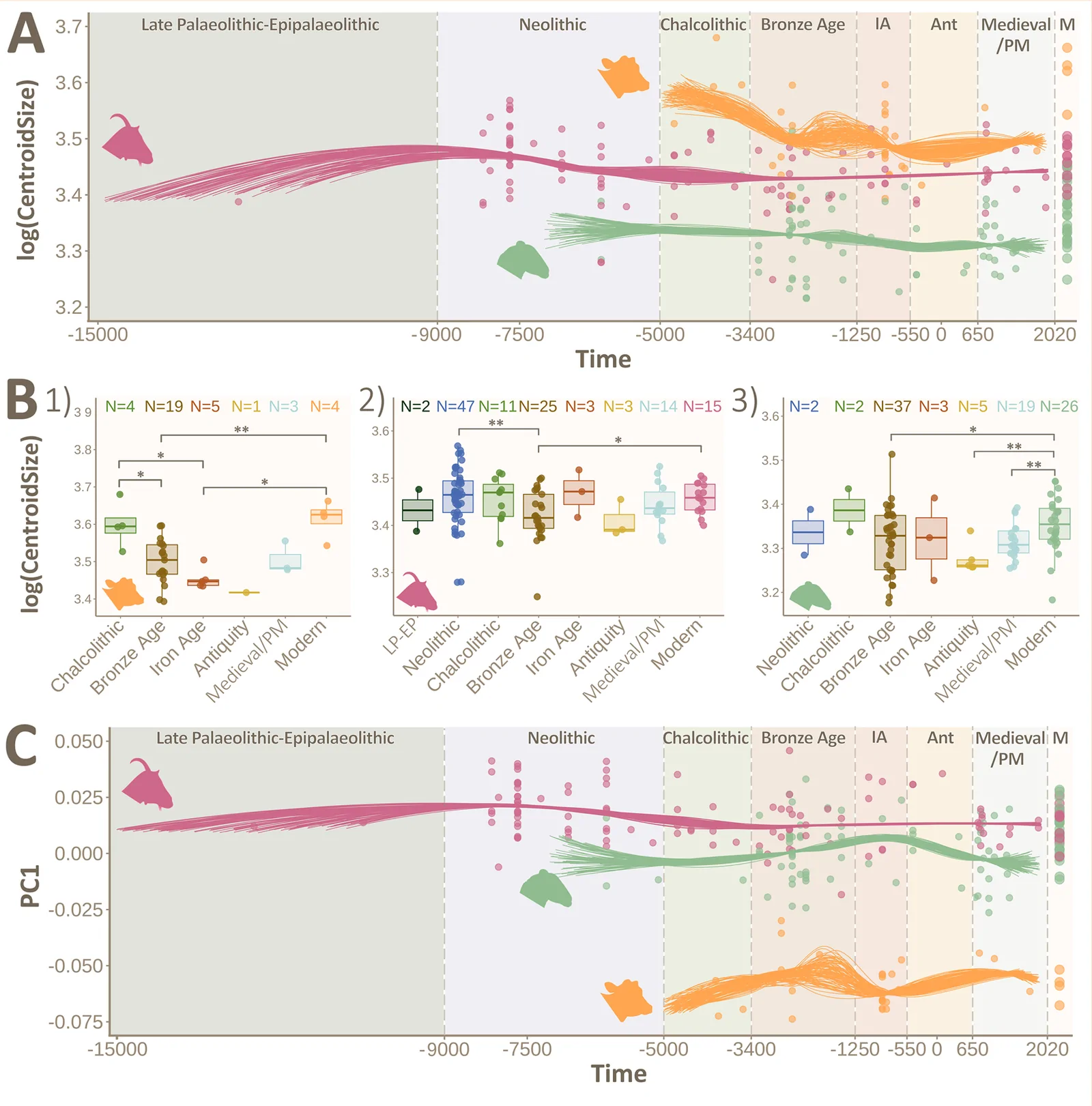
Diachronic variation in bony labyrinth size and shape. (A) Changes in log-transformed SC centroid size through time for the three species, with temporal uncertainty incorporated through 100 permutations. (B) SC centroid size distributions by period for cattle (1), goats (2) and sheep (3). (C) Shape variation through time along the first PCA axis for the three species, with 100 permutations. Period abbreviations: LP–EP, Late Palaeolithic–Epipalaeolithic; IA, Iron Age; Ant, Antiquity; PM, Pre-Modern; M, Modern. Statistical significance (Wilcoxon tests): ns, not significant; *p ≤ 0.05; **p ≤ 0.01.

In goats, the SCs size remained relatively stable through time (figure 4A and B). However, a significant decrease was detected during the Neolithic (*p* = 0.009, correlation = -0.38). Neolithic specimens had larger SCs than Bronze Age specimens (*p* = 0.004), and Bronze Age specimens exhibited smaller SCs than modern goats (*p* = 0.015; figure 4B-2). Shape differences among periods were significant (*p* = 1e-04), with all archaeological groups differing from modern goats except for the Epipalaeolithic (*p* = 0.4; supplementary material, table S10), with time explaining 12% of the total shape variance.

In sheep, SCs size of modern specimens was larger than in Bronze Age (*p* = 0.021), Antique (*p* = 0.009) and Medieval (*p* = 0.005) specimens (figure 4B-3), with no pairwise differences between the archaeological periods. Shape variation through time was significant (*p* = 1e-04), with 14% of total shape variance explained by time. Shape differences among periods were significant (*p* = 1e-04). Pairwise differences were significant, with modern sheep differing from the Bronze Age (*p* = 0.001), Antiquity (*p* = 0.004) and Medieval (*p* = 0.001) periods. Medieval sheep also differed from the Antiquity period (*p* = 0.05).

## 5. Discussion

This study provides the first large-scale geometric morphometric analysis of the bony labyrinth morphology in archaeological mammals, focusing on the Iranian Plateau and the Zagros, South Caucasus, South Central Asia and the Indus Valley, a key area for the emergence of animal domestication and the long-term development of herding practices. By combining three-dimensional GMM with an extensive archaeological and modern comparative framework, we reassessed species identifications, documented diachronic morphological patterns and explored evolutionary trajectories in sheep, goats and cattle across nearly 10 000 years of human-animal interactions.

### 5.1. Repeatability and sharing of morphometric datasets

A key methodological outcome of this study is the successful integration of morphometric datasets generated independently [48], made possible by low inter-operator landmarking error. Inter-operator variability is a well-recognised limitation in GMM [72–74], yet our results demonstrate that, when protocols are rigorously standardised, bony labyrinth landmarking yields reproducible and shareable data. This is particularly important for archaeological and paleontological contexts, where destructive sampling must be minimised, and where the long-term value of digital archives of heritage material extends beyond individual studies. The compatibility of independently produced datasets supports the development of cumulative reference collections and collaborative morphometric frameworks.

### 5.2. Bony labyrinth morphology as a taxonomic and evolutionary signal

The identification of a large number of archaeological individuals revealed a larger taxonomic diversity than initially thought. Bony labyrinth morphology carries a strong taxonomic signal in mammals, reflecting both phylogenetic history and functional constraints. In our modern comparative dataset, species or groups of species were clearly differentiated based on SC size and shape. Humans, in particular, were clearly separated from non-human taxa, notably owing to their more acute angle formed by their lateral SC with the *common crus*, a feature that may reflect differences in locomotion behaviour, head motion [75] or deeper phylogenetic divergence [76].

Importantly, the morphospace defined by modern non-human taxa fully encompassed the variation observed in archaeological specimens, allowing a robust taxonomic attribution for 228 of the 234 archaeological bony labyrinths analysed. This high identification success underscores the robustness of bony labyrinth morphometrics, even among closely related and morphologically similar taxa such as sheep and goats. This strong differentiation between sheep and goats was first recognised on modern specimens [48] that had been used to infer the species identification of archaeological specimens that were simultaneously identified through DNA sequencing [49]. The extended taxonomical coverage studied here provides a framework for taxonomic identification that could help select specimens prior to further analyses, such as DNA or isotopic studies.

Nonetheless, a small proportion of specimens (16 out of 234 specimens; approx. 7%) displayed inconsistencies between anatomical and morphometric identifications. Most discrepancies involved sheep-goat assignments, reflecting overlapping morphologies in ancient domestic caprines and pronounced regional and temporal variability. Such discrepancies may also reflect limitations in modern reference collections, which are exclusively European breeds and may not capture the full morphological diversity of ancient Southwest and Central Asian populations. The tendency for anatomically unassignable specimens to be classified morphometrically as goats contrasts with previous studies reporting perfect success rates but based on much smaller archaeological samples [77], suggesting that broader archaeological sampling reveals greater variability than previously recognised. Together, these findings emphasise the need to expand modern comparative datasets geographically to better represent the morphological diversity related to regional, ecological and husbandry practice variability and to refine diagnostic criteria to fully encompass the variability of medium-sized artiodactyls in the region.

One specimen (KK321) from Kafir Kala (Samarkand) clustered with modern humans, yet its cochlear morphology resembles that of a ruminant [78], and it has an unusually thick and short lateral SC. As petrous bones ossify *in utero* [79], these anomalies may represent developmental abnormalities rather than taxonomic distinctions. Further investigation, including cochlear-based analyses, will be necessary to resolve its attribution.

The relative proportion of sheep and goats varied across periods, consistent with archaeozoological studies showing evidence for shifting caprine management strategies, with the dominance of either sheep or goats being site- and period-dependent [20,80,81]. Neolithic assemblages such as Abdul Hosein were dominated by goats within mixed herds [82], whereas later periods in Central Asia and Sogdiana (present-day southern Uzbekistan and north-western Tajikistan) show a persistent preference for sheep from Antiquity through the Medieval period and into modern times [22,81,83]. These patterns reinforce the capacity of bony labyrinth morphometrics to recover meaningful economic and cultural signals from fragmentary archaeological remains.

### 5.3. Size differences between species and chronology

Across all periods, cattle exhibited larger SCs than sheep and goats. While this distinction is probably influenced by the larger body size of cattle, it also reflects broader taxonomic differences between bovines and caprines. Goats consistently exhibited larger SCs than sheep, regardless of chronology, a pattern observed in both modern bony labyrinth morphology [48,49] and external petrous bone measurements [59].

The functional interpretation of bony labyrinth variation, especially between closely related taxa, is complex. SCs play a key role in balance and head stabilisation, with larger canals enhancing sensitivity to angular movements and smaller canals dampening vestibular responsiveness, a trend often associated with differences in agility and locomotor style in primates [84]. Beyond overall size, canal morphology shows a degree of flexibility within species: shape can vary with locomotor behaviour even when dimensions remain constant, implying that subtle changes in geometry may influence vestibular performance among closely related individuals (e.g. [85]). The consistently larger SCs observed in goats may relate to their greater agility relative to sheep, supported by comparative studies on primates linking SC size and thickness with locomotor demands [75].

For the three species, archaeological specimens exhibited greater morphological variability than modern European reference specimens, a pattern that contrasts with much of the existing literature [28,45]. This discrepancy likely reflects the limited taxonomic and geographic breadth of the modern reference dataset, which includes only three to four breeds per species and only 45 individuals in total (with only four modern cattle). By contrast, archaeological assemblages studied here integrate multiple populations across wide temporal and spatial scales, capturing a broader spectrum of phenotypic diversity.

### 5.4. Diachronic trajectories

Sheep, goats and cattle display distinct diachronic trajectories in SC size, reflecting species-specific responses to domestication, subsequent selection and management practices.

Goat SCs show only a slight decrease in size from the Early Neolithic (circa 7500 BCE) to the Bronze Age, consistent with a size reduction associated with the domestication process in large mammals, often linked to selective breeding for desirable behavioural and reproductive traits [1].

Pronounced changes in horn morphology in both sheep and goats around 6000 BC during the late Neolithic further indicate the emergence of intensified livestock management in Southwest Asia and Northeast Africa [86,87]. Smaller animals may have been preferred for their increased docility and ease of adaptation to confined environments [88]. This size decline mirrors trends documented in the occidental Mediterranean basin on teeth [27] and overall body size [89]. Archaeozoological evidence from Iran also supports these findings, showing larger goats in the Neolithic than in subsequent periods [20,80,90].

In addition, the overall stability of goat bony labyrinth morphometrics for the rest of the chronology parallels genomic evidence indicating that early managed goat populations retained much of their wild morphology, despite clear genomic signatures of human management, including sex-biased culling [7].

By contrast, sheep display more pronounced temporal variability. Archaeological sheep display smaller SCs than modern specimens, with minimum sizes around the mid-first millennium BCE. This pattern is consistent with genomic evidence for rapid selection on traits such as coat colour, horn morphology and growth rate during the first two millennia of domestication [6]. Although SC shape variation through time was significant, limited sample sizes, particularly for early periods, likely constrain the detection of finer-scale temporal trends.

Cattle follow a distinct trajectory, with a marked decrease in SC size from the Chalcolithic through Antiquity, paralleling well-documented reductions in cattle body size across Southwest Asia and Northeast Africa during later prehistory [91]. This continuous decrease is consistent with regional archaeozoological evidence, linking cattle body size diminution to evolving husbandry practices from the Neolithic through at least 2000 BCE in the Levant and Anatolia [92], as well as the Iranian Plateau and the Zagros (e.g., [16]).

Beyond these broad diachronic patterns, however, it is important to acknowledge that size change in domesticates rarely unfolded uniformly across space. Archaeozoological data offer an important perspective: osteometric approaches, particularly log size index [93], highlight substantial size variability within the domestic herbivore triad at inter-regional and, in some cases, micro-regional scales. Such variability is documented throughout the long-term sequence from the Neolithic to the Medieval period (e.g., [19,83,94,95]). These findings suggest that broad diachronic trends in size reduction, including those observed here in SC metrics, likely coexisted with locally contingent management strategies and environmental constraints.

Together, these results emphasise that domestication did not impose a uniform morphological response across taxa. Instead, sheep, goats and cattle followed distinct evolutionary pathways shaped by species-specific biology and management strategies.

### 5.5. Archaeological integration and long-term evolutionary perspectives

Although sheep and goats are often herded together for similar purposes (e.g., meat, wool and milk) and share morphological and historical similarities, their evolutionary trajectories under human control diverged substantially. Archaeological evidence from the Iranian Plateau and adjacent regions documents a complex, nonlinear history of caprine domestication in both time and space (e.g. [16]), and our study corroborates this complexity through species-specific patterns of bony labyrinth size and shape variation.

Overall, the limited diachronic signal observed in cattle, goats and sheep likely reflects the sporadic nature of the available archaeozoological samples rather than an absence of biological change. The uneven distribution of specimens through time and space prevents, for now, site-specific interpretations and precludes inter-site or regional comparisons (supplementary material, figure S4).

## 6. Conclusions and perspectives

This study demonstrates that three-dimensional GMM of the bony labyrinth provides a robust, non-destructive and reproducible framework for species identification and diachronic analysis of archaeological petrous bones. Applied to a large dataset from the Iranian Plateau, the Zagros, the South Caucasus, South Central Asia and the Indus Valley, the approach revealed a broader taxonomic diversity than previously recognised and established a reusable morphometric reference framework for the identification of archaeological specimens that are difficult to assign using petrous bone anatomy alone. In particular, it enabled robust discrimination between sheep and goat and clarified species attribution across a wide range of archaeological contexts.

By integrating modern and archaeological material, this study reveals distinct, species-specific evolutionary trajectories in sheep, goats and cattle over nearly 10 000 years of human–animal interaction. Differences in SC size and shape likely reflect a combination of functional constraints and domestication-related processes, highlighting the complex and nonlinear phenotypic responses of domestic taxa to changing husbandry practices. The preservation of three-dimensional digital archives of the bony labyrinth further enhances the long-term value of petrous bones and facilitates integration with osteological, morphometric and palaeogenetic datasets.

Conducted in the cradle of domestication and long-standing agro-pastoral practices, this study sheds new light on the evolutionary history of key domestic species. While the biological and cultural drivers of bony labyrinth variation remain difficult to disentangle, potential influences include altered locomotor regimes associated with captivity [96] or broader cranial reorganisation during domestication, as documented in other taxa [97,98]. Future studies integrating extended morphometric as well as palaeogenetic data will be essential to test these hypotheses.

More broadly, this study underscores the importance of long-term, collaborative and interdisciplinary research grounded in extensive fieldwork for reconstructing the deep history of human–animal relationships. By combining large-scale archaeological sampling with advanced morphometric and genetic approaches, it provides a framework for future diachronic and micro-regional investigations aimed at refining our understanding of the biological, functional and cultural drivers of domestic animal evolution over the longue duree.

## Supplementary Material



## Data Availability

The data and the code are available at InDoRES [99].
