# Supplementary material for: Bony labyrinth taxonomic identification and long-term evolution of livestock in the Southwest and Central Asia
Source: R Soc Open Sci. 2026 Oct 7;13(10):261493. doi: 10.1098/rsos.261493 (PMC13641910; doi:10.1098/rsos.261493)
Supplement: Supplementary file 1 [file rsos.261493.zip › rsos.261493/rsos261493_si_001.docx]

### Inner Ear Taxonomic Identification and Long-Term Evolution

### of Livestock in the Southwest and Central Asia

### *Supplementary Information*

#### *Summary*

##### **SI_Text 1.** Chronological overview of environmental change and Neolithic expansion across the Zagros and Alborz regions.

##### **SI_Text 2.** Regional sample coverage and cultural context from the Late Palaeolithic/Epipalaeolithic to the Medieval period in Southwest and Central Asia.

##### **SI_Table 1**. Dataset of individual samples with archaeozoological and geometric morphometrics identification, as well as clustering identifications for caprines. ID: identification; ArchZoo: archaeozoological; GMM: geometric morphometrics; TPQ: *terminus post quem*; TAQ: *terminus ante quem*.

##### **SI_Table 2.** Names of the 51 archaeological and modern sites investigated in the studied area (Azerbaijan, Georgia, Iran, Iraq, Pakistan, Türkiye and Uzbekistan), their regional attributions, the associated archaeological specimen counts (N), and the site identifiers for the map (ID, SI_Figure 1).

##### **SI_Text 3.** Description of Study Sites. The number before each site refers to the number reported on SI_Figure 1 and SI_Table 2. The coordinates of the sites are given next to the name of the site in the geographic coordinate system (WGS 84), with latitude listed first (North) and longitude second (East).

##### **SI_Table 3.** General chronological framework of studied areas (BCE: Before Common Era).

##### **SI_Figure 1.** Geographical distribution of the 51 archaeological sites analysed. Dots indicate site locations and are coloured according to associated periods. Dot size is proportional to the number of specimens analysed per site, with the names of the larger assemblages mentioned in the study. The inset shows the broader geographical context of the study area. LP-EP: Late Palaeolithic-Epipalaeolithic; Ch: Chalcolithic; BA: Bronze Age. For information on the sites, refer to SI_Table 2.

##### **SI_Figure 2.** Detailed protocol for measuring the bony labyrinth according to Evin et al. (2022): A) identification of different structures; B) placement of reference points along the semicircular canals (SCs); C) isolation of the 4 structures of interest (ASC: anterior semicircular canal; PSC: posterior semicircular canal; CC: common crus; LSC: lateral semicircular canal); D) superimposition of the 3 common points at the CC; E) adjustment of points along the curves; F) visualisation of the obtained curves; G) placement of sliding semilandmarks (in red) along the curves; 16 points on the ASC, 16 on the PSC, 5 on the CC, 23 on the LSC, landmarks are shown in green; H) visualisation of coordinates in RStudio. Landmark (LM) 1: Midpoint of the anterior ampulla; LM 18: Midpoint of the common crus, where the anterior and posterior semicircular canals meet; LM 24: Lower midpoint of the common crus (juncture with the vestibule); LM 41: Midpoint of the posterior ampulla; LM 42: Midpoint of the end of the lateral semicircular canal (juncture with the vestibule); LM 66: Midpoint of the lateral ampulla.

**SI_Table 4.** Relative measurement error of replica measurements. Intra- and inter-operator errors in size and shape measurements were calculated for each operator according to Claude (2008).

**SI_Text 3.** Morphometric identification of archaeological specimens detailed. Supervised (pLDA) and unsupervised (clustering) analyses were used to assign archaeological specimens to taxonomic groups based on modern reference datasets.

**SI_Table 5**. Wilcoxon test results for pairs of centroid size means (cattle, sheep and goats). Significance of the p-values is indicated by the asterisks and means that the sizes are different. ns: not significant, *: p ≤ 0.05, **: p ≤ 0.01, ***: p ≤ 0.001, and ****: p ≤ 0.0001.

**SI_Table 6.** Mean shape comparisons among cattle, goats, and sheep. Pairwise Procrustes-based tests of mean shape distances between modern and archaeological populations. Significance of the p-values is indicated by asterisks and means that the shape variances are different. ns: not significant, *: p ≤ 0.05, **: p ≤ 0.01, ***: p ≤ 0.001, ****: p ≤ 0.0001.

##### **SI_Table 7.** Morphological variance comparisons among cattle, goats, and sheep. Pairwise Procrustes-based tests of variance differences between modern and archaeological populations. Significance of the p-values is indicated by asterisks and means that the shape variances are different. ns: not significant, *: p ≤ 0.05, **: p ≤ 0.01, ***: p ≤ 0.001, ****: p ≤ 0.0001.

##### **SI_Table 8.** Allometry analysis for cattle, goats and sheep. Results of Procrustes ANOVAs testing the existence and homogeneity of allometries between modern and archaeological populations. Significance of the p-values is indicated by the asterisks and testifies to the robustness of the relationships tested. ns: not significant, *: p ≤ 0.05, **: p ≤ 0.01, ***: p ≤ 0.001.

##### **SI_Table 9.** Wilcoxon test results for pairs of log-transformed centroid sizes for each period (cattle, sheep and goats). Significance of the p-values is indicated by the asterisks and means that the sizes are different. ns: not significant, *: p ≤ 0.05, **: p ≤ 0.01, ***: p ≤ 0.001, and ****: p ≤ 0.0001.

##### **SI_Table 10.** Pairwise Procrustes-based tests of shape distances between each pair of periods (cattle, sheep and goats). Significance of the p-values is indicated by the asterisks. ns: not significant, *: p ≤ 0.05, **: p ≤ 0.01, ***: p ≤ 0.001.

**SI_Figure 3.** Visualisation of differences between SC measurements of three BMC sheep (Blanche du Massif Central breed) by operators AH (A. Hemelsdaël) and AG (A. Guignet). (A) PCA of the first two axes; (B) boxplot of the log-transformed centroid size.

##### **SI_Figure 4.** Distribution of the three taxa over time (TPQ-TAQ, in years – if available) and according to archaeological sites.

##### **SI_Figure 5.** Bony labyrinth morphometric overall variation. (A) Principal Component Analysis (PCA) of semicircular canals (SCs) shape variation along the PC1 and PC3. (B) PCA of SCs shape variation along the PC2 and PC3.

**SI_Table 11.** Detailed authors’ contributions.

**References**

Claude J (2008) *Morphometrics with R*. Springer, New York.

Evin A, David L, Souron A, Mennecart B, Orliac M, Lebrun R (2022) Size and shape of the semicircular canal of the inner ear: A new marker of pig domestication? *Journal of Experimental Zoology Part B: Molecular and Developmental Evolution*, **338**, 552–560.<https://doi.org/10.1002/jez.b.23127>
