# Supplementary material for: Bony labyrinth taxonomic identification and long-term evolution of livestock in the Southwest and Central Asia
Source: R Soc Open Sci. 2026 Oct 7;13(10):261493. doi: 10.1098/rsos.261493 (PMC13641910; doi:10.1098/rsos.261493)
Supplement: Supplementary file 1 [file rsos.261493.zip › rsos.261493/rsos261493_si_018.docx]

##### **SI_Text 1**. Chronological overview of environmental change and Neolithic expansion across the Zagros and Alborz regions.

Between ~48,000 and 16,000 BP, the Zagros region experienced very cold, dry conditions with Irano-Turanian dry mountain steppe vegetation and largely treeless uplands, aside from riparian tamarisk, willow, and sea buckthorn (van Zeist & Bottema 1977; Djamali et al. 2008a,b; Wasylikowa et al. 2008). Conditions briefly ameliorated in the Late Glacial Interstadial (~14,500 BP) but remained relatively arid, with full warming and broader tree spread only in the Early Holocene; trees retreated during the Younger Dryas and expanded again by ~9000 BCE alongside early goat and cereal domestication (Wasylikowa et al. 2008). Throughout the Holocene, the landscape featured mosaics of wild pistachio and almond scrubs with steppes until oak woodlands expanded from ~4500 BCE—driven by shifts in precipitation seasonality—and modern open deciduous oak/pistachio/almond woodlands peaked in the mid-4th millennium BC (Stevens et al. 2006; Roberts 2002; Djamali et al. 2010; Wasylikowa et al. 2008; Sharifi et al., 2023). By contrast, central Alborz records (last ~20,000 years) show stronger forest expansion during the Late Glacial Interstadial and an immediate oak expansion in the early Holocene—likely due to proximity to the Hyrcanian refugium—so Alborz mountain areas were forested from the start of the Holocene (Ramezani et al. 2023).

Today, the Iranian Plateau, situated in southwest Asia at the eastern fringes of the Fertile Crescent, comprises approximately nine geographical-archaeological/cultural distinct zones: North (Alborz Mountains and the southern Caspian Sea coast), Northwest (northern Zagros Mountains), West (central Zagros), Southwest (lowland plains of Khuzestan and Ilam), South (southern Zagros and northern coasts of the Persian Gulf), Southeast (southeast mountains of the Central Iranian Range), East (eastern margin of the Kavir Desert/Helmand basin), Northeast (Alborz and Kopet Dagh ranges), and the Central Plateau (Malek Shahmirzadi, 1999). These zones differ to some extent in topography, hydrology, and biotic resources, factors that have shaped human subsistence and settlement patterns through the Pleistocene and Holocene. The climate could be generally divided into the following regimes: temperate-humid (the Caspian Sea coasts), cold (Zagros and the Alborz mountains), hot-dry (much of the Central Plateau) and hot-humid (coastal strips along the Persian Gulf and Makran Sea) (Ganji, 1954; Beck et al., 2018).

The Zagros and Alborz mountain systems provided refugia and habitats for the wild progenitors of domesticated animals and cultivated plants, and their environmental heterogeneity and resource diversity created favourable conditions for the emergence of agriculture and herding in the early Holocene. Current archaeological evidence indicates that Neolithisation in this region began in the intermontane plains of the Central Zagros and subsequently spread northward, eastward and southward during the mid‑eighth millennium BCE. In other parts of Iran, notably the Central Plateau, the southeast and the eastern margins, Neolithic lifeways became established somewhat later, by the end of the seventh millennium BCE (Late Neolithic). From the Late Neolithic onwards, agro‑pastoral economies were present across all regions of the Iranian Plateau and persisted, with regional variation, into modern times (Mashkour and Davoudi, 2024). The archaeological sample used in this study intentionally spans the major geographical–archaeological zones of Iran and includes contemporaneous sites from several neighbouring regions such as Anatolia, the Caucasus, Upper Mesopotamia, and Central and South Asia. This coverage permits a long‑term assessment of livestock species composition and trajectories from the Early Neolithic (e.g., Abdul Hosein and Jarmo) through to the Late Islamic period (e.g., Kakh‑e Jahannama).

**References**

Beck HE, Zimmermann NE, McVicar TR, Vergopolan N, Berg A, Wood EF. 2018 Present and future Köppen-Geiger climate classification maps at 1-km resolution. *Sci Data* 5, 180214. (doi:[10.1038/sdata.2018.214](https://doi.org/10.1038/sdata.2018.214)).

Djamali M, Kürschner H, Akhani H, de Beaulieu J.-L, Amini A, Andrieu-Ponel V, Ponel P, Stevens L. 2008b Palaeoecological Significance of the Spores of the Liverwort Riella (Riellaceae) in A Late Pleistocene Long Pollen Record from the Hypersaline Lake Urmia, NW Iran. *Review of Palaeobotany and Palynology* 152, no. 1-2: 66-73.<https://doi.org/10.1016/j.revpalbo.2008.04.004>.

Djamali M, Ponel P, Delille T, Thiéry A, Asem A, Andrieu-Ponel V, de Beaulieu J.-L, Lahijani H, Shah-Hosseini M, Amini A, and Stevens L. 2010 A 200,000-Year Record of the Brine Shrimp Artemia (Crustacea: Anostraca) Remains in Lake Urmia, NW Iran. *International Journal of Aquatic Science* 1, no. 1: 14-18.

Djamali M, Ponel P, Delille T, Thiéry A, Asem A, Andrieu-Ponel V, de Beaulieu J.-L, Lahijani H, Shah-Hosseini M, Amini A, and Stevens L. 2010 A 200,000-Year Record of the Brine Shrimp Artemia (Crustacea: Anostraca) Remains in Lake Urmia, NW Iran. *International Journal of Aquatic Science* 1, no. 1: 14-18.

Ganji MH. 1954 Climate classification of Iran. *Journal of Faculty of Literature and Humanities of University of Tehran* 1, 27–72.

Mashkour M, Davoudi H. 2024 Ambushing the Wild and Following the Herds: Archaeozoological Review of Human Occupation of the Iranian Mountains from the Middle Palaeolithic to the Late Neolithic. In: The Oxford Handbook of Mountain Archaeology (eds Carrer F, Callanan M, Della Casa P, Fontana F, Reinhold S, Saul H), Oxford University Press. <https://doi.org/10.1093/oxfordhb/9780197608005.013.15>

Malek Shahmirzadi, S. 1999 *Prehistoric Iran: The Archaeology of Iran from the Earliest Times to the Down of Urbanism*. Tehran: Sobhan Noor Publications.

Ramezani E, de Klerk P, Naqinezhad A, Theuerkauf M, and Joosten H. 2023 Long-Term Dynamics of Oriental Beech (*Fagus orientalis* Lipsky) Stands in the Hyrcanian Forests of Northern Iran. *Review of Palaeobotany and Palynology* 312: 104871.<https://doi.org/10.1016/j.revpalbo.2023.104871>.

Roberts N. 2002 Did Prehistoric Landscape Management Retard the Post-Glacial Spread of Woodland in Southwest Asia?. *Antiquity* 76, no. 294: 1002-1010.<https://doi.org/10.1017/S0003598X0009181X>.

Sharifi A, Djamali M, Peterson LC, Swart PK, Guadalupe Pulido Ávila M, Esfahaninejad M, de Beaulieu J.-L, Lahijani H, and Pourmand A. 2023 The Rise and Demise of Iran’s Urmia Lake during the Holocene and the Anthropocene: “What’s Past is Prologue. *Regional Environmental Change* 23, no. 4: 121. https://doi.org/10.1007/s10113-023-02119-x.

Stevens L, Ito E, Schwalb A, and Wright HE. 2006 Timing of Atmospheric Precipitation in the Zagros Mountains Inferred from a Multi-Proxy Record from Lake Mirabad, Iran. *Quaternary Research* 66, no. 3: 494-500.<https://doi.org/10.1016/j.yqres.2006.06.008>.

Van Zeist W, Bottema S. 1977 Palynological Investigations in Western Iran. *Palaeohistoria* 19: 19-85. <https://ugp.rug.nl/Palaeohistoria/article/view/25018>.

Wasylikowa K, van Zeist W, Wright HE, Stevens LR, Witkowski A, Walanus A, Hutorowicz A, Alexandrowicz SW, and Langer JJ. 2008 The Lake Zeribar Palaeoecology: A Synthesis. In The Palaeoecology of Lake Zeribar and Surrounding Areas, Western Iran, During the Last 48,000 Years, Diatom Monographs 8, K. Wasylikowa and A. Witkowski (eds.): 303-321. Ruggel: A.R.G. Gantner Verlag K.G.
