# Supplementary material for: Bony labyrinth taxonomic identification and long-term evolution of livestock in the Southwest and Central Asia
Source: R Soc Open Sci. 2026 Oct 7;13(10):261493. doi: 10.1098/rsos.261493 (PMC13641910; doi:10.1098/rsos.261493)
Supplement: Supplementary file 1 [file rsos.261493.zip › rsos.261493/rsos261493_si_019.docx]

##### **SI_Text 2.** Regional sample coverage and cultural context from the Late Palaeolithic/Epipalaeolithic to the Medieval period in Southwest and Central Asia.

*Regional sample coverage*

The samples analysed in this study derive predominantly from the Iranian Plateau (156 samples) and encompass the full geographical extent of modern Iran, spanning a broad chronological range from the Late Palaeolithic to the present, with a particular emphasis on the Zagros regions (e.g., for the Neolithic, Abdul Hosein and Jarmo/Charmo, and for the Medieval period, Masjed-e Jame' Isfahan). This geographical coverage is complemented by a sample from an early Neolithic site located in the northwestern foothills of the Zagros Mountains.

The Caucasus, Southern Central Asia, and the Indus Valley are each represented by smaller sample sets, which nonetheless account for 78 of the 234 specimens analysed in the study. The Caucasus constitutes a substantial component of the dataset, with samples originating from five archaeological sites in present-day Georgia, spanning the Late Chalcolithic, Early Bronze Age, Iron Age, and Medieval periods (Rova *et al*., 2010; Gambashidze, 2021; Sauer *et al*., 2020; Stöllner *et al*., 2023; Vautrin, 2025), together with one Early Chalcolithic site from Azerbaijan (Marro, 2022). A single sample from Anatolia, dating to the Chalcolithic and Bronze Ages, is also included (Berthon & Mashkour, 2008). In southern Central Asia, samples originate from archaeological sites located in the Zeravshan Valley (modern-day Uzbekistan), more specifically from the oases of Samarkand and Bukhara, and date back to Antiquity and the Early Medieval period (Decruyenaere *et al*., 2024; Begmatov et al., 2024). Finally, the Indus Valley is represented by a single sample recovered in Sindh, dating to the Early Bronze Age (Didier *et al*. 2017, 2024).

##### *Cultural context*

The Epipaleolithic of Iran dates approximately from 20,000 BP to 9,500 BC and represents a transitional cultural phase between the Palaeolithic and Neolithic periods, preceding the onset of domestication. In the Zagros Mountains, this period is commonly referred to as the Zarzian culture, while in the Alborz Mountains it is generally described as Mesolithic (Hole & Flannery, 1968; Richter & Darabi, 2023). Microlithic technologies dominate lithic assemblages and include scrapers, notches, borers, denticulates, backed blades, microblades, and notably geometric microliths, which are diagnostic of the period (Olszewski, 2012).

Southwest Asia was one of the earliest centres of plant and animal domestication (9,000-8,000 cal. BCE), particularly involving caprines, cattle, pigs, barley, wheat, and lentils (e.g., Peters *et al*., 2005; Riehl *et al*., 2013; Zeder, 2024). The samples analysed in this study derive from regions within this core area, notably present-day eastern Türkiye and the Zagros Mountains of Iran and Iraq. During the Neolithic, human communities across these regions developed distinct husbandry strategies, varying in the management and relative proportions of domesticated species, reflecting diverse local trajectories of plant and animal exploitation (Riehl *et al*., 2013; Daly *et al*., 2018; Richter & Darabi, 2023).

Quickly, the Neolithic lifestyle, including notably domesticated species and technologies, spread outward along multiple pathways. In the Caucasus, the Neolithic occupations are attributed to the end of the 7th mill. BCE, with probably no local domestication (e.g., Lyonnet, 2007; Marro *et al*., 2019; Nishiaki *et al*., 2019; see a review in Vautrin et al., accepted). Between northeastern Iran and the western regions of southern Central Asia, archaeological evidence points to cultural and economic connections. Neolithic sites such as Jeitun in the Kopet Dag (modern Turkmenistan; Harris, 2010) and Sang-e Chakhmaq (Iran, Roustaei *et al*., 2015) illustrate shared agro-pastoral practices in the beginning of the 6th mill. BCE. (Roustaei, 2016). As for Central Asia, Taylor *et al.* (2021), based on aDNA analyses, further propose a possible eastward dispersal toward the Fergana Valley (modern Kyrgyzstan) as early as 6000 BCE.

At the beginning of the 5th millennium BCE, the Chalcolithic was associated with the development of a more complex exchange network. Obsidian from the Caucasus has been found in the Lake Urmia region of Iran and in the Persian Gulf, while lapis lazuli from Afghanistan has been discovered in both Iran and the Caucasus (Chataigner *et al*., 2010; Lyonnet *et a*l., 2008; Marro *et al*., 2018; Marro, 2022). This long-distance exchange can be observed in Central Asia, notably through the mobility of turquoise stones discovered throughout the steppes and beyond (Castro, 2024). Metallurgy is also developed during this period (Thornton, 2009; Sagona, 2018). Archaeological evidence points to the emergence of more integrated cultural formations across neighbouring zones. During this period, economic ties tended to be strongest between different areas; for instance, in northeastern Iran and southwestern Turkmenistan, Baluchistan in southeastern Iran and western Pakistan, and the southwestern and western lowlands of Iran with southern Mesopotamia, and northwest Iran with the Caucasus.

In the Bronze Age (from the middle of the 4th millennium to the middle/end of the 2nd millennium BCE), processes of urbanisation and population growth increased aggregate demand for animal products (Arbuckle, 2012; Arbuckle and Hammer, 2018). This heightened demand, together with diverse local environments and human requirements, contributed to the selective breeding and possibly to the regional differentiation of sheep, goats and cattle. The domestication of donkeys, horses, and two-humped camels from the Late Neolithic through the Bronze and Iron Ages had profound implications for mobility and interregional interaction. The North Caucasus and Central Asia, in particular, were key regions for the domestication of horses (Librado *et al*., 2021) and Bactrian camels (Suska-Malawska *et al*., 2022). These species facilitated longer-distance migrations, more efficient movement of people and commodities, and the expansion of trade networks, thereby accelerating cultural transmission and economic integration across wide areas (Meyer *et al*. 2019). Trade networks were crucial in connecting distant regions. One major route extended from Mesopotamia eastwards, passing through northern Iran before reaching Turkmenistan and connecting to Bactria. In the later periods, this route, known as the Khorasan Road (Gopnik and Rothman, 2011), will be one of the main arteries of the Silk Roads. Another important trade network, active during the Bronze Age, connected the regions of present-day Pakistan and southern Afghanistan to southeastern, southern, and southwestern Iran, eventually reaching southern Mesopotamia. A third route linked the Caucasus and parts of southern and southeastern Türkiye to northwestern Iran. Several archaeological sites examined in this study, Hasanlu, Alou, Chalow, Naderi, Kale Kub, Graziani, Chanhu-daro, etc., are associated with these cultural and trade interactions.

The Late Bronze Age and Iron Age are often regarded as the proto-historic period (Danti, 2013). The emergence of local and regional governments during this period laid the foundation for the historical era and the rise of major political entities, including the Babylonian and Assyrian empires, the kingdom of Urartu, and the Median polity (Muscarella, 2006).

This process culminated with the emergence of the Achaemenid Persian Empire (550–330 BCE), which, at its greatest extent, encompassed a vast territory stretching from Anatolia and the southeastern Mediterranean region in the west to the Indus area in the east. As part of this expansion, the Achaemenids launched military campaigns into part of Central Asia (Baumer, 2014; Gershevitch, 1985).

During the Antiquity era, as well as throughout the Medieval era (Milwright 2010), medium- and long-distance trade and exchange intensified further with the development of new routes, notably eastward extensions of the “Khorasan Road” linking Iran and Merv to Bukhara (Wordsworth, 2024; Rante et al., 2016), which provided alternative pathways onwards to China. Within this framework, Sogdian merchants played a decisive role by establishing trading colonies from Sogdiana to China and by controlling commerce along these routes, now collectively referred to as the Silk Roads (de la Vaissière, 2002; Duturaeva, 2025).

These expanding networks linking Europe to China, passing through the Iranian world, fostered a growing openness to the wider world, stimulating economic development and cultural interaction (Hansen, 2017). These exchange networks between various populations, sedentary or nomadic, enabled not only the circulation of goods and livestock but also the transmission of knowledge and practices, including techniques related to animal husbandry and pastoral strategies. In doing so, they profoundly shaped the way of life in urban centres, contributing to their long-term growth and sustainability.

**References**

Arbuckle (2012) Pastoralism, Provisioning, and Power at Bronze Age Acemhöyük, Turkey. American Anthropologist 114:3, 462-476.

Arbuckle BS, Hammer EL (2019) The Rise of Pastoralism in the Ancient Near East. Journal of Archaeological Research, 27, 391–449. <https://doi.org/10.1007/s10814-018-9124-8>

Baumer C. (2014) The History of Central Asia (Volume Two): The Age of the Silk Roads, I.B. Tauris, London. ISBN 978-1-78076-832-8

Begmatov A, Teramura H, Decruyenaere D, Mashkour M, Mir-Makhamad B, Spengler R, Murakami T, Sandiboev A, Takao U (2024) The Grand Hall of Kafir-Kala: An Observation of Archaeological Finds, Botanical and Zoological Evidence. History and Archaeology of Turan, Texts in honor of Bogomolov G. & Berdimuradov A., Samarkand Institute of Archaeology, 6, 194–206.

Berthon RJ-P, Mashkour M. (2008). Animal Remains from Tilbeşar Excavations, Southeast Anatolia, Turkey. *Anatolia antiqua. Eski Anadolu* 16, 23–51. (doi:[10.3406/anata.2008.1248](https://doi.org/10.3406/anata.2008.1248))

Castro, M. (2024) New Research on Turquoise Stones in Pre-Modern Central Eurasia. In Silk Roads Papers, 1st Edition, Paris: UNESCO, 51—65.

Chataigner C. Avetisyan P., Palumbi G., Uerpmann H.-P. (2010). Godezor, a late Ubaid-related settlement in the Southern Caucasus. In: Carter R. A., Philip G. (eds.). Beyond the Ubaid. Transformation and integrism in the late prehistoric societies of the Middle-East. The Oriental Institute of the University of Chicago, Chicago, 381-398.

Daly KG, Maisano Delser P, Mullin VE, Scheu A, Mattiangeli V, Teasdale MD, Hare AJ, Burger J, Verdugo MP, Collins MJ, Kehati R, Erek CM, Bar-Oz G, Pompanon F, Cumer T, Çakırlar C, Mohaseb AF, Decruyenaere D, Davoudi H, Çevik Ö, Rollefson G, Vigne J-D, Khazaeli R, Fathi H, Doost SB, Rahimi Sorkhani R, Vahdati AA, Sauer EW, Azizi Kharanaghi H, Maziar S, Gasparian B, Pinhasi R, Martin L, Orton D, Arbuckle BS, Benecke N, Manica A, Horwitz LK, Mashkour M, Bradley DG (2018). Ancient goat genomes reveal mosaic domestication in the Fertile Crescent. Science, 361, 85–88. <https://doi.org/10.1126/science.aas9411>

Danti MD. 2103 The Late Bronze and Early Iron Age in northwestern Iran. In: DT Potts (ed.), The Oxford Handbook of Ancient Iran, Oxford University Press: 327-376.

# de La Vaissière E. (2002) *Histoire des marchands sogdiens*. Paris, Collège de France, Institut des Hautes Études Chinoises, 413 p.

Decruyenaere D, Rante R, Vuillien M, Mashkour M (2024) Animal Remains in the Oasis of Bukhara: Preliminary Results. In: The Oasis of Bukhara Volume 3: Material Culture, Socio-Territorial Features, Archeozoology and Archaeometry Arts and Archaeology of the Islamic World. (ed Rante R), pp. 364–370. Brill, Leiden.

Didier A, Castillo DS, Mongne P, & Shah SSA. 2017 Resuming excavations at Chanhu-daro, Sindh: First results of the 2015-2017 field-seasons. *Pakistan Archaeology* 30: 69-121.

Didier A, Marchand F, Sarmiento-Castillo D. 2024. Les lapidaires de Chanhu-daro (Sindh, Pakistan). Logiques productives et organisation socio-économique dans un centre urbain majeur de l’Âge du bronze en Asie du Sud. C. Cheval, O. Langlois, M. Lauwers, G. Palumbi, H. Procopiou (eds.)*, Acteurs techniques, acteurs sociaux. Des vestiges matériels à l’organisation sociale du travail, de la Préhistoire à nos jours*. 43es rencontres internationales d’archéologie et d’histoire – Nice Côte d’Azur, Éditions APDCA: 89-102 (2-904110-66-6. ff10.4000/14ys6ff).

Duturaeva D. 2025 Silk Roads before the Qarakhanids: Étienne de la Vaissière’s Asie centrale. Journal of Central Asian History 4(2): 287-294. <https://doi.org/10.1163/27728668-20250040>

Gambashidze I. 2021 Radiocarbon Dates from the Orchosani Settlement and the Chalcolithic/Early Bronze Age Chronology of Samtskhe (South-West Georgia). In: L. Giemsch and S. Hansen (eds). *The Caucasus, bridges between the urban centres in Mesopotamia and the Pontic steppes in the 4th and 3rd millenium BC. The transfer of knowledge and 229 technologies between East and West in the Bronze Age*. Schriften des Archäologischen Museums Frankfurt, Regensburg: Verlag Schnell & Steiner GmbH: 133–158.

Gershevitch I. (ed.). 1985 *The Cambridge History of Iran*. Cambridge University Press.

Gopnik H, and Rothman MD. 2011 On the high road: the history of Godin Tepe, Iran. Costa Mesa CA, Mazda Publishers, Bibliotheca Iranica / Archaeology, Art and Architecture Series.

Harris DR. 2010 Origins of Agriculture in Western Central Asia: an Environmental-Archaeological Study. University of Pennsylvania Press. <https://doi.org/10.9783/9781934536513>

Hansen HB, Damgaard PB, Margaryan A, Stenderup J, Lynnerup N, Willerslev E, Allentoft ME (2017) Comparing Ancient DNA Preservation in Petrous Bone and Tooth Cementum. PLoS One, 12, e0170940. <https://doi.org/10.1371/journal.pone.0170940>

Hole F, & Flannery K.V. 1968 The prehistory of southwestern Iran: a preliminary report. In *Proceedings of the Prehistoric Society* 33: 147-206.

Insoll, T. 1996 Islam, archaeology and history. *Gao Region (Mali) ca. ad*.

Librado P, Khan N, Fages A, Kuslyi M, Suchan T, Tonasso-Calvière L, Schiavinato S, Alioglu D, Fromentier A, Perdereau A, Aury JM, Gaunitz C, Chauvey L, Seguin-Orlando A, Sarkissian C, Southon J, Shapiro B, Alquraishi S, Alfarhan AH, Al-Rasheid KAS, Seregely T, Klassen L, Iversen R, Bignon-Lau O, Bodu P, Olive M, Castel JC, Alvarez N, Germonpré M, Wilczyński J, Rannamäe E, Saarma U, Lōugas L, Kyselý R, Peške L, Balasescu A, Gerber D, Kulcsár G, Gál E, Bendrey R, Allentoft M, Shephard H, Tomadini N, Grouard S, Pitulko V, Brem G, Wallner B, Keller M, Kitagawa K, Bessudnov A, Taylor W, Bayarsaikhan J, Erdenebaatar D, Kubatbeek T, Mijiddorj E, Boldgiv B, Tsagaan T, Pruvost M, Olsen S, Makarewicz C, Valenzuela Lamas S, Albizuri S, Nieto Espinet A, Lira Garrido J, Kotova N, Pryor A, Crabtree P, Zhumatayev R, Kuznetsova T, Lordkipanize D, Marzullo M, Prato O, Bagnasco G, Clavel B, Lepetz S, Davoudi H, Mashkour M, Stockhammer P, Krause J, Haak W, Morales A, Benecke N, Hofreiter M, Ludwig A, Graphodatsky A, Kiryushin KY, Baryshnikov G, Petrova E, Sablin M, Tishkin A, Ananyevskaya E, Logvin A, Logvin V, Kalieva S, Kukushkin I, Merz I, Merz V, Sakenov S, Shevnina I, Varfolomeyev V, Zaibert V, Arbuckle B, Reinhold S, Hansen S, Roslyakova N, Kosintsev P, Kuznetsov P, Anthony D, Kroonen GJ, Kristiansen K, Wincker P, Outram A, Orlando L. 2021 The origins and spread of domestic horses from the Western Eurasian steppes. Nature 598: 634-640. DOI: <https://doi.org/10.1038/s41586-021-04018-9>

Lyonnet B. (ed.). 2007 *Les cultures du Caucase (VII^e^-III^e^ millénaires avant notre ère). Leurs relations avec le Proche-Orient*. ERC, Paris: CNRS.

Lyonnet B., Akhundov T., Almemedov K. Bouquet L., Courcier A. Jellilov B., Huseynov F., Loute S., Makharadze Z., Reynard S. 2008 Late Chalcolithic kurgans in Transcaucasia. The cemetery of Soyuq Bulaq (Azerbaijan). Archäologische Mitteilungen aus Iran und Turan 40 (2), 115-130.

Marro C., Bakhshaliyev V., Le Bourdonnec F.-X., Orange M. 2018 A Multi-Directional Bridge? The Geo-Stratigraphic significance of Nakhchivan during the Late Chalcolithic (4500-3500 BCE). In: Giemsch L., Hansen S. (eds.) The Caucasus, bridge between the urban centres in Mesopotamia and the Pontic steppes in the 4th and 3rd millennium BC. The transfer of knowledge and technologies between East and West in the Bronze Age. Schriften des Archäologischen Museums Frankfurt (Regensburg 2021), 15-30.

Marro C, Bakhshaliyev V, Berthon R, Thomalsky J. 2019 New light on the Late Prehistory of the South Caucasus: Data from the recent excavation campaigns at Kültepe I in Nakhchivan, Azerbaijan (2012-2018). *Paléorient. Revue pluridisciplinaire de préhistoire et de protohistoire de l’Asie du Sud-Ouest et de l’Asie centrale* 45-1: 81-113.

Marro C. 2022. The view from the North, the emergence and spread of the Chaff-Faced Ware oikumenè as seen from the Caucasus (ca. 4600 – 3500 BCE). *Paléorient* 48-1, 112-130.

Meyer J-W, Vila E, Mashkour M, Casanova M, Vallet R (Eds.) (2019) The Iranian Plateau during the Bronze Age : Development of urbanisation, production and trade. MOM Éditions, Lyon.

Milwright M. 2010 *Introduction to Islamic archaeology*. Edinburgh University Press.

Muscarella OW. 2006 Iron Age. *Encyclopaedia Iranica*. XIII, 605–609

Nishiaki Y, Zeynalov A, Mansrov M, Akashi C, Arai S, Shimogama K, Guliyev F. 2019 The Mesolithic-Neolithic interface in the Southern Caucasus: 2016–2017 excavations at Damjili Cave, West Azerbaijan*. Archaeological Research in Asia* 19: 100140.

Olszewski D.I. 2012 The Zarzian in the context of the Epipaleolithic Middle East. *The International Journal of Humanities* 19(3): 1-20.

Peters J, von den Driesch A, Helmer D. 2005 10. The Upper Euphrates-Tigris Basin: cradle of agro-pastoralism? In *The First Steps of Animal Domestication. New Archaeological Approaches* (eds J-D Vigne, J Peters, D Helmer), pp. 96–124. Oxford: Oxbow Books.

Rante R, Fouache E, & Mirzaakhmedov D. 2016 Dynamics of human settlements ensuing from river transformation and changes in commercial behaviour: The birth of the “North-eastern Silk Road”. Journal of Archaeological Science: Reports 9: 437-447. <https://doi.org/10.1016/j.jasrep.2016.08.008>

Richter T, & Darabi H. (eds.). 2023 *The Epipalaeolithic and Neolithic in the Eastern Fertile Crescent: Revisiting the Hilly Flanks*. London and New York: Routledge.

Riehl S, Zeidi M, Conard NJ (2013) Emergence of Agriculture in the Foothills of the Zagros Mountains of Iran. Science, 341, 65–67. <https://doi.org/10.1126/science.1236743>

Rova E., Puturidze M., Makharadze Z. (2010). The Georgian-Italian Shida Kartli Archaeological Project: a report on the first two field seasons 2009 and 2010. *Rivista di Archaeologia*, 5-42.

Roustaei K, Mashkour M, and Tengberg M. 2015 Tappeh Sang-e Chakhmaq and the beginning of the Neolithic in north-east Iran. Antiquity 89(345): 573-595. <https://doi.org/10.15184/aqy.2015.26>

Roustaei K. 2016 An emerging picture of the Neolithic of Northeast Iran. *Iranica Antiqua* LI: 21-55. doi: 10.2143/IA.51.0.3117827.Sagona A. 2018. The Archaeology of the Caucasus. From Earliest Settlement to the Iron Age. Cambridge University Press, New York.

Sauer EW, Chologauri L, Gabunia A, Hopper K, Lawrence D, MacDonald E, Mashkour M, Mowat FA, Naskidashvili D, Pitskhelauri K, Priestman SMN, Shumilovskikh L, Simpson SJ, Tiliakou AI, Abe Y, Batt CM, Gagoshidze G, Gilmour B, Greenwood DP, Pickard C, Poole I, Radu V, Snape L, Snyder JR, Stoetzel E, Amiribeirami S, Astolfi M, Bailiff I, Beizaee Doost S, Bonato E, Clavel B, Debue K, Decruyenaere D, Diana A, Fathi H, Jajanidze R, Kamjan S, Kainaat T, Khazaeli R, Kranioti E, Lemoine M, Philip G, Shupe C, Shikaku R, Stern B, and Stetkiewicz S. 2020 *Dariali: The "Caspian Gates" in the Caucasus from Antiquity to the age of the Huns and the Middle ages: The joint Georgian-British Dariali gorge excavations & surveys of 2013-2016.* (Vol. 1). Oxbow Books.

Stöllner T, Gambashidze I, Al-Oumaoui I, Baldus T, Berthon R, Belošić A, Bode M, Boenke N, Broomand N, Bungardt J, Courcier A, Fehren-Schmitz L, Ghalichi A, Gogochuri G, Haak W, Kvavadze E, Jansen M, Löffler I, Mindiashvili G, Murvanidze B, Otkhvani N, Schapals F, Senczek S, Tshornaliani N, Vautrin A. 2023 Between Valleys and Mountains. The Dzedzvebi Plateau as an Intermediate Settlement Site of Chalcolithic and Early Bronze Age Communities in the Lesser Caucasus. *Archaeologia Austriaca* 107, 65-136.

Suska-Malawska M, Kot M, Gręzak A, Mętrak M, Khudjanazarov M, & Szymczak K. 2022 Potential impact of Holocene climate changes on camel breeding practices of Neolithic pastoralists in the Central Asian drylands: A preliminary assessment. The Holocene 32(11): 1132-1143. https://doi.org/10.1177/09596836221114289.

Taylor WTT, Pruvost M, Posth C, Rendu W, Krajcarz MT, Abdykanova A, Brancaleoni G, Spengler R, Hermes T, Schiavinato S, Hodgins G, Stahl R, Min J, Alisher kyzy S, Fedorowicz S, Orlando L, Douka K, Krivoshapkin A, Jeong C, Warinner C, Shnaider S (2021) Evidence for early dispersal of domestic sheep into Central Asia. Nature Human Behaviour, 5, 1169–1179. <https://doi.org/10.1038/s41562-021-01083-y>

Thornton CP. 2009 The Emergence of Complex Metallurgy on the Iranian Plateau: Escaping the Levantine Paradigm. *Journal of World Prehistory* 22: 301–327. https://doi.org/10.1007/s10963-009-9019-1

Vautrin A (2025) From summer pasture to mountain village: an archaeozoological approach to the establishment of agro-pastoral communities in the Caucasus mountains (VIth-IInd mill. BCE). PhD Thesis Thesis. Muséum National d’Histoire Naturelle, Paris.

Vautrin A., Bosch D., Balasse M., Bray F., Bruguier O., Fiorillo D., Le Meillour L., Bakhshaliyev V., Marro C., Mashkour. M and Berthon R. (Accepted and reviewed). Caprine Management during the Neolithic in the South Caucasus: Insights into Zootechnical Practices and Pastoral Mobility through Archaeological and Isotopic Studies (δ^18^O, δ^13^C, ^87^Sr/^86^Sr) at Kültepe I (Nakhchivan, Azerbaijan). *Paléorient*

Wordsworth PD. 2024 Moving in the Margins: Desert Travel and Power in Medieval Central Asia. Vol. 32, Leiden: Brill.

Zeder M. 2024 Out of the Shadows: Reestablishing the Eastern Fertile Crescent as a Center of Agricultural Origins: Part 1. *Journal of Archaeological Research* **33**, 1–56. (doi:10.1007/s10814-024-09195-5)
