# Supplementary material for: Bony labyrinth taxonomic identification and long-term evolution of livestock in the Southwest and Central Asia
Source: R Soc Open Sci. 2026 Oct 7;13(10):261493. doi: 10.1098/rsos.261493 (PMC13641910; doi:10.1098/rsos.261493)
Supplement: Supplementary file 1 [file rsos.261493.zip › rsos.261493/rsos261493_si_020.docx]

**SI_Text 3. Description of Study Sites.** The number before each site refers to the number reported in Figure 1 in the main text and SI Table 2. The coordinates of the sites are given next to the name of the site in the geographic coordinate system (WGS 84), with latitude listed first (north) and longitude second (east).

**Türkiye (Marjan Mashkour and Rémi Berthon)**

**1- Tilbeshar (Gaziantep)** (36.873888°, 37.558333°)

Tilbeshar was excavated during several seasons of excavation from 1999 by Christine Kepinski. The site is located 20 km northwest of Gaziantep in eastern Anatolia, a well-irrigated landscape, and near the Sajur River. The site was identified with Late Chalcolithic to Middle Bronze Age levels. In area D of the site, some pre-medieval and medieval levels were also found (Berthon & Mashkour, 2008).

The single sample from the site is MMTB01.

**Caucasus area**

The Caucasus is a mountainous and volcanic region located between the Black and Caspian Seas, characterised by varied reliefs and plains. This vast area is divided into two main zones: the North Caucasus, which lies on the northern slopes of the Greater Caucasus Mountain range and is currently part of the Russian Federation. The South Caucasus, where the archaeological remains of this study are from, is located in the southern part of the Greater Caucasus and is shared by the modern countries of Georgia, Armenia and Azerbaijan, as well as parts of Türkiye and Iran.

In this paper, five sites are located in this area and are dated from the Chalcolithic to the Medieval period.

The general framework of pastoralism in the South Caucasus begins in the Neolithic with the arrival of agro-pastoral societies who arrived with domestic plants and animals during the end of the 7th millennium BCE (see a review in Vautrin et al., accepted). Subsistence relies on a productive economy, while hunting is not that important. The main domestic species are the caprines, followed by cattle and pigs. The proportion of cattle/pigs depends on the sites. During the Chalcolithic, caprines are still in the majority, but the proportion of cattle increased, while pigs are almost absent from the faunal remains (Vautrin, 2025). During the Early Bronze Age the composition of the herd is more or less similar to that of the Chalcolithic (Vautrin, 2025). It is with the Middle Bronze Age, the Late Bronze Age and the transition to the Iron Age that changes are identified. The presence of domestic horses is attested as well as a change of status of pigs, which are represented in a larger proportion, sometimes equal to or greater than caprines and cattle (Vautrin, personal communication). During the Antiquity and Medieval periods (in Dariali), the sheep and goats dominated the assemblages, followed by cattle. Pigs are not identified (Mashkour et al., 2020).

**Georgia (Adeline Vautrin)**

**2- Aradetis Orgora** (42.046625°, 43.860500°)**, and 3- Doghlauri** (42.050861°, 43.855911°)

The mounds of Aradetis Orgora are located in Georgia, in the plain of the Kura Valley, at an altitude of 650 m asl. The site has been occupied for a long period, from the end of the IVth mill. BCE to the 6th c. CE. It was excavated by several Georgian expeditions and, between 2013 and 2016, by the Georgian-Italian Shida Kartli Project under the supervision of E. Rova and I. Gagoshidze. Three areas were excavated: Fields A and B belong to the Bronze Age and the Early Iron Age, while Field C corresponds to the Hellenistic palace. The Bronze Age and Early Iron Age occupations, where the material was sampled for this study, cover a range dated from 3000 to 700 BCE. Doghlauri is the name of a cemetery associated with the site, where hundreds of graves were excavated, belonging both to the Early Bronze and the Late Bronze Age. It was mainly excavated between 2012 and 2016 by a Georgian salvage expedition headed by Iulon Gagoshidze. In this study the material from Doghlauri only belongs to the Late Bronze Age period (Gagoshidze and Rova, 2016; Razzia et al., 2021; Gagoshidze et al., 2025).

For Aradetis Orgora, samples AO328, AO663, AO697, AO1287, AO1549, AO1569, AO1716, AO2467, AO3048, AO3092, AO3133, AO3458, AO3970, AO4361, AO4630, AO4691 and AO5020 were included in this study. For Doghlauri, samples DL28, DL68 and DL141 were studied.

**4- Natsargora** (42.070514°, 43.715300°)

The mound of Natsargora is located close to the plain of the Kura valley in Georgia, at an altitude of 760 m asl. The site was occupied throughout the Bronze Age, with the main occupations during the Early Bronze Age and the Late Bronze/Early Iron Age. More than five hundred graves were excavated, 26 of which belong to the Early Bronze Age. The mound is 20-25 m high, oval in shape, and measures 90×50 m. The site was first excavated between 1984 and 1992 by the Georgian National Museum under the supervision of A. Ramishvili. Later, in the early 2010s, it was restudied by the Georgian-Italian Shida Kartli Archaeological Project (Rova et al., 2010; Rova et al., 2017). The material studied in this project mainly belongs to the Early Bronze Age and the Late Bronze Age.

Samples NTS866, NTS1292, NTS1463, NTS1465, NTS2971, NTS3696, NTS4344 and NTS4893 were included in this study.

**5- Orchosani** (41.593076°, 42.825589°)

The site is located in Georgia at the border with Türkiye at an altitude of 1,225 m asl. It was first excavated between 2002 and 2005 by A. Orjonikidze and M. Baramidze during the period of the construction of the Baku-Tbilisi-Ceyhan (BTC) pipeline and was excavated again in 2014, 2015 and 2016 by I. Gambashidze and D. Mindroashvili from the Georgian National Museum within the frame of the Pipeline Expansion Project (SCPX). The material of this study comes from the latest excavations. The site was occupied during the Late Chalcolithic, Early Bronze Age, Late Classical and Early Middle Ages. For the Chalcolithic period, 109 pits, a building and 4 ovens were excavated. The samples from this study belong to the Chalcolithic period, attributed to the first half of the fourth millennium BCE (Gambashidze, 2021).

The sample Orcho485 was included in this study.

**6- Dzedzvebi** (44.388575°, 41.371934°)

The site is located in Georgia on the Dzedzvebi Plateau at an altitude of 730 m asl on a basaltic rocky spur where two rivers, the Mashavera and the Ukangorula, flow on either side. To the west, the site is linked to the Trialeti and Javakheti plateaus and the volcanic plateau of Mount Chikiani, where there are obsidian deposits, and the Armenian high plateau. To the northeast, the site links up with the Kura Valley, following the course of the Mashavera. The site is located close to several mineral deposits, in particular copper and gold linked to the Madneuli-Sakdrisi deposits. Since 2007, it has been the subject of archaeological excavations by the Deutsches Bergbau-Museum / Ruhr Universität Bochum and the Georgian National Museum, directed by Thomas Stöllner and Irina Gambashidze. The site shows evidence of long-period occupation with major establishments dated from the Chalcolithic, Bronze Age (early and middle) and Iron Age. The Chalcolithic settlement appears to be restricted to the south of the plateau. Two phases of occupations are documented on the rocky spur; the first one is dated to the Early Chalcolithic (4900 - 4700 BCE), and the second is attributed to the Late Chalcolithic (4400 - 4000 BCE). The occupations belong to the Sioni cultural complex, based on the study of pottery. A total of 17 pits with Sioni pottery have been documented, and some of them have been disturbed by later Early Bronze Age and Iron Age occupations. The Kura-Araxes occupations (dated to the 3600/3500 - 2700 BCE range) are larger and occupy area IV, the slopes of Dzedzvebi II and the northern part of Dzedzvebi III. These occupations are characterised by a settlement including areas for crafting activities and burial groups (Stöllner *et al*., 2023).

The samples Dz.av.1, Dz.av.1373, Dz.av.1837, Dz.av.1843, and Dz.av.1847 were included in this study.

**7- Dariali/Tamara’s Fort (Caucasian Gates)** (42.738050°, 44.625633°)

The fort of Dariali is located in the Greater Caucasus at an altitude of 1,350 m asl. The site was investigated within the ERC project “Persia and its Neighbours”, directed by E. Sauer, K. Pitskhelauri and others. The earliest evidence of human occupation on the site dates back to between the 6th and 4th c. BCE. The fort was constructed in the late 4th c. CE (or early 5th c. CE at the latest). The site was then occupied intensively until the late 10th/early 11th c. CE and reoccupied between the late 13th and early 15th c. CE. Except for the 20th c., when some military structures were positioned within the fort, there is no traceable post-medieval occupation (Sauer et al., 2020). The very large number of faunal remains was studied by Mashkour et al. (2020).

The sample MMCG13 (Trench Q, context Q.03), included in this study, belongs to the phase dated to the late 7th to 9th c. CE.

**Azerbaijan (Rémi Berthon)**

**8- Uçan Ağıl (Nakhchivan, Azerbaijan)** (39.30005°, 45.547263°)

Uçan Ağıl is a flat site located at 1200 m asl in the Sirab piedmonts (Nakhchivan Autonomous Republic, Republic of Azerbaijan). The site has been excavated since 2015 in the frame of the "Mission archéologique du Bassin de l'Araxe" under the supervision of Catherine Marro (CNRS) and Veli Bakhshaliyev (Azerbaijan National Academy of Sciences). Excavated areas mostly yielded occupation remains belonging to the 4850-4350 BCE time span, corresponding to the Chalcolithic period in the region, but the site was also occupied during the 3rd millennium BCE (Early Bronze Age – Kura-Araxes culture). Judging by its stratigraphic sequence and material assemblage, Uçan Ağıl was a mobile camp used by pastoral groups (Marro, 2022).
The sample UA01220 was included in this study. It comes from the Early Chalcolithic occupation.

**Iraqi Kurdistan – Sulaymaniyah** **(Hitomi Hongo & Marjan Mashkour)**

**9- Jarmo** (36.533333°, 44.833333°)

Jarmo (also known as Charmo in Kurdish) is a prominent Neolithic archaeological site located in the Sulaymaniyah district, situated at approximately 725 metres above sea level in the foothills of the Zagros Mountains, within the territory of Iraqi Kurdistan. Initially identified by the Iraqi Directorate of Antiquities in 1940, the site was later excavated by Robert and Linda Braidwood under the auspices of the Oriental Institute of the University of Chicago (Braidwood et al., 1983). This is a key site for the understanding of early plants and animals in the Zagros. Since 2016, a University of Tsukuba research team directed by Akira Tsuneki (2024) has been conducting renewed investigations within a joint Iraqi-Japanese archaeological project. According to multiple recent ^14^C dates (Itahashi et al., 2024), PPN and PN layers were uncovered and cover 1000 years of occupation at the site. The faunal remains from the Braidwood and Braidwood’s excavations were studied by Stamplfi (1983). The remains of the Japanese excavations were studied mainly by Hitomi Hongo and later with Marjan Mashkour (Hongo and Mashkour, unpublished report). They are dominated by wild and domestic ungulates (caprini, cattle, pigs, red deer, gazelles and dogs).

Samples HHJM15-a, HHJM15-c, HHJM15-d, HHJM16-a, HHJM16-b, HHJM17-b, HHJM17-c, HHJM20-a, HHJM20-b, HHJM20-d, HHJM21, HHJM8-a, HHJM8-b, and HHJM8-c were included in this study and belong to the Pre-Pottery Neolithic period.

**Iranian Plateau (Hossein Davoudi & Marjan Mashkour)**

**10- Dava Göz (West Azerbaijan, Iran)** (38.490277°, 45.032222°)

Tepe Dava Göz settlement, located near Khoy in northwestern Iran (1146 m asl.), is a small archaeological mound measuring approximately 1 hectare. Excavations conducted by Akbar Abedi in 2012 revealed the stratigraphy of the site, which spans the Late Neolithic to Transitional Chalcolithic and Chalcolithic periods. Notably, the Hajji Firuz materials indicate that Dava Göz functioned as a seasonal campsite, while Pisdeli remains were found primarily in a thicker cultural layer. The site is located between the Southern Caucasus and Lake Urmia in the lowlands and is a suitable target for mobile agro-pastoral human communities for winter pastureland. The site provides valuable insights into the development of communities in the Lake Urmia basin from the 6th to 4th millennium BC and their interactions with contemporary cultures in the Caucasus and Eastern Anatolia (Abedi *et al*., 2015; Abedi, 2017).

The faunal remains from Tepe Dava Göz were analysed at the Bioarchaeology Laboratory of the University of Tehran. Across all periods, domesticated sheep and goats were the most exploited animals at the site. Cattle remains were also identified at Dava Göz, and the presence of aurochs during the Late Neolithic period is considered possible. In addition, remains of wild animals, including wild horse, hemione, wild sheep and goat, deer, and gazelle, were recovered. Canid remains, specifically dog and fox, were also identified (Davoudi & Mashkour, 2017; Davoudi *et al*., 2019a).

Samples MMDG01, MMDG03, MMDG04, and MMDG05 were included in this study and belong to the Late Neolithic and Early and Late Chalcolithic periods.

**11- Hasanlu (West Azerbaijan, Iran)** (37.004346°, 45.458833°)

Tepe Hasanlu is located in the Solduz valley on the southern shore of Lake Urmia at 1043 m asl in West Azerbaijan province. Due to long-term occupation and defined stratigraphy, Tepe Hasanlu is one of the key sites of northwestern Iran. Robert H. Dyson Jr. directed 10 seasons of excavations from 1956 to 1977 [(Dyson & Muscarella, 1989; Muscarella,](https://paperpile.com/c/GyUccA/Xaih+fPi6) 2006), which revealed its occupation during 10 different cultural periods from the Late Neolithic (period X) to the Ilkhanid dynasty (period I). The most highly represented periods are the Late Bronze (period V) and Iron Age (periods IV-III) [(Danti, 2013)](https://paperpile.com/c/GyUccA/LmAq). Periods IIIc and IIIb are attributed to Iron Age III (Urartian period) [(Kroll, 2013)](https://paperpile.com/c/GyUccA/jPvw). The fauna assemblage was studied in the Osteology Department of the National Museum of Iran by H. Davoudi [(Davoudi, 2017)](https://paperpile.com/c/GyUccA/9smm). The bone assemblages of animals discovered in Hasanlu were divided into three main classes: mammals (Bovidae, Caprinae, Cervidae, Suidae, Equidae, Camelidae, Canidae, Mustelidae, Leporidae and Muridae), birds (Pelecanidae, Ardeidae, Ciconiidae, Anatidae, Phasianidae, Columbidae and Corvidae), and reptiles (Testudinidae) [(Davoudi *et al*., 2018; Davoudi & Mashkour, 2019)](https://paperpile.com/c/GyUccA/7hlW+SiyG).

Samples MMTH04, MMTH05, MMTH08, MMTH09, MMTH10, MMTH13, and MMTH22 were included in this study and belong to the Early Bronze Age, Iron Age, and Middle Islamic period.

**12- Koulian Cave (Kermanshah, Iran)** (34.73119°, 46.65802°)

Koulian Cave (also Kulian) is located near the city of Rawansar, about 52 km northwest of Kermanshah, in West Iran. The site contains Middle Palaeolithic, Upper Palaeolithic, Chalcolithic and later archaeological deposits (Biglari & Taheri, 2000). The cave is about 20 m long and consists of two chambers. Animal bone accumulation in the inner chamber most likely originated from carnivore activity and natural death. The faunal remains recovered from pits dug by pothunters are predominantly associated with Palaeolithic and Chalcolithic archaeological materials, and many of them exhibit cut marks, burning, and other evidence of anthropic modification. The collection includes identified bones of wild herbivores and carnivores (Mashkour, 2001). One of the equid bones was dated to the time of the reign of Sasanian king Shapur II (309–379 CE) (Fages *et al*. 2019).

The sample MMKU01 was included in this study.

**13- Zilou Cave (Kermanshah, Iran)** (34.564890°, 47.082000°)

Zilou Cave is a high-altitude karstic cave located on the Paraw Mountain in the central Zagros Mountains of western Iran. Situated at approximately 2,500 m above sea level on Alivar Mountain, the cave overlooks the Shirali Karst Valley and lies within the High Zagros (or “Crushed Zone”) in the northwest of Kermanshah City, Kermanshah Province. It is one of the few known karstic caves in the region that contains exceptionally rich faunal remains. The cave contains two deep vertical shafts that have functioned as natural traps for local fauna over many millennia. The bottoms of both shafts are densely covered with animal remains, most of which are well preserved. High levels of precipitation (primarily snowfall), combined with the cave’s difficult access and vertical morphology, have contributed to excellent bone preservation and minimal post-depositional disturbance (Taheri *et al*., 2007).

The faunal assemblage is dominated by wild goats and sheep, while carnivores are represented by species such as bears, wolves, and foxes. The cave’s elevation, which exceeds the estimated Upper Pleistocene snow line, suggests that it was largely inaccessible to fauna during the Last Glacial Period, except possibly during short warmer interstadial phases. It is therefore reasonable to propose that most of the faunal remains from Zilou Cave date to the last interglacial period (ca. 125,000–130,000 years ago) or the Holocene. During much of the Late Pleistocene, the cave was likely buried beneath permanent or seasonal snow, with limited accessibility continuing into the Holocene. However, absolute dating of wild goat remains has produced a date of approximately 30,000 BP, indicating at least some Late Pleistocene faunal activity at the site (Mashkour & Mohaseb, 2012).

The sample MMZC01 was included in this study.

**14- Kulluk [Chehrābād Salt Mine complex] (Zanjan, Iran)** (36.879100°, 47.871686°)

The Douzlākh (Chehrābād) Salt Mine is located in the Talkherud Basin, near Chehrābād village in Zanjan Province, northwest Iran. To date, eight so-called “Salt Men” have been identified at the mine. Several of these individuals retain exceptionally preserved keratinous tissues, including skin and hair, as well as both endo- and exoparasites. Radiocarbon dating places them within the Achaemenid (550–330 BCE) and Sasanian (224–651 CE) periods. The mine and its associated settlements have been excavated by a joint Iranian–German archaeological expedition directed by Abolfazl Aali and Thomas Stöllner. Archaeological evidence indicates that the mine was in use from the 6th century BCE until the 20th century CE. In addition to the mine itself, several other sites within the Talkherud Basin—such as Tappeh Haj Hatam, Kūzehchi, Reza-ābād South, Zarin Tappeh, Kulluk, and Tappeh Sheikh Attar—have also been investigated. Meanwhile, Kuzehchi from the Achaemenid period and Zarrin Tappeh from the Sassanid era have been excavated. Given their close geographical proximity, it is likely that some of these settlements were closely connected to salt-mining activities. Tepe Kulluk is one such site, excavated in 2017 and dated to the Late Islamic period, spanning the Safavid to Qajar dynasties (15th–19th centuries CE) (Aali *et al*., 2012; Stöllner *et al*., 2020; Stöllner *et al*., 2024).

Archaeozoological studies of the Chehrābād Salt Mine and its associated sites were conducted at the Bioarchaeology Laboratory of the University of Tehran and UMR 7209 BioArch Laboratory of the National Museum of Natural History in Paris by Marjan Mashkour and colleagues. The faunal assemblage from Chehrābād comprises two main categories: anthropogenic remains, primarily representing food waste and activities related to mining, and remains associated with the mine’s natural environment. Exceptional preservation conditions created by the presence of salt enabled the recovery of a wide range of organic materials, including feathers, hair, wool, fibres, skin, leather, faeces, muscles, cartilage, and bones. Remains of sheep, goat, cattle, pig, and chicken were identified in Achaemenid and Sasanian contexts within the mine. At the Kulluk site, remains of domesticated sheep, goats, cattle, equids, chickens, birds, and rodents were documented. Notably, the presence of horse and donkey remains at Kulluk is of particular interest and may indicate their use in transport or labour related to mining activities (Mashkour *et al*., 2020; Stöllner *et al*., 2024).

The sample MMCHD1 was included in this study and belongs to the Late Islamic period.

**15- Abdul Hosein (Lorestan, Iran)** (34.052500°, 48.131388°)

Tepe Abdul Hosein is a highland site located at 1860 m asl in the Province of Luristan between Khorramabad and Malayer in the Zagros Mountains of Iran. The mound was excavated by Judith Pullar in 1978, and a unique Pre-Pottery occupation during the early Neolithic was discovered. The lower levels consist of ashy deposits, and the upper levels contained more substantial mud-brick architecture (Pullar, 1990). Recent genetic studies on the human remains of the site also underline the great potential of the site to provide information on the Neolithic people of the Zagros and their spread to the East (Broushaki *et al*., 2016; Daly *et al*., 2018).

The faunal remains from the site are stored in the Osteology Department of the National Museum of Iran and are indicative of a diversified subsistence economy, with wild and domestic goats playing a prominent role. In order to distinguish between wild and domestic goat skeletal elements, several interdisciplinary methods are used. The criteria include horn core morphology, univariate metric analyses on different skeletal parts, and LSI analyses on the postcranial bones in comparison to both modern and Palaeolithic/Epipalaeolithic reference material. Kill-off patterns were also employed in analysing the assemblage. The sample described here dates to the late 9th / early 8th millennium cal BCE, corresponding to the Early Neolithic, and the various resources exploited include wild goats and sheep, boars, aurochs and red deer. Goat remains appear in the early levels, and domesticated sheep in the later levels of settlement when a small village was present (Mashkour *et al*., 2018; Mashkour *et al.*, 2021).

Samples MMTAH01, MMTAH03, MMTAH04, MMTAH05, MMTAH06, MMTAH07, MMTAH08a, MMTAH08b, MMTAH10, MMTAH11a-L, MMTAH11a-R, MMTAH11b-L, MMTAH11b-R, MMTAH12, MMTAH13, MMTAH14, MMTAH16, MMTAH17, MMTAH18a, MMTAH18b, MMTAH18c, MMTAH19, MMTAH20, MMTAH21, MMTAH22, MMTAH23, MMTAH24, MMTAH26, MMTAH27a, MMTAH27b, MMTAH28a-L, MMTAH28a-R, MMTAH28b, MMTAH28c, MMTAH29, MMTAH30, and MMTAH31 were included in this study and belong to the Pre-Pottery Neolithic and Early Chalcolithic periods.

**16- Forudgah (Lorestan, Iran)** (33.4451389°, 48.237861°)

The archaeological site known as Tepe Forudgah is located on a flat plain within the Khorramabad Airport area in Lorestan Province. The mound rises approximately 5 metres above the surrounding terrain and has a diameter of about 80 metres. Due to its location, the site is under significant threat from developmental activities; consequently, a salvage excavation was conducted by Ali Sajadi in 2014. The excavation revealed evidence of several cultural periods at Tepe Forudgah. The uppermost phase dates to the post-Achaemenid and early Parthian periods. The second phase contains remains attributed to Iron Age II or III, followed by a third phase associated with the Bronze Age. The lowest and fourth phase is dated to the Late Chalcolithic period (Sajadi, 2015; Tavassoli *et al*., 2016). Faunal remains from the site were analysed at the Bioarchaeology Laboratory of the University of Tehran. The assemblage consists predominantly of domesticated herbivores. Notably, cattle outnumber sheep and goats in the collection. Several bones of horses and donkeys were also identified, mainly dating to the historical periods. In addition, remains of hunted wild species—including red deer, gazelle, wild boar, and hemione—were recovered (Mashkour & Fathi, 2015).

The sample MMTF01 was included in this study and belongs to the Iron Age.

**17- Susa Complex (Khuzestan, Iran)** (32.189166°, 48.257777°)

Susa (Persian *Shush*; ancient *Šušen / Šušan*) is a UNESCO World Heritage Site (<https://whc.unesco.org/en/list/1455>) located in Khuzestan Province, southwestern Iran, near the modern city of Shush. The site lies on the fertile Susiana Plain between the Karkheh and Dez rivers, with the Shavur River forming its eastern boundary. Susa is one of the few archaeological sites in the region to have been the subject of continuous investigation since the first half of the nineteenth century (Carter, 1992; Ahmadzadeh et al., 2021; Soraghi and Zeynivand, 2022). These investigations revealed a complex, multi-layered settlement sequence comprising up to 27 distinct occupational phases, spanning from approximately the sixth millennium BCE to the thirteenth century CE (de Mecquenem, 1934; Ghirshman, 1963; Perrot, 1978, 2010; Gasche, 2009).

During the last years 3 animal bone assemblages from Susa were found in the French Institution. One small assemblage is deposited at the Institute of Human Palaeontology of the MNHN. One assemblage, initially studied by Thérèse Poulain in the 70s, was transferred to the UMR 7209 BioArch Laboratory and restudied by Marjan Mashkour, Homa Fathi and Romain Perronet but not yet published in 2024. Another assemblage was recently found in the Louvre Museum and sent on loan to the MNHN to be studied by M. Mashkour. The bones are labelled "SH 093 227” and "SH 093 228". These materials originate from Roman Ghirshman's 1962-1963 excavations. While Ghirshman’s work at Susa extended over two decades, the 1962–1963 seasons formed part of a sustained effort to reach the site’s occupational levels through the clearance of extensive areas. During this period, Ghirshman focused on Chantier A (Site A) in the Ville Royale. By the 1960s, his team had identified fifteen distinct settlement levels, establishing a definitive ceramic stratigraphy for the region. These layers belonged to the Middle and Neo-Elamite dynasties, dating from the middle of the 2nd millennium BC to the 8th century BC (Ghirshman, 1963).

Between 2023 and 2024 the study of these materials was performed by several trainees within academic works (e.g., Martin, 2023) under the supervision of M. Mashkour. The faunal assemblage is dominated by domestic animals, primarily sheep, goats, and cattle. Equids and camels were also present at the site. Wild species are represented in small numbers and include gazelle, deer, and boar. Canids and other carnivores are rare. Several human bone fragments were also recovered within the assemblage.

The petrous sample Susa01 was included in this study and belongs to the Middle Elamite Dynasty (mid-second mill. BC).

**18- Soha Chay (Zanjan, Iran)** (36.312750°, 48.363194°)

Soha Chay Tepe (also Soha Chai), located in the Sojasrud Valley of western Iran, was excavated in 2006 by Abolfazl Aali and reveals a single-period settlement from the Early Middle Chalcolithic, specifically the late 5th millennium BC, linked to the Dalma cultural tradition (Rahimi & Eslami, 2018). The site covers less than one hectare and has a cultural deposit of 1.20 metres. The distinctive Dalma ceramic assemblage is prevalent in the northern and central Zagros Mountains, indicating a technically and stylistically homogeneous phenomenon across the region. Dalma pottery at Soha Chay Tepe should be understood as a persistent ceramic tradition spanning several Chalcolithic phases, with the site dating to the onset of the Late Chalcolithic 2 (Early Late Chalcolithic 2, ca. 4200-4000 BC) under Renette’s revised chronology (Renette and Mohammadi, 2020; Renette, 2022). Recent studies of the faunal and botanical remains at Soha Chay Tepe show a predominance of small ruminants, particularly sheep and goats, over gazelles, though interdisciplinary research on Dalma sites remains limited (Mashkour *et al*., 2015; Davoudi *et al*., 2019a).

Samples MMSCH01 and MMSCH04 were included in this study and belong to the Middle Chalcolithic period.

**19- Qela Gap (Lorestan, Iran)** (33.528790°, 49.344010°)

Tepe Qela Gap (also known as Ghala Gap) is located in the northwest of the Azna Plain, Lorestan Province, Iran, at an elevation of 1970 m asl., stands 25 m above the surrounding fields and covers an area of approximately 1.5 ha. This important site was excavated in 2009 by M. Abdolahi and A. Sardari, aiming to establish the chronological sequence for the Azna Plain (eastern part of the Central Zagros Mountains), which had been scarcely studied archaeologically until now. Three trenches were excavated, which revealed a noticeable sequence of occupations from the Late Neolithic to Parthian periods [(Abdollahi *et al*., 2014; Abdollahi & Sardari, 2013)](https://paperpile.com/c/GyUccA/2TVM+Amh4). Considering the ecological diversity surrounding the plain, Tepe Qela Gap seems to have been an ideal place for the settlement of prehistoric permanent villages but also for nomadic and semi-nomadic people. This feature was one of the incentives to study the large faunal assemblage (approximately 6500 specimens) of Tepe Qela Gap. The remains were studied by S. Amiri at the Bioarchaeology Laboratory of the University of Tehran. The faunal spectra indicate that the majority of the subsistence economy of the site relied on the exploitation of domesticated sheep/goats and cattle. The wild species, although not abundant, were also part of the subsistence economy. Remains of wild sheep, red deer or Persian fallow deer, wild boar, gazelle and hemione have been found and indicate that different ecosystems were exploited [(Amiri *et al*., 2019)](https://paperpile.com/c/GyUccA/63zZ).

The sample MMGG01 was included in this study and belongs to the Middle Bronze Age.

**20- Ālou (Qazvin, Iran)** (35.989577°, 49.526737°)

Ālou is located near the village of Radekan in Takestan County, Qazvin Province, and was excavated in the summer of 2016 under the direction of Hossein Davoudi. The site is an open-air settlement covering approximately 1 hectare and, unlike Kholāh Kouh, lacks a visible mound. Based on the absolute radiocarbon dating and pottery typology and the presence of administrative and technological devices characteristic of the period, the site has been dated to the Late Chalcolithic (ca. 3700–3400 BC), a key phase for understanding the origins of socioeconomic developments on the Central Plateau of Iran. Zooarchaeological evidence indicates that the primary exploited animals were domesticated caprines and cattle, while wild sheep and goats, boar, gazelle, and hemione were also hunted. Remains of dog and fox were also identified (Davoudi *et al*., 2019c; Moghimi, 2018; Moghimi & Davoudi, 2020).

The sample MMAU01 was included in this study and belongs to the Late Chalcolithic period.

**21- Kholah Kouh (Qazvin, Iran)** (36.060512°, 49.706981°)

Tepe Kholāh Kouh is located near the city of Takestan in Qazvin Province. A salvage excavation at the site was carried out in the summer of 2014 under the direction of Hossein Davoudi. The mound covers an area of approximately 3.5 hectares. Based on the absolute dating and pottery typology, the site has been dated to the transitional phase between the Neolithic and Chalcolithic periods (ca. 5200–4600 BC). Evidence recovered from approximately 4 m of cultural deposits includes residential structures, human burials, pottery, animal bones, spindle whorls, copper objects, and other artefacts. Zooarchaeological analysis indicates that the primary exploited animals were domesticated goats, sheep, and cattle, while wild caprines, gazelles, and hemiones were also hunted (Davoudi *et al*., 2016; Davoudi *et al*., 2021; Moghimi, 2018).

The sample MMKHK01 was included in this study and belongs to the Transitional Chalcolithic period.

**22- Sagzabad (Qazvin, Iran)** (35.816208°, 49.957131°)

Tepe Sagzabad (Qareh Tappeh), together with Tepe Qabrestan and Tepe Zagheh, forms a group of three closely related sites on the Qazvin Plain, located in the central district of Buin Zahra, approximately 60 km south of the modern city of Qazvin and about 140 km west of Tehran, in Qazvin Province. Tepe Zagheh lies 2 km east of Sagzabad, while Tepe Qabrestan is situated 300 m west of it. These sites were excavated in the 1970s under the direction of E. O. Negahban (Negahban, 1976). Multiple archaeological campaigns have demonstrated that Tepe Sagzabad is one of the few large mounds in the north-central Iranian Plateau that preserves evidence of continuous occupation throughout the Bronze and Iron Ages. As such, it represents a key site for investigating the transition from the Bronze Age to the Iron Age (Negahban, 1974; Dehpahlavan & Alinezhad, 2022). More recent excavations have also identified Late Chalcolithic occupation at Sagzabad (Azizi & Niknami, 2013). The extensive faunal assemblage, comprising more than 10,000 identified bone specimens, highlights the dominant role of domestic herbivores—particularly ovicaprines and cattle—followed by a significant contribution from domestic equids. Wild species, including sheep, goats, onagers, boars, gazelles, and deer, were also exploited (Mashkour *et al*., 1999; Mashkour, 2001).

The sample MMSZ01 was included in this study and belongs to the Iron Age.

**23- Zāgheh Ābyek (Qazvin, Iran)** (36.060512°, 49.706981°)

Tepe Zāgheh Ābyek is located in Zāgheh village, Ābyek County, Qazvin Province. A test at the site was conducted in the winter of 2018 under the direction of Hossein Davoudi. Zāgheh Ābyek is a large mound (tappeh) covering approximately 14 hectares. The site was occupied during the transitional phase from the Neolithic to the Chalcolithic period (ca. 5200–4600 BC) as well as the Early Chalcolithic period (ca. 4300–4000 BC). An overlap between Transitional and Early Chalcolithic deposits has been identified at only two sites on the Central Plateau of Iran; therefore, Zāgheh Ābyek holds significant potential for refining the chronology and duration of the transition between these two periods. The main exploited animals were domesticated caprines and cattle, while wild sheep and goats, boars, gazelles, and hemiones were also hunted (Moghimi, 2018; Davoudi *et al*., 2019b).

Samples MMZA01a, MMZA01b, and MMZA01c were included in this study and belong to the Early Chalcolithic period.

**24- Toll-e Sohzu (Bushehr, Iran)** (29.572470°, 50.882890°)

Toll-e Sohzu is located in the Shabankarah District, Khalifeh Village, Dashtistan County, Bushehr Province. The mound covers an area of approximately two hectares and rises about 4 m above the surrounding landscape. Toll-e Sohzu is one of the few sites in Bushehr Province, on the northern coast of the Persian Gulf, that contains prehistoric cultural layers. Surface materials indicate occupation during at least the fifth and fourth millennia BCE. To investigate the cultural sequence and gain a better understanding of the settlement structure, Trench A (4×4 m) was excavated at the top of the mound. Excavation reached virgin soil at a depth of 5.60 m below the surface. The results revealed evidence of the Middle Bakun culture (second half of the fifth millennium BCE) and the Lapui culture (ca. 3950–3400 BCE). Archaeological remains include mud-brick and pisé architectural features, oven remains, chipped stone tools, pottery vessels, copper tools and implements, animal bones, and charcoal fragments. Given the region’s hot and arid climate, Tol-e Sozu was likely used as a temporary or seasonal settlement, probably occupied during the fall and winter months (Azizi, 2022). Archaeozoological analyses conducted at the Bioarchaeology Laboratory of the University of Tehran identified a diverse faunal assemblage. Domestic species include goats, sheep, and cattle, while hunted animals comprise wild goats, deer, boar, and various fish species (Azizi *et al*., 2020).

The sample MMTOS02 was included in this study and belongs to the Late Chalcolithic period.

**25- Deh Dumen Graveyard (Kohgiluyeh and Boyer-Ahmad, Iran)** (31.15526°, 51.10758°)

The Deh Dumen Graveyard (also referred to as Deh Paeen Graveyard) is located in southwestern Iran, within the Khersan River valley. The site is particularly significant for its role as a cultural and commercial nexus linking the eastern and western regions of the Iranian Plateau during the Bronze Age. Administratively, it lies about 100 m southwest of Deh Dumen village, in the Pataveh District of Dena County, approximately 70 km northwest of Yasuj, in Kohgiluyeh and Boyer-Ahmad Province (Naseri & Khanipour, 2025). The site was first identified in 2008 during a rescue survey conducted prior to the construction of Khersan Dam 3 (Ghasemi & Watson, 2014). Subsequent rescue excavations over four seasons, directed by Reza Naseri in 2013, 2016, 2019 and 2021, resulted in the discovery of 42 graves. The burial assemblages exhibit stylistic affinities with several regional cultural traditions, including Luristan (Bani Surmeh), Susa (Susa D), and eastern Iranian centres such as Shahdad and Jiroft (Naseri & Khanipour, 2025; Oudbashi *et al*., 2021). The cemetery was in use for several centuries and can be divided into three main occupational phases. The majority of stone-built graves and jar burials date to the second half of the third millennium BCE, corresponding to the Early and Middle Bronze Age. A later phase of burial activity extends into the second millennium BCE, spanning the Late Bronze Age to the Early Iron Age. It should be noted that several graves provide evidence dating to the first half of the first millennium BCE. These contexts require absolute dating and may be considered representative of a third phase of burials within the cemetery (Naseri & Khanipour, 2025; Sołtysiak & Naseri, 2017).

Notably, some graves contain partial animal skeletons. Archaeozoological analysis of these remains was carried out at the Bioarchaeology Laboratory of the University of Tehran. Preliminary results indicate that sheep and goats are the most frequently represented species in the burial contexts. In addition, remains of cattle, birds, and boar have been identified in a limited number of graves (Davoudi *et al*., 2026).

Samples MMDD10, MMDD11, MMDD12, MMDD13, and MMDHD2 were included in this study and belong to the Bronze Age.

**26- Sialk (Kashan, Iran)** (33.968888°, 51.404722°)

Tepe Sialk is one of the largest and most intensively studied prehistoric sites on the Iranian Plateau and holds a key position in discussions of early cultural development in central Iran. Situated within the modern city of Kashan, in Isfahan Province, the site consists of two distinct mounds with different occupational histories. The first excavations at Tepe Sialk were carried out between 1933 and 1937 by a French archaeological mission led by Roman Ghirshman (Ghirshman, 1939). Following several decades without major fieldwork, new investigations were initiated in 2001 and continued until 2006 under the Sialk Reconsideration Project, organised by the Iranian Center for Archaeological Research and directed by Sadegh Malek Shahmirzadi (Malek Shahmirzadi, 2003, 2004, 2006). More recently, since 2008, renewed excavations on the northern mound have been undertaken by an Iranian team under the supervision of Hassan Fazeli Nashli (Fazeli *et al*. 2013). The northern mound was primarily settled during the Late Neolithic period, in the sixth millennium BCE (Malek Shahmirzadi, 2006; Fazeli Nashli *et al*., 2013), whereas the southern mound experienced a longer and more complex sequence of occupation. This southern mound was inhabited continuously throughout the Chalcolithic period, beginning in the mid-fifth millennium BCE and extending into the early third millennium BCE (Early Bronze Age). After a period of abandonment, the site was briefly reoccupied during the Late Bronze Age and Early Iron Age (Malek Shahmirzadi, 2003; Nokandeh, 2010).

Archaeozoological research associated with the first series of the Sialk Reconsideration Project was conducted by Marjan Mashkour. Analysis of faunal remains from the Chalcolithic levels of the southern mound demonstrates a mixed economy that included domesticated caprines and cattle, alongside a variety of wild species such as equids, gazelle, boar, rabbit, birds, and turtle. Located in a semi-arid zone on the margins of the central desert of Iran, the Sialk region offered suitable habitats for animals such as the Persian onager and gazelle. These species appear to have been hunted occasionally by the inhabitants of Tepe Sialk, supplementing a primarily pastoral subsistence strategy (Mashkour, 2002).

The sample MMSN01 was included in this study and belongs to the Late Neolithic period.

**27- Chahar Bagh Street (Isfahan, Iran)** (32.656972°, 51.669138°)

Chahar Bagh Street, also known as Abbasi Chaharbagh Street, is located in the southwestern part of Isfahan. It was planned in 1596–1597 CE and constructed in 1597–1598 CE, coinciding with the transfer of Iran’s capital to Isfahan during the reign of Shah Abbas I (1588–1629 CE). Prior to the construction of the street, the area consisted of four gardens established by Shah Abbas I. In the Islamic period, this part of the city functioned as a suburban zone due to its proximity to the Zayandeh Rud River. The construction of Chaharbagh Street continued until 1602–1603 CE on both sides of the river, and by 1607–1608 CE the associated streams, gardens, ponds, and fountains were completed. In the same year, construction of the Allah Verdi Khan Bridge began; upon its completion, Chaharbagh became a continuous axial boulevard connecting the northern and southern parts of Isfahan. The street extends approximately 6 km in length. In 2020, archaeological salvage excavations were conducted in the central section of Chaharbagh Street, opposite Amadgah Street and Enghelab Square, under the supervision of Mohsen Javeri. These excavations were necessitated by the construction of a subway tunnel passing beneath Chaharbagh Street at a depth of approximately 14 metres. Six trenches, measuring between 7 and 15 metres in length, were excavated along the central axis of the street. The excavations yielded cultural materials primarily from the Safavid period, including brick pavements dating to the Qajar period, which had been added to the original Safavid structure. Evidence from the Early Islamic and historical periods was also identified. Notably, in one trench excavated to a depth of 4 metres, pottery fragments dating to the fourth millennium BC were discovered (Javeri, 2021). Additionally, bone remains were recovered from the stratigraphic layers. Zooarchaeological analysis conducted at the Bioarchaeology Laboratory of the University of Tehran identified these remains as belonging to goats, sheep, and cattle (Davoudi & Mashkour, 2020).

The sample MMCHB01 was included in this study and belongs to the Early Islamic period.

**28- Kākh-e Jahānnamā (Isfahan, Iran)** (32.656960°, 51.669178°)

Kākh-e Jahānnamā (Jahānnamā Palace, meaning *“World Showcase”* in English) was located on Chahar Bagh Street in the centre of Isfahan City, Isfahan Province, Iran. The palace was constructed during the Safavid period and was demolished in 1896 by Zell-al Soltan, the Qajar-era governor of Isfahan. Although the structure no longer exists, its location is documented in historical plans and maps of Charbagh Street, and several photographs dating to the Qajar period have survived. As part of recent urban development projects, including the construction of the subway system beneath Charbagh Street, the first archaeological investigations at the presumed site of Jahānnamā Palace were conducted between February and May 2015 by Ali Shojaee-Esfahani. The excavations covered approximately 300 m² and revealed not only Safavid-period architectural remains but also evidence of pre-Safavid and pre-Islamic occupations (Shojaee-Esfahani *et al*., 2017). Archaeozoological analyses carried out at the Bioarchaeology Laboratory of the University of Tehran identified a limited diversity of taxa. Domesticated sheep, goats, and cattle were the most exploited animals across all occupational phases at Jahānnamā Palace. A small number of bones from two-humped camels and boars were also identified. Identified bird remains indicating their presence in the site's subsistence practices (Khazaeli & Mashkour, 2019).

Samples MMKJN01 and MMKJN02 were included in this study and belong to the Late Islamic period.

**29- Masjed-e Jāmeʿ Isfahan (Isfahan, Iran)** (32.669444°, 51.684999°)

Masjed-e Jāmeʿ Isfahan, also known as the Great Mosque or Friday Mosque, is a UNESCO World Heritage Site located within the historic urban fabric of Isfahan in central Iran ([UNESCO World Heritage Centre](https://whc.unesco.org/en/list/1455/?utm_source=chatgpt.com): <https://whc.unesco.org/en/list/1397/>). Constructed in the 8th century CE on the remains of pre-Islamic settlements, the mosque’s long process of formation and transformation reflects more than a millennium of continuous urban life. The evolving ritual requirements and multiple functions of this monumental complex fostered dynamic interactions between communities and their urban and rural environments. Still an active place of worship, the Isfahan Friday Mosque embodies over twelve centuries of the city’s history, as well as the broader development of Islamic architecture in general and Iranian architecture in particular. Its central role in Persian architectural history—and especially in the monumental heritage of Isfahan—is evidenced by the successive rulers and patrons who left their marks on the building. Through continual modification and expansion, the mosque became an evolving architectural entity whose growth is closely intertwined with the development of the city itself. The complex expanded organically, both internally and into the surrounding urban fabric (Shojaee-Esfahani, 2018).

Major restoration and excavation campaigns at Masjed-e Jāmeʿ Isfahan were carried out in the 1970s by joint Iranian and Italian teams. In the frame of the collaboration between the Service for the Restoration and Preservation of the Historical Monuments of Iran (now the Ministry of Cultural Heritage, Handicrafts and Tourism) and the *Istituto Italiano per il Medio ed Estremo Oriente* (IsMEO, now IsIAO), the Italian Restoration Mission—led by Eugenio Galdieri—undertook restoration work between 1970 and 1972. Subsequently, from 1972 to 1978, an Italian Archaeological Mission conducted systematic research at the site in cooperation with the National Research Council of Italy (CNR) and the *Istituto Universitario Orientale* of Naples, directed by Umberto Scerrato and E. Galdieri (Galdieri, 1972, 1973, 1984). In 2003, the Archaeological Digital Archive of Masjed-e Jāmeʿ Isfahan (ADAMJI), a joint Iranian–Italian project led by Bruno Genito and Michael Jung, was launched to select and catalogue the large number of archaeological finds (Genito & Saiedi-Anaraki, 2011). In sum, based on the results of the mentioned archaeological and restoration investigations, nine major architectural phases have been identified: Abbasid I (8th century CE), Abbasid II (9th century CE), Buyid (10th century CE), Seljuk I (11th century CE), Seljuk II (11th–12th centuries CE), Muzaffarid/Ilkhanid (14th century CE), Timurid (15th century CE), Safavid (16th–17th centuries CE), and Qajar (19th century CE).

Archaeozoological studies of the exceptional faunal assemblage—comprising hundreds of thousands of animal bones excavated during the 1970s at Masjed-e Jāmeʿ Isfahan—have enabled our interdisciplinary research team to investigate food traditions in Isfahan, alongside broader questions of environmental change, economic networks, social differentiation, and belief systems within the cultural landscape of a major Islamic city. Osteological analyses indicate that three domestic herbivores—sheep, goats, and cattle—were the primary sources of meat consumption, supplemented by chicken. Sheep and goat (Caprini) remains account for approximately 70–80% of the assemblage across different periods. In addition, remains of equids (horse and donkey), camel, dog, fox, cat, rodent, and fish were identified (Mashkour *et al*. 2024a, 2024b).

Samples MMMJI01, MMMJI02, MMMJI03, MMMJI05, MMMJI07, MMMJI08, MMMJI09, MMMJI10, MMMJI11, MMMJI12, MMMJI13, and MMMJI14 were included in this study and belong to the Medieval/Islamic Era.

**30- Varzaneh 013** (32.36465°, 52.55208°)**, and 31- Varzaneh 051** (32.3563°, 52.56471°) **(Isfahan, Iran)**

Two Early Bronze Age sites, Varzaneh 013 (also known as Tappeh Gabri) and Varzaneh 051, were excavated during field seasons in 2018 and 2019. Both sites are located on the Varzaneh Plain, approximately 105 km southeast of the city of Isfahan, in Isfahan Province, central Iran. The area lies west of the Gavkhouni Playa (wetland) and is characterised by an arid to semi-desert landscape (Jebreili *et al*., in press). Varzaneh 013 is situated near the village of Kafarved, while Varzaneh 051 lies approximately 1,400 m southeast of Varzaneh 013. Varzaneh 013 covers an area of about 3.7 ha and rises approximately 1.8 m above the surrounding plain; Varzaneh 051 is similarly slightly elevated, by about 1 m. At Varzaneh 013, three excavation trenches revealed architectural remains and metallurgical evidence, whereas five trenches at Varzaneh 051 uncovered burial contexts, architectural and industrial materials. Together, the two sites provide evidence for copper and lead smelting activities in the region. Both comparative and absolute chronological data indicate that the primary occupation of Varzaneh 013 and 051 dates to the Early Bronze Age II (EBA II), approximately 2700–2200 BCE. Moreover, there is evidence indicating that these sites remained inhabited throughout the early second millennium BCE. The material culture shows strong affinities with Godin III:6–5 assemblages from the Posht-e Kuh and Pish-e Kuh regions, Susa IV, the Jalyan cemetery in the Fasa Plain, as well as stone vessels and beads from southeastern Iran. Overall, the evidence suggests a period of cultural and economic flourishing in the eastern Zayandeh-Rud River basin (Varzaneh Plain) between 2700 and 2200 BCE, contrasting with a contemporaneous decline in settlement density across other parts of the Central Iranian Plateau, such as Sialk and Arisman (Shojaee-Esfahani & Rafiei-Alavi, 2020; Rafiei-Alavi & Shojaee-Esfahani, 2020; Rafiei-Alavi *et al*., 2021a, 2021b; Kakavand *et al*., 2025).

Archaeozoological analyses were conducted at the Bioarchaeology Laboratory of the University of Tehran. Domesticated sheep and goats constitute the most frequently identified taxa at both sites, followed by domestic cattle. Equid remains may also include domestic donkeys, although this identification remains tentative. Wild species are relatively rare in the assemblages and include wild boar, deer, Persian onager, birds, and canids (Mashour *et al*., 2025).

For Varzaneh 013, samples MMVRZ3, MMVRZ4, and MMVRZ5, and for Varzaneh 051, samples MMVZ01, MMVZ02, MMVZ03, MMVRZ1, and MMVRZ2 were included in this study and belong to the Early Bronze Age.

**32- Qasr-e Ahmad (Fars, Iran)** (29.17061°, 52.771194°)

Tepe Qasr-e Ahmad is located along the Qara Aghaj River near the village of Kavar in Fars Province, within the Southern Zagros region of Iran. In the winter of 2011, the mound was excavated during a campaign by Mohammad Hossein Azizi Kharanaghi, during which three stratigraphic trenches were opened. The site covers approximately 6.5 ha and lies at an elevation of 1,515 m above sea level. Archaeological evidence indicates two distinct phases of Neolithic occupation at Qasr-e Ahmad. The earlier phase belongs to the Pre-Pottery Neolithic (PPN), dated to the mid-8th millennium BC, while the later Pottery Neolithic (PN) phase, characterised by the appearance of ceramic vessels, dates to the mid-7th millennium BC (Azizi Kharanaghi *et al*., 2012).

The zooarchaeological analysis was conducted at the Bioarchaeology Laboratory of the University of Tehran. Goats were the most intensively exploited animals during both Neolithic phases, whereas sheep were present in smaller numbers and made only a limited contribution to the subsistence economy. Cattle and pigs were neither domesticated nor regularly exploited. In addition to domesticates, wild taxa such as gazelle, wild sheep and goats, hare, and birds were hunted. The site’s proximity to the permanently flowing Qara Aghaj River, the presence of architectural remains in the PPN levels, and the abundance of lithic tools together indicate that both pastoral and agricultural activities formed integral components of the subsistence economy at Qasr-e Ahmad (Kamjan *et al*., 2018).

Samples MMQA01, MMQA03, MMQA06, MMQA07, MMQA08, MMQA09, and MMQA10 were included in this study and belong to the Pottery Neolithic period.

**33- Ali Tappeh Cave (Mazandaran, Iran)** (36.685833°, 53.587777°)

Ali Tappeh Cave (also known as Al-Tappeh Cave) is an important Epipalaeolithic/Mesolithic cave located on the northern flank of the Alborz Mountains in Mazandaran Province, northern Iran, facing the southeastern shores of the Caspian Sea. Originally excavated by C.B.M. McBurney in the 1960s, the site was initially attributed entirely to the Mesolithic period (McBurney & Payne, 1969). However, recent radiocarbon dating has refined the chronology: layers 1 to 11 (phases A–C) are now dated to the Mesolithic (approximately 11,200–9,700 cal BC), while the upper layers 12 to 23 (phases D–E) have been reattributed to the Early Neolithic (ca. 8,500–7,800 cal BC) (Arbez, 2018).

The faunal assemblage from the Mesolithic layers reflects a diverse hunter-gatherer economy adapted to a variety of local environments. Prey selection focused primarily on gazelle, followed by Urial sheep (*Ovis vignei*), aurochs, and wild boar. Several carnivores are also present, including lynx (*Lynx lynx*), wild cat (*Felis silvestris*), badger (*Meles meles*), and fox (*Vulpes vulpes*). A particularly notable aspect of the site is the significant exploitation of Caspian seal (*Pusa caspica*), highlighting the importance of marine resources — a feature linked to the site's proximity to the Caspian shoreline during periods of higher sea levels. Bird and fish remains were also recovered throughout the sequence, representing 21 different species in total. These remains reflect the exploitation of a wide range of biomes, including mountainous terrain, seacoast, marshes, and lowland areas. This diverse faunal spectrum underscores the role of Ali Tappeh as a key site for understanding human adaptations and the transition from hunting and gathering to early food production in the southeastern Caspian region (Uerpmann & Frey, 1981). The faunal remains from Ali Tappeh Cave are stored in the National Museum of Iran. As part of a later curation and research initiative in the 2010s (Mashkour *et al*., 2012, 2021), the animal bone assemblage from Ali Tappeh Cave was re-examined by Marjan Mashkour and Homa Fathi (Mashkour & Fathi, 2020).

Samples MMAT01 and MMAT02 were included in this study and belong to the Epi-Palaeolithic and Early Neolithic periods.

**34- Rafsanjan Region (Kerman)** (30.4066°, 55.99388°)

The skull of a modern wild sheep (mouflon, *Ovis orientalis*) analysed in this study was collected by local people from the Rafsanjan region, Kerman Province, southeastern Iran, and donated to Marjan Mashkour in 1995 for use as part of a comparative reference collection. The specimen is currently housed in the Bioarchaeology Laboratory at the University of Tehran. In this case, the animal had died naturally, possibly as a result of predation, and the skull and associated bones were subsequently collected from the site (Mashour, this study).

The sample MMRN01 from Rafsanjan was included in this study.

**35- Chalow (North Khorasan, Iran)** (37.673305°, 56.532333°)

The site of Tepe Chalow is situated in the easternmost part of the Jajarm Plain, approximately 3 km east of Sankhast and about 60 km west of Esfarayen. It lies at the terminus of the ancient delta of the Darband River (56°53′7.01″ E, 37°6′12.78″ N) at an elevation of 980 m asl. Tepe Chalow was excavated by A. A. Vahdati and R. Biscione. Recent excavations conducted by a joint Iranian–Italian team have revealed a necropolis containing both luxury items and ordinary household objects, as well as pottery closely comparable to those of the Greater Khorasan Complex. Pottery analysis, together with several radiocarbon dates, indicates a continuous sequence from the Late Chalcolithic to the Middle/Late Bronze Age, spanning from the end of the 4th millennium BC to the mid-3rd millennium BC (Vahdati *et al*., 2019). Archaeozoological analyses at the Bioarchaeology Laboratory of the University of Tehran highlight the importance of goats, sheep, and cattle, alongside hunted species such as hemione and gazelle. Of particular interest are the animal grave goods (Mashkour *et al*., 2016).

Samples MMCHC01, MMCHC02, MMCHC03, MMCHC04, MMCHC05, and MMCHC06 were included in this study and belong to the Late Chalcolithic period and Bronze Age.

**36- Khartouran Natural Park (Touran Biosphere Reserve)** (35.444752°, 56.569882°)

The Touran Biosphere Reserve covers an area of 1,464,992 hectares in Semnan Province, northeastern Iran. The reserve encompasses a wide variety of habitats, including three extensive plains at different elevations ranging from 700 to 1,400 metres above sea level, a saline river system, three mountain ranges rising to a maximum elevation of 2,200 metres, approximately 200,000 hectares of sandy landscapes with active dunes, and vast expanses of barren playa. The area was first designated as a protected region in 1976. Owing to its exceptional ecological importance—characterised by high levels of plant endemism and the presence of large mammals such as *Acinonyx jubatus*, *Panthera pardus*, *Felis catus*, *Felis margarita*, *Gazella subgutturosa*, *Gazella dorcas*, and *Equus hemionus*—the site was designated as a Biosphere Reserve under UNESCO’s Man and the Biosphere (MAB) Programme in 1977 (<https://whc.unesco.org/en/tentativelists/5275/>). The skull of a modern wild sheep (urial, *Ovis vignei*) analysed in this study was collected by local people from the region and donated to Marjan Mashkour in 1994 for use as part of a comparative reference collection. The specimen is currently housed in the Bioarchaeology Laboratory at the University of Tehran. The skull is housed in the Bioarchaeology Laboratory of the University of Tehran. The animal had died naturally, possibly as a result of predation, and the skull and associated bones were later collected from the site (Mashour, this study).

The sample MM12-623 from Khartouran was included in this study.

**37- Naderi (North Khorasan, Iran)** (37.395931°, 57.918598°)

Tepe Naderi is a 20 m-high artificial mound with a long occupational sequence, located in the Upper Atrak River valley, within the modern city of Shirvan in North Khorasan Province. In 2016 and 2018, the Iran–China Joint Expedition, directed by Ali Akbar Vahdati and Liangren Zhang, conducted two seasons of archaeological fieldwork at the site. Tepe Naderi consists of an imposing central mound surrounded by a dilapidated adobe fortification wall. In addition to two main excavation trenches (Trenches 1 and 2), the team opened twelve small test pits at the foot of the mound and along its periphery in order to clarify the stratigraphy and formation processes of the mound. The results of these two seasons indicate that Tepe Naderi was first settled in the late 4th millennium BCE (ca. 3500 BCE) and was occupied intermittently until the Late Islamic period. The excavations yielded a wide variety of artefacts and radiocarbon dates assigning the site to multiple cultural phases, including the Late Chalcolithic, Bronze Age, and Iron Age; the Achaemenid through Sassanid periods; a short medieval occupation dated to the 10th–11th centuries CE; and a final phase during the Qajar period (18th–19th centuries CE). During the Qajar period, a fortress was constructed atop the mound, overlooking a lower town enclosed by fortification walls. The Bronze Age occupation is characterised by a robust mud-brick defensive wall and ceramic assemblages closely comparable to those of the Namazga culture of Central Asia. Throughout prehistoric times, Tepe Naderi appears to have been closely connected to both the Namazga cultural tradition of southern Turkmenistan and the Burnished Grey Ware culture of the Gorgan–Damghan region. These findings suggest that the Shirvan area functioned as a zone of cultural interaction between Central Asia and the Iranian Plateau from the 4th to the 2nd millennium BCE (Vahdati and Zhang, 2019; Zhang *et al*., 2021; Vahdati *et al*., 2024).

Archaeozoological studies conducted at the Bioarchaeology Laboratory of the University of Tehran identified a diverse range of taxa. Domesticated sheep, goats, and cattle were the most exploited animals throughout all occupational periods. Horses, donkeys, and camels appear from the Bronze Age onwards. Wild taxa, including wild sheep and goats, boar, deer, Persian onager, as well as dogs, foxes, small carnivores, rodents, turtles, and birds, were also identified across multiple periods (Vahdati *et al*., 2024; Mashkour *et al*., 2025). Furthermore, analysis of chicken bones dating to the 18th–19th centuries CE revealed evidence for the presence of a virulent strain of Marek’s disease virus at Tepe Naderi (Fiddmann *et al*., 2023).

Samples TNS01, TNS02, TNS03, TNS04, TNS05, TNS06, TNS07, TNS08, TNS12, TNS13, TNS14, TNS15, TNS16, TNS17, TNS18, TNS19, MMTN20, MMTN21, MMTN23, MMTN24, MMTN25, MMTN27, MMTN28, MMTN29, MMTN30, MMTN32, MMTN33, and MMTN34 were included in this study and belong to the Bronze and Iron Ages and the Islamic Era.

**38- Kale Kub (South Khorasan, Iran)** (33.887194°, 58.373611°)

Tepe Kale Kub is located in the eastern region of the Iranian Plateau, in South Khorasan Province. The eastern fringes of the Iranian Plateau, particularly the South Khorasan Province, are still very little known archaeologically. Kale Kub, an exceptional site in a hyper-arid environment, was excavated over four seasons, from 2018 to 2023, by Mohammad Hossein Azizi Kharanaghi. The cultural deposits revealed a sequence of occupations from the Early Chalcolithic, 5^th^ mill. BCE to the Bronze Age, 3^rd^-2^nd^ mill. BCE. Notably, there is a 4^th^ mill. BCE phase contemporaneous to the Susa II horizon / Late Uruk, showing distinctive pottery and indicating cultural interactions between Mesopotamia and Iran. This represents the easternmost evidence of Uruk culture on the Iranian Plateau (Azizi, 2020; Azizi & Abe, 2024). The faunal remains are well preserved, and more than 10,000 bone fragments were studied at the Bioarchaeology Laboratory, Central Laboratory of the University of Tehran. The archaeozoological analysis showed that sheep (*Ovis aries*) and goat (*Capra hircus*) constitute the bulk of the exploited animal resources in all periods (67.5%), where goat outnumbers sheep. Wild herbivores were also hunted by the inhabitants, as indicated by gazelle (*Gazella sp.*) and hemione (*Equus hemionus onager*). Most equid bones from the metric analyses and logarithmic profiles are identified as hemiones, though some may belong to horses or donkeys (Amiri *et al*., 2025).

Samples MMKK01, MMKK02, MMKKB01, MMKKB08, and MMKKB10 were included in this study and belong to the Early Chalcolithic period.

**39- Nishāpur (Khorasan Razavi, Iran)** (36.171111°, 58.847194°)

Nishāpur, one of the principal urban centres of Khorasan in northeastern Iran, was founded during the Sassanian period and has remained continuously occupied to the present day (1198 m asl; 36°10′16.72″ N, 58°50′50.82″ E). Evidence of this long-term occupation is preserved across a vast area corresponding to the historical urban landscape of Nishāpur. At its zenith, between the 10th and 12th centuries CE, the city supported the production of a wide range of agricultural food and non-food commodities, with processed foods forming an integral part of the local economy (Lorzadeh & Laleh, 2018). Following extensive archaeological surveys in 2009-2010, and since 2018, a long-term archaeological project led by Haeedeh Laleh and Zahra Lorzadeh sought to establish the city’s chronology and to reconstruct the formation and development of its urban landscape through time (Laleh *et al*., 2015). The first excavation season in 2018 uncovered a diverse assemblage of artefacts from 15 trenches (Laleh & Lorzadeh, 2019). Additionally, 2,470 animal bone fragments from a faunal assemblage initially collected during the 2005–2006 seasons of joint Franco-Iraninan excavation within the citadel (*Kohandej*) were analysed in the Archaeozoology Section of the Bioarchaeology Laboratory at the University of Tehran. These remains date from the Sassanian period through the 12th century CE. The results indicate that sheep, goats, and cattle were the primary animals exploited, providing both meat and secondary products (Khazaeli *et al*., 2021).

The sample MMNIK01 was included in this study and belongs to the Islamic period (11^th^ -12^th^ Century CE).

**40- Graziani (Sistan and Baluchestan, Iran)** (30.470686°, 61.278339°)

Tepe Graziani (also spelt Tappeh Graziani) is situated in Sistan and Baluchestan Province, approximately 56.6 km south of Zabol and about 10 km west of the major Bronze Age centre of Shahr-i Sokhta, in the southern Sistan Plain. The region is characterised by a hot desert climate, with past environmental conditions sustained by the terminal branches of the Helmand (Hirmand) River. The site extends over roughly 3 hectares and rises about 10 m above the surrounding plain. Graziani represents an important Bronze Age archaeological site in southeastern Iran and is generally interpreted as a satellite industrial settlement associated with the urban centre of Shahr-i Sokhta. As such, it plays a key role in reconstructing the economic organisation and administrative complexity of the Helmand Civilisation. Archaeological investigations were carried out over three excavation seasons initiated in 2010 by Hossein Ali Kavosh of the University of Zabol, with the aim of clarifying the site’s architectural layout and industrial activities. The cultural deposits at Tepe Graziani are closely integrated into the broader chronological framework of Shahr-i Sokhta and correspond to Periods II, III, and IV. Radiocarbon dating places the occupation between approximately 2860 and 2300 BCE, after which the site—like other major settlements in the region—was abandoned around 2300–2200 BCE (Kavosh *et al*., 2019; Kavosh & Oveisi-Keikha, 2024). Archaeozoological analyses of faunal remains from Tepe Graziani were conducted at the Bioarchaeology Laboratory of the University of Tehran. The assemblage is overwhelmingly dominated by caprines (sheep and goats), which account for approximately 92% of the identified specimens (NISP). In contrast, cattle (Bos sp.) are represented by an unusually low proportion of the assemblage (0.7%), and it is currently not possible to distinguish between taurine and zebu cattle. Wild taxa are limited and include gazelles, birds, and fish. Overall, the restricted faunal spectrum underscores the central role of sheep and goat husbandry in animal resource exploitation at Graziani, while cattle played only a minor role, and hunting and fishing contributed marginally to subsistence strategies (Mashkour *et al*., 2019).

Samples MMTG02, MMTG03, MMTG04, MMTG05, MMTG07, MMTG08, MMTG12, MMTG13, MMTG14, MMTG15, and MMTG16 were included in this study and belong to the Early Bronze Age.

**41- Dahan-e Gholaman (Sistan and Baluchestan, Iran)** (30.797026°, 61.636575°)

Dahan-e Gholaman is a unique archaeological site in southeastern Iran and is widely recognised as an Achaemenid city constructed according to a pre-planned urban design. The site is located approximately 44 km southeast of the city of Zabol in Sistan and Baluchestan Province, about 2 km south of the village of Qalʿe-No, within the Sistan basin and close to the present-day border with Afghanistan (Gnoli, 1993). The region is characterised by an arid desert climate with extreme summer heat and strong seasonal winds. Historically, the settlement depended on the waters of the Helmand (Hirmand) River, and its eventual abandonment is generally attributed to the desiccation of a river branch or a sudden shift in its course (Scerrato, 1962; Mariani, 1977). The site was first systematically excavated by the Italian Archaeological Mission of the *Istituto Italiano per il Medio ed Estremo Oriente* (IsMEO), directed by Umberto Scerrato, between 1962 and 1965/1966 (Scerrato, 1966). A second major phase of excavation began in 2000 and continued until 2005 under the direction of Iranian archaeologist Mansour Seyed Sajjadi. The core excavated area covers approximately 12 hectares (Sajjaddi, 2006, 2019). Archaeological evidence indicates that the main occupation of Dahan-e Gholaman dates to the Achaemenid period, particularly the 6th and 5th centuries BC (c. 550–330 BC). The stratigraphic sequence suggests a relatively short occupation span—approximately 200 years—followed by an organised and deliberate abandonment of the site (Genito, 1990). Dahan-e Gholaman is exceptional among Achaemenid settlements, as the site displays a defined grid-like urban plan, with residential quarters systematically arranged alongside public, administrative, and religious buildings. Dahan-e Gholaman was constructed entirely of mudbrick and clay. Architectural features such as domed and arched roofs reflect local building traditions and practical adaptations to the harsh climatic conditions of the region (Mariani, 1977). The site is commonly identified with Zranka (or Zarin), the capital of the Achaemenid satrapy of Drangiana (Gnoli, 1993).

Faunal remains recovered during the 1960s excavations were subsequently stored in the National Museum of Iran. As part of a later curation and research project in the 2010s, the animal bone assemblage from Dahan-e Gholaman was studied by Marjan Mashkour and Homa Fathi (Mashkour *et al*., 2012, 2021). The assemblage is dominated by domesticated caprines (sheep and goats), which constitute more than 85 percent of the identified remains, followed by cattle. In addition, remains of camelids, gazelle, deer, boar, canids, rodents, and birds were also identified, reflecting both pastoral practices and exploitation of local wild fauna (Mashkour & Fathi, 2016).

Samples MMDQN01-L, MMDQN01-R, MMDQN02-L, MMDQN02-R, MMDQN03-L, and MMDQN04 were included in this study and belong to the Achaemenid Empire.

**Southern Central Asia (Delphine Decruyenaere)**

All Southern Central Asian samples analysed in this study derive from archaeological sites located in the Bukhara and Samarkand oases, situated in the middle and lower courses of the Zarafshan Valley in the northwestern part of ancient Sogdiana (present-day southeastern Uzbekistan) (Gulyamova, 2022). Rising in the Zarafshan Range, part of the Pamir-Alay mountain system in present-day Tajikistan, the Zarafshan River flows westward toward the piedmont, lowland plains, and deserts of Uzbekistan, providing an important water source within an otherwise semi-arid environment characterised by a sharply continental climate, with hot, dry summers, cold winters, and limited rainfall (Khojimatov et al., 2023; Gintzburger et al., 2003). It thus sustains the oases of Samarkand and Bukhara, where fertile soils and long-established irrigation systems have supported agriculture and dense human occupation for millennia (Fouache et al., 2016; Fouache et al., 2020; Mantellini et al., 2018; Rante & Mirzaakhmedov, 2019).

The samples considered in this study were collected as part of D. Decruyenaere’s PhD that aims to investigate and reconstruct long-term patterns of diet and animal resource management in the Zarafshan Valley (Decruyenaere et al., 2024, Begmatov et al., 2024, Begmatov et al., in press). The samples derive from archaeological sites spanning different chronological phases and cultural horizons, all dating to the Antiquity and Medieval periods, and representing a wide range of contexts (including urban and rural settlements, fortresses, and religious complexes) as well as diverse spatial settings, from central to peripheral locations within oasis systems and along primary and secondary trade routes.

Across all archaeological sites dealt with in this paper, the faunal spectra show continuity over time, with no major chronological shifts. They are consistently dominated by the standard domestic triad, with caprines (sheep – *Ovis aries* and goat – *Capra hircus*) overwhelmingly predominant and cattle (*Bos* sp.) present in smaller proportions (Decruyenaere et al., 2024; Begmatov et al., 2024). This pattern can also be observed at all neighbouring Antique and Medieval settlements for which archaeozoological studies have been published (e.g., Serone et al., 2021; Portero et al., 2022). The only notable diachronic change concerns suids, whose relative representation declines over time, already from the first centuries CE onwards.

Bovids are systematically associated with remains of equids (horses, donkeys, and their hybrids), which were used in multiple ways, including riding, pack transport, and traction; however, in light of the abundant butchery marks observed on their bones, they were also probably consumed. By contrast, camelids, whether two-humped camels, dromedaries, or their hybrids, despite their iconic association with the *Silk Roads*, are not attested at all sites and, when present, occur only in small proportions. Remains of commensal animals are also attested in most of the settlement, including rodents and dogs (*Canis familiaris*). The contribution of wild taxa to the assemblages is minimal in large urban settlements, representing less than about 3% of the faunal spectra. A relatively higher proportion of wild species is observed at Kakishtuvan, a settlement located on the periphery of the Bukhara oasis, at the desert fringe, where environmental conditions likely encouraged greater exploitation of wild resources.

**The Oasis of Bukhara**

The oasis of Bukhara, together with its southwestern extensions, the Qaraqöl micro-oasis, lies along the final course of the Zarafshan River and covers an area of approximately 5,000 km² (Rante & Mirzaakhmedov, 2019; Gulyamova, 2022). It lies at the edge of the Kyzylkum Desert, within the Chol zone, characterised by arid plains and desert-steppe environments dominated by shrubs and herbaceous species such as *Alhagi maurorum*, *Artemisia* sp., *Carex pachystylis*, *Poa bulbosa* or *Peganum harmala* (Khojimatov et al., 2023).

The earliest occupation of the oasis dates to around the 3rd century BCE, when the progressive drying of formerly marshy lands created conditions favourable for permanent settlement (Rante, Fouache & Mirzaakhmedov, 2016; Rante & Mirzaakhmedov, 2019). Earlier sites, by contrast, have been identified mainly on the periphery of the oasis or in the surrounding desert regions (Stark et al., 2024; Rouse et al., 2026). In 2009, the Franco-Uzbek archaeological mission (MAFOUB), a joint project involving the Archaeological Institute of Samarkand and the Department of Islamic Arts of the Louvre Museum with the collaboration of the Institute of Iranian Studies of the Austrian Academy of Sciences, initiated research in the Bukhara Oasis. The project investigates the dynamics of human occupation, together with socio-cultural, economic and environmental changes. In line with this objective, the mission has conducted a systematic survey of the oasis and investigated seven of its principal archaeological sites, including Bukhara, Paykend, Varakhsha, Ramitan, Iskijkat, and Kakishtuvan, which yielded samples analysed in this study (Rante & Mirzaakhmedov, 2019; [Rante](https://brill.com/search?f_0=author&q_0=Rocco+Rante), [Schwarz](https://brill.com/search?f_0=author&q_0=Florian+Schwarz) & [Tronca](https://brill.com/search?f_0=author&q_0=Luigi+Tronca), 2022; Rante, 2024).

**42- Paykend** (39.586080°, 64.008220°)

*Paykend* is situated on the western periphery of the Bukhara Oasis, near the Qaraqöl micro-oasis, along the course of the Zarafshan River. The settlement comprises a citadel, two shahristan areas (lower cities), and surrounding suburbs. Initial human occupation occurred within the area that later became the citadel, dating to approximately the early 3rd century BCE. The expansion of the city saw the addition of shahristan-1 in the second half of the 4th century CE and shahristan-2 between the late 6th and early 7th centuries CE. By the 9th century, the southern and eastern suburbs had developed further. Paykend remained inhabited throughout the Islamic period until the Timurid era (14th–15th centuries), though it experienced a gradual decline in population from the 12th century onwards. Since the 19th century, extensive archaeological and historical investigations have been carried out at the site. The MAFOUB mission initiated its research at Paykend in 2009, conducting stratigraphic soundings and large-scale excavations in the citadel (trench D), shahristan-1 (trenches A, B, C), shahristan-2 (trench F), the eastern suburbs (trench G), and the artisanal quarter (trench E).

Rante & Raimkulov, 2013; Rante & Mirzaakhmedov, 2019; Rante, 2021; Omel’chenko & Mirzaakhmedov, 2024; Puschnigg & Bruno, 2024; Richardin & Rante, 2024. Amongst the earlier works on Paykend, see Mukhamedzhanov et al. (1988).

The samples PA183 and PA413 were included in this study.

**43- Varakhsha** (39.863658°, 64.073005°)

Varakhsha lies at the western margin of the present-day Bukhara oasis. The earliest phase of occupation was initially dated to between the 2nd century BCE and the 1st century CE; however, recent archaeological evidence and C14 dates suggest an even earlier occupation (Rante, forthcoming). The site is organised around a citadel, where a large palace complex was uncovered, surrounded by an extensive shahristan and an outer suburb. From the 3rd to the 5th centuries CE, Varakhsha developed into a strongly fortified urban centre, followed by renewed monumental expansion during the 7th–8th centuries. From the 9th century onwards, the settlement experienced a gradual demographic decline, culminating in progressive abandonment between the 12th and 13th centuries, likely linked to the reduction or loss of local water resources. A stratigraphic trench (Trench A) was excavated in the shahristan by the MAFOUB mission between 2023 and 2024.

Shishkin, 1963; Naymark, 2003; Rante & Mirzaakhmedov, 2019.

The sample VK003 was included in this study.

**44- Kakishtuvan** (40.047633°, 64.156441°)

Kakishtuvan is situated at the north-western edge of the Bukhara oasis, along the axis connecting Bukhara to Khoresm. Like the other major tripartite settlements of the oasis, the site comprises a rectangular, fortified citadel, a shahristan, and an extensive suburb. The earliest occupation, evidenced in the citadel area, dates to the 1st century CE, as revealed by the stratigraphic Trench A. Survey data indicate that the shahristan developed alongside the citadel only by the 3rd–4th centuries CE. The 5th century witnessed intensified settlement and significant construction activity. Stratigraphic data indicate a gradual decline in citadel occupation during the 6th–7th centuries, followed by slow depopulation in the early years after the Islamic conquest. Subsequent traces of habitation are visible between the 9th and 12th centuries, with sporadic use continuing into the Timurid period.

Rante & Mirzaakhmedov, 2019; Puschnigg & Bruno, 2024; Richardin & Rante, 2024.

The sample KT121 was included in this study.

**45- Ramitan** (39.910861°, 64.2691°)

Ramitan is a tripartite urban settlement located in the central part of the oasis, comprising a walled citadel, a shahristan, and an unwalled suburb. Following limited excavations conducted by Suleymanov in the early 1970s, who opened a single stratigraphic trench within the city, the site has been surveyed and excavated by the MAFOUB expedition since 2011. These investigations include three stratigraphic trenches (A, C, and F), as well as more extensive excavations in the citadel (trench D) and the suburb (trench G). The earliest occupation corresponds to a non-urban settlement dating to the 3rd–2nd centuries BCE. Ramitan adopted a quadrangular urban layout in the 1st century CE, which was expanded and fortified during the 2nd and early 3rd centuries CE. In the 4th century CE, the city developed further with the construction of a shahristan to the south of the citadel, followed by the emergence of an unwalled suburb to the west in the late 4th–5th centuries. Between the 5th and 7th centuries CE, Ramitan reached its peak as a well-organised urban centre covering approximately 24 hectares, a configuration that persisted into the early Islamic period. During the 8th–9th centuries CE, urban development shifted toward the southwestern part of the site, where a new urban unit was established, including an unwalled citadel and a new shahristan to the north. Occupation continued throughout the Islamic period until the Timurid era (14th–15th centuries). Archaeological evidence also indicates Timurid and post-Timurid occupations (16th–17th centuries), particularly above the city and in the western sector of the site, which was subsequently reused as a modern cemetery.

Suleymanov, 1984; Rante & Mirzaakhmedov, 2019; Puschnigg & Bruno, 2024; Richardin & Rante, 2024.

The samples RM001 and RM002 were included in this study.

**46- Bukhara** (39.863658°, 64.073005°)

Bukhara represents the principal settlement of the oasis and has served as its capital since the early Islamic period. The city displays a classic tripartite urban layout, consisting of a citadel, a shahristan, and surrounding suburbs. The earliest evidence of occupation, identified within the citadel, dates back to the 3rd century BCE. From the 3rd century BCE to the 1st century CE, the settlement was organised around the citadel and an eastern shahristan, which has remained the enduring urban core of the city. Demographic growth is attested from the 2nd–3rd centuries CE onwards, followed by a sustained expansion of the shahristan and suburban areas over subsequent centuries, culminating in the early Islamic period (9th–10th centuries). At this time, Bukhara reached its first major political apogee as one of the most important centres of the Samanid state. Urban growth continued throughout the Timurid period (14th–15th centuries), prior to major transformations of the city during the 16th and 17th centuries. Archaeological investigations conducted by the MAFOUB expedition include one stratigraphic trench (A) in the Citadel and a larger excavation (trench C), as well as additional trenches (B and D) within the shahristan.

Mukhamedzhanov, Mirzaakhmedov & Adylov, 1982; Nekrasova, 1999; Gangler, Gaube & Petruccioli, 2004; Rante & Mirzaakhmedov, 2019; Puschnigg, Bruno, 2024; Richardin & Rante, 2024.

The samples BK160, BK433, BK477 and BK478 were included in this study.

**47- Iskijkat** (39.970936°, 64.618497°)

Further east, Iskijkat is situated near the core of the oasis. Again, the site follows a tripartite layout, comprising a square citadel, an unwalled shahristan, and surrounding suburbs. Two deep stratigraphic trenches (A and B) were excavated, one within the citadel and the other in the shahristan. The earliest occupation of the citadel dates to the 3rd–2nd centuries BCE, followed by well-defined construction phases during the 2nd–1st centuries BCE and again in the 2nd–3rd centuries CE. From the 4th–5th centuries CE through to the 7th century, the citadel, shahristan, and suburbs display a clear continuity in occupation and urban organisation. During the 7th–8th centuries CE, expansion beyond the tripartite core suggests renewed demographic growth. From the early Islamic period (9th–10th centuries CE), occupation became largely concentrated in the citadel and shahristan, while the suburbs appear to have functioned primarily as areas of market activity and production. According to current evidence, occupations dating from the 12th to the 16th centuries CE are restricted to the citadel and reflect a marked decline in settlement activity, particularly from the 13th century onwards.

Rante & Mirzaakhmedov, 2019; Puschnigg & Bruno, 2024; Richardin & Rante, 2024.

The sample ISK469 was included in this study.

**The Oasis of Samarkand**

The oasis of Samarkand lies within a fertile alluvial plain in the middle Zeravshan Valley, covering approximately 5,000 km² (Mantellini, 2017; 2018). Situated between 900 and 500 m above sea level, it is largely encompassed by the Adyr zone, a transitional landscape of piedmont hills and steppe environment forming an interface between the desert lowlands and the surrounding mountain ranges (Khojimatov et al., 2023). The natural environment is characterised by ephemeral and dry steppe communities, with significant shrub and dry forb elements (Khojimatov et al., 2023, Gintzburger et al., 2003). The fertility of the alluvial plain, strengthened by an extensive network of artificial canals, most notably the Dargom to the south and the Bulungur to the north, supported the development of intensive irrigated agriculture. Semi-mobile pastoralism in the adjacent steppes, the foothills, and higher mountain pastures complemented farming activities (Mantellini, 2018).

The samples analysed in this study originate from three late antique and medieval archaeological sites: Kafir-kala, located in the southeastern vicinity of Samarkand; Kurgan-tepa, situated approximately 18 km southeast of Kafir-kala; and Suleyman-tepa, positioned about 11 km further southeast of Kurgan-tepa within the Urgut district.

*Kafir-kala* has been under systematic excavation since 2013 as part of a joint Uzbek-Japanese project led by the Samarkand Archaeological Institute and the Tezukayama University (Nara), followed by the National Museum of Ethnology (Osaka), with the subsequent involvement of the Brandenburg Academy of Sciences and Humanities (Berlin). Research has focused on the citadel (continuing the excavations previously conducted by the Uzbek–Italian mission) and the northwestern section of the shahristan, with particular attention to the Sogdian period and its transition to the early Islamic era. Excavations have uncovered what is believed to be a ritual complex dedicated to the goddess Nana, a grand hall presumed to be part of the palace quarter of the Sogdian kings, as well as significant findings related to pre-Islamic material culture (Begmatov et al., 2020 and 2024; Berdimuradov et al., 2018; Grenet, 2020; Murakami et al., 2023).

Since 2022, with the objective of investigating urban emergence and cultural development, as well as preventing the destruction of sites in the presumed Maymurgh principality, one of the Sogdian city-states, another Uzbek–Japanese–German team has launched a joint research project at *Kurgan-tepa,* conducted concurrently with investigations at Kuldor-tepa in its northern vicinity. The Japanese component was, this time, initially affiliated with Hokkaido University, followed by Tohoku University of Arts and Design, and most recently Okayama University. Excavations uncovered a rectangular cella with a central altar and murals, indicating the presence of a Sogdian temple (Murakami et al., 2023a; 2024; Begmatov et al., 2025).

Finally, the medieval site of Suleiman Tepa has been under excavation since 2022 by an international team comprising specialists from Uzbekistan, Kazakhstan, and the United States. The project is conducted under the direction of the International Institute for Central Asian Studies (IICAS, UNESCO) and the Samarkand Archaeological Institute. Its primary objective is to investigate the monastery and its associated necropolis in order to document the history and establishment of the Church of the East along the Silk Road (Archaeological Report, 2022, 2023, 2024; Gilbert, 2024; Gilbert et al., forthcoming).

**48- Kafir-Kala** (39.955591°, 67.037516°)

Kafir-kala is located approximately 10 km southeast of Afrasiab (ancient Samarkand), along the Dargom canal, covering roughly 16 ha. Naturally protected by rivers on all sides, the site forms a monumental and prominent landmark, rising c. 25 m above the surrounding plain. Identified as the residence of the Sogdian kings, its position on the routes from Kesh/Shahri-Sabz made it a direct stronghold of Afrasiab, controlling the southern approach to the capital. The settlement comprises a fortified citadel encircled by a moat and towers. The moat is reinforced by four earth ramparts, with the shahristan situated atop them, while a second moat in the northwest separates the city from the suburb. South of the complex was the necropolis. The Uzbek-Japanese expedition, active since 2013, initially opened a trench at the summit of the citadel (Trench 1) and has focused on the shahristan (Trenches V and IX) since 2019. Trench V, located in the northwestern part of the Shahristan, has yielded a large hall, interpreted as part of a palace complex.

The earliest securely dated occupation of Kafir-kala falls in the 4th century CE, with major expansion between around the 5th and early 8th centuries. The site may have been damaged or destroyed by fire in 712 CE during the campaigns of Qutayba ibn Muslim, as indicated by a widespread burn layer. Stratigraphic evidence from the grand hall reveals a phase of reoccupation following this event, which ended abruptly with another destructive fire around the mid-8th century. Settlement continued intermittently until the 18th century, with marked fluctuations during the Abbasid and Qarakhanid periods

e.g., Mantellini & Berdimuradov, 2005; Begmatov et al., 2016; Mantellini et al., 2017; Usami et al., 2017; Begmatov et al., 2020; Murakami et al., 2021; Begmatov, 2022; Bogomolov et al., 2022; Murakami et al., 2022; Berdimuradov et al., 2023; Murakami et al., 2023a; Murakami et al., 2023b; Begmatov et al., 2024; Teramura et al., 2024; Begmatov, 2026.

The samples KK001, KK007b, KK247, KK248, KK321a, KK321b, KK341, KK355 and KK460 were included in this study.

**49- Kurgan Tepa** (39.449338°, 67.149960°)

Kurgan-tepa is located in the Urgut district, approximately 28 km southeast of the pre-Mongol remains of Samarkand (Afrasiab). Today, only a portion of the original settlement has been preserved, mainly the roughly square citadel and the surrounding area, corresponding to the inner part of the shahristan.

Excavations in this area have revealed a series of interconnected rooms with arched ceilings encircling the citadel, dating to the 7th–8th centuries CE. These structures appear to have been abandoned by the early 8th century, with resettlement occurring later, particularly during the 11th–12th centuries CE. Trench 4, opened in 2023 within the innermost area of the citadel, confirmed the later phase of occupation and, in lower strata, revealed a rectangular cella (c. 8 × 5 m) featuring a central altar base, mural fragments on the walls and floor, and the lower portions of statues set in niches. These deposits are preliminarily dated to the 6th century and bear evidence of destruction by a major fire in the early 8th century CE.

Murakami et al., 2023c; Sandiboev et al., 2024; Murakami et al., 2024; Begmatov et al., 2025; Murakami et al., 2025; Sandiboev, 2025.

The sample UG028 was included in this study.

**50- Suleiman Tepa** (39.381029°, 67.241974°)

Suleyman Tepa is located approximately 40 km southeast of Samarkand, on a promontory at the foothills of the Zarafshan Mountains in Urgut. The site comprises a church, an adjacent cemetery, and scattered rock inscriptions distributed across the surrounding landscape. Investigations have made it possible to establish a clear association between these elements, confirming their integration within a Christian monastic complex.

Excavations of the church have brought to light an architectural plan closely comparable to other known ecclesiastical layouts of the Church of the East. The cemetery contains exclusively adult male burials, tentatively interpreted as members of the monastic community. All are orientated west–east and laid in a supine position with hands crossed over the chest, in accordance with Christian funerary practices. Artefacts are scarce; however, a silver cross recovered from one grave strengthens the connection between the church and the cemetery. Christian Syriac inscriptions on the adjacent rock caves point to a community shaped by multiple ethnic influences and may correspond to the locality mentioned by the 10th-century Arab geographer Ibn Hawqal. Chronological evidence derived from ceramics, coins, and radiocarbon dating indicates continuous occupation of the site from the 7th to the 13th centuries.

Savchenko, 1996, 2005, 2010; Dickens, 2017; Archaeological Report, 2022, 2023, 2024; Gilbert, 2024; Gilbert et al., forthcoming.

The sample ST024 was included in this study.

**Indus Valley, Pakistan (Aurore Didier, Marjan Mashkour)**

**51- Chanhu-daro** (26.173653°, 68.323101°)

Chanhu-daro is an ancient archaeological site of the Indus Valley Civilisation located in the Shaheed Benazirabad (Nawabshah) District of Sindh, Pakistan, about 130 km south of Mohenjo-daro and roughly 12 miles east of the present Indus River bed near the village of Jamal-Kirio. The site comprises three low mounds that together form a single settlement covering roughly 5–7 hectares, and it was inhabited from approximately 4000 BCE to 1700 BCE. Chanhu-daro is especially noted as a craft production centre, particularly for carnelian bead manufacturing, and shows evidence of planned urban features of Harappan settlements. Large-scale excavations from 2016 have resumed under the French Archaeological Mission in the Indus Basin (MAFBI) in collaboration with the Directorate General of Antiquities and Archaeology, Culture, Tourism, Antiquities and Archives Department, Government of Sindh in Pakistan, revealing stratified architectural levels, craft workshops, pottery, and other material culture that shed light on both the Harappan and subsequent Jhukar phases at the site (Didier, 2017 & in press; Didier *et al*., 2017, 2024). The rich faunal remains from the site indicate exploitation of bovids, possibly zebu cattle, and caprines, mainly goats and pigs. Fish remains are also very abundant in the osteological remains (Mashkour *et al.*, 2025).

**References**

Aali A, Abar A, Boenke N, Pollard M, Rühli F, & Stöllner T. 2012 Ancient salt mining and salt men: The interdisciplinary Chehrābād Douzlākh project in north-western Iran. *Antiquity*, Project Gallery 86(333). <http://antiquity.ac.uk/projgall/aali333>.

Abdollahi M, & Sardari A. 2013 Eastern Central Zagros during the Neolithic period: based on the excavation at Tepe Ghala Gap. *Journal of Archaeological Researches of Iran* *4*: 117–138.

Abdollahi M, Niknami KA, Hesari M, & Sardari Zarchi A. 2014 Village life and Culture transformations in the east Central Zagros Societies: archaeological excavations at Tepe Ghala Gap. *Journal of Archaeological Studies* *6*(1): 67–86.

Abedi A, and Omrani B. 2015 Kura-Araxes Culture and North-western Iran: New Perspectives from Kul Tepe Jolfa (Hadishahr). *Paléorient* 41(1): 55-68.

Abedi A. 2017 Iranian Azerbaijan pathway from the Zagros to the Caucasus, Anatolia and Northern Mesopotamia: Dava Göz, a new Neolithic and Chalcolithic site in NW Iran. *Mediterranean Archaeology and Archaeometry* 17: 69–87.

Ahmadzadeh L, Yashmi R, Mohammadkhani K, Omidfar M, Zadeh Dabbagh M, Heydarizadeh B. 2021 Exposing Haute Terrasse and Systematic Excavation in Susa Acropole . In: *Proceedings of 18th Annual Symposium on the Iranian Archaeology 2021*, R. Shirazi (ed.), Tehran: RICHT: 49-60 (in Persian).

Amiri S, Mashkour M, Mohaseb A, Tengberg M, Abdolahi M, & Sardari A. 2019 The Subsistence Economy of Qela Gap; Lurestan, Iran: From the Late Neolithic to the Iron Age. *Archaeology* *3*(4): 125–132.

Amiri S, Fathi H, Azizi Kharanaghi MH, Abe M, Davoudi H, and Mashkour M. 2025 Surviving at the edge of the Lut desert: Archeozoological analysis of the Kale Kub, a possible Uruk outpost in Eastern Iran. *ICAZ Working Group „Archaeozoology of Southwest Asia and Adjacent Areas“, ASWA XVIth International meeting*, 25-28 May 2025, Munich, Germany.

Arbez L. 2018 Faune et environnement passés du Sud de la Mer Caspienne : Analyse paléoécologique et taphonomique des restes de micromammifères du site d’Ali-Tappeh. Master 2 Thesis, Spécialité Quaternaire et Préhistoire, direction Marjan Mashkour, Emmanuelle Stoetzel. MNHN France.

Azizi Kharanaghi, M.H., Khalouei, F., Khanipour, M., Khosravi, S., Razi, S., Komijani, S. and Tahmasebi, M. 2012. The First Season of Archaeological Excavation at Tell-e Qasr e Ahmad, Kavaar District, winter 2011, Fars. In, Moggadam, A. and Roustaei, K. (eds) Abstracts Volume of the 11th Annual Symposium of Iranian Archaeology. Tehran: Iranian Cultural Heritage Handicraft & Tourism Organisation- ICHHTO.

Azizi Kharanaghi MH, Niknami K. 2013 The Late Chalcolithic Period at Tepe Sagzabad. *Payam-e Bastanshenas* 20: 51-68.

Azizi Kharanaghi MH, Jamshidi Yeganeh S, Abe M, and Akbari A. 2020 New Evidence of Beveled Rim Bowls from Kale Kub, South Khorasan Province. *Parseh Journal of Archaeological Studies* 4(12): 29-48.

Azizi Kharanaghi MH, Mashkour M, Beizaee Doost S, Jamshidi Yeganeh S, Akbari A. 2020 Evidence for fishing and collect of marine unvertebrates. in F. Biglari, J. Nokandeh, AM. Naderi Beni, A Hojabri, *Human and Sea: A Review of Thousands of Years of Relationships between the Human and Sea in Iran*. Pp.32-34, Tehran: National Museum of Iran.

Azizi Kharanaghi MH. 2022 The first results of excavation at Tol-e Sozu of Genaveh, Bushehr. *Parseh Archaeological Studies* 19: 33-51 (in Persian). 10.30699/PJAS.6.19.33

Azizi Kharanaghi MH, & Abe M. 2024 Eastern Iran Prehistoric Archaeological Project; The Second Seasons of Archaeological Excavation in Kale Kub, South Khorasan Province (2020). *Journal of Greater Khorasan* 15(55): 1-28. [10.22034/jgk.2023.403121.1129](https://doi.org/10.22034/jgk.2023.403121.1129).

Begmatov A, Uno T, Murakami T, Berdimurodov A, Bogomolov G, Teramura H, Usami T. 2016 Excavations of the Uzbek-Japanese Expedition on the Site of Kafirkala. *International Conference on Archaeology of Uzbekistan during the Years of Independence: Progress and Perspectives*, Samarkand, 116-118.

Begmatov A. 2022 Cross-Cultural Exchange across Eurasia as Reflected in the Sealings from Kafir-Kala in Samarkand. *Cultures in Contact: Central Asia as Focus of Trade, Cultural Exchange and Knowledge Transmission* (eds Ch. Baumer, M. Novák, S. Rutishauser), Schriften zur vorderasiatischen Archäologie, Wiesbaden: Harrassowitz 19, 193–203.

Begmatov A, Hirofumi T, Decruyenaere D, Mashkour M, Mir-Makhamad B, Spengler R, Murakami T, Sandiboev A, Takao U. 2024. The Grand Hall of Kafir-kala: An Observation of Archaeological Finds, Botanical and Zoological Evidence. *History and Archaeology of Turan* 6, 194-206.

Begmatov A, Murakami T, Sandiboev A. 2025 Discovery of a Temple at Kuldor-Tepa in Uzbekistan. Asian Review of World Histories 14/1, 5-25. (doi.org/10.1163/22879811-bja10079)

Begmatov A, Rahmonov H, Usami T, Decruyenaere D, Mir-Makhamad B, Spengler R, Mashkour M. 2026 From Late Antiquity to Early Medieval Sogdiana: A Glimpse from Ming-tepa. In *Mobility and Archaeogenetics in Central Asia People – Goods – Ideas* (eds Ch. Baumer, M. Novak), Wiesbaden: Harrassowitz.

Begmatov, A, Berdimurov A, Bogomolov G, Murakami T, Teramura H, Uno T, Usami T. 2020 New Discoveries from Kafir-Kala: Coins, Sealings and Wooden Carvings, in Sogdians. In Sogdiana*, China, and Turfan during the Sixth Century* (ed. Y. Yoshida), *Acta Asiatica* 119, 1–20.

Begmatov A. 2026 Cities on the North-Eastern Fringes of the Sasanian Empire and Beyond. In *Historical Aspects of Medieval to Modern Cities in West Asia* (ed T. Morikawa), Brepols. CUWANA 1, 111-126. (doi 10.1484/M.CUWANA-EB.5.151833)

Berdimuradov A, Bogomolov G, Begmatov A, Uno T. 2018. Novel Unique Finds of the Sogdian Art. *Sogdiyskiysbornik 5. Latest Research on the history and cultural history of Sogdiana in Uzbekistan,* 25-44 (In Russian).

Berdimurodov A, Teramura H, Uno T, Bogomolov G, Begmatov A, Sandiboyev A, Murakami T. 2023 Археологическиеработы в шахристане городища Кафиркала Узбекско-Японской экспедиции в 2022 г, Археологических исследований вУзбекистане 15, Самарканд (In Russian).

Berthon RJ-P, Mashkour M. 2008 Animal Remains from Tilbeşar Excavations, Southeast Anatolia, Turkey. *Anatolia antiqua. Eski Anadolu* 16, 23–51. (doi:[10.3406/anata.2008.1248](https://doi.org/10.3406/anata.2008.1248))

Biglari F, and Taheri K. 2000 The Discovery of Upper Palaeolithic Remains at Mar Kuliyan and Mar Dalan Cave, Rawansar. in *Essays on the Archaeology, Geology, Geography, and Culture of the Rawansar*, Kamal Taheri (ed.): 7-27. Kermanshah: Taq-e Bostan Publications (In Persian).

Bogomolov G, Berdimurodov A, Teramura H, Begmatov A, Sandiboev A. 2022. Gorodiŝe Kafirkala. Arheologičeskie raboty 2021 g.’ (The Kafir-kala Site. Archaeological Investigations of 2021). *Arheologičeskie issledovaniâ v Uzbekistane (Archaeological Research in Uzbekistan)* 14, 34–39 (in Russian).

Braidwood L., Braidwood, R. Howe B., Reed C., Watson P.J. (Eds.), Prehistoric Archaeology Along the Zagros Flanks. Studies in Ancient Oriental Civilization 105, University of Chicago Oriental Institute, Chicago (1983).

Broushaki F, Mashkour M, Abdi K, Davoudi H, Biglari F, Burger J. 2016 Human genome extraction from early Neolithic of Tappeh Abdol Hossein and Wezmeh Cave in Western Iran. In *Proceedings of 15th Annual Symposium on Iranian Archaeology*, H. Chubak (ed.), Tehran: Research Institute of Cultural Heritage and Tourism (RICHT): 791-793.

Carter E. 1992 A history of excavation at Susa: personalities and Archaeological methodologies. In: The royal city of Susa: ancient near eastern treasures in the Louvre, P. Harper, J. Aruz, F. Tallon (eds.), New York: The Metropolitan museum of Art: 20-25.

Daly KG, Mattiangeli V, Hare AJ, Davoudi H, Fathi H, Beizaee Doost S, Amiri S, Khazaeli R, Decruyeare D, Nokandeh J, Richter T, Darabi H, Mortensen P, Pantos A, Yeomans L, Bangsgaard P, Mashkour M, Zeder MA, Bradley DG. 2021 Herded and hunted goat genomes from the dawn of domestication in the Zagros Mountains. *PNAS (Proceedings of the National Academy of Sciences of the United States of America)* 118(25): e2100901118.

Danti MD. 2013 *Hasanlu V: The Late Bronze and Iron I Periods*. University of Pennsylvania Press.

Davoudi H. 2017 *Subsistence Economy of Iron Age Societies in North-Western Iran Based on Archaeozoological Studies-the Case of Tepe Hasanlu*. PhD thesis, Department of Archaeology, Faculty of Humanities, Tarbiat Modares University, Iran (in Persian).

Davoudi H, Mostafapour I, Zifar H, Moghimi M. 2016 Rescue excavation at archaeological site of Kholah Kouh, Takestan city, Qazvin province, summer 2014. In: *Proceedings of the 14th Annual Symposium on the Iranian Archaeology*, Tehran: RICHT: 179-184 (in Persian).

Davoudi H, & Mashkour M. 2017 *Faunal Remains from Tepe Dava Göz (West Azerbaijan, Iran): A Preliminary Report.* Tehran: Archive of the Bioarchaeology Laboratory of the University of Tehran (unpublished report).

Davoudi H, Berthon R, Mohaseb A, Sheikhi S, Abedi A, & Mashkour M. 2018 Kura-Araxes Exploitation of Animal Resources in Northwestern Iran and Nakhchivan. In C. Çakırlar, J. Chahoud, R. Berthon, & P. Birch (Eds.), *Archaeozoology of the Near East XII* (pp. 91–108). University of Groningen.

Davoudi H, & Mashkour M. 2019 Subsistence Economy in Northwestern Iran during Bronze and Iron Ages through Archaeozoological Researches at Tepe Hasanlu. In Y. Hassanzadeh, A. A. Vahdati, & Z. Karimi (eds.), *Proceedings of the International Conference on The Iron Age in Western Iran and Neighbouring Regions, Kurdistan University, Sanandaj, Iran, 2-3 November 2019* (Vol. 1, pp. 484–520). RICHT / National Museum of Iran and Kurdistan Province / ICHHTO (in Persian).

Davoudi H, Mashkour M, Abedi A, Maziar A, Rahimi R, Fathi H, Sheikhi S, Mohaseb AF, Berthon R. 2019a Herding and hunting in north-western Iran and adjacent areas from Late Neolithic to Early Bronze Age. In: *The Araxes River in Late Prehistory Bridge or Border?*, 14-15 May 2019, International Conference, Lyon, Collegium Lyon, France.

Davoudi H, Moghimi N, Sadeghi E. 2019b Delimitation sounding to identify core area and buffer zone of Tappeh Zāgheh of Abyek, Qazvin Province., *Proceedings of the 16th Annual Symposium on the Iranian Archaeology*, Tehran, RICHT: 185-190 (in Persian).

Davoudi H, Moghimi N, Sadeghi E. 2019c Excavation at ancient site of Ālou, Radekan Village, Takestan County. In: R. Shirazi (ed.), *Proceedings of the 16th Annual Symposium on the Iranian Archaeology*, Tehran: RICHT: 168–170 (in Persian).

Davoudi H, & Mashkour M. 2020 *Faunal Remains from Chahar Bagh Street (Isfahan, Iran): A Preliminary Report*. Archive of the Bioarchaeology Laboratory of the University of Tehran (unpublished report).

Davoudi H, Moghimi N, Shirazi Z. 2021 Cultural and Economic Transformations of the First Half of the Fifth Millennium B.C. in the Qazvin Plain, Based on the Results of Salvage Excavations at Kholah Kouh of Takestan. *Pazhohesh-ha-ye Bastanshenasi Iran* 10 (27): 29-54.

Davoudi H, Fathi H, Mashkour M. 2026 *Faunal Remains from Deh Dumen Cemetery (Kohgiluyeh and Boyer-Ahmad, Iran)*: *A Preliminary Report.* Archive of the Bioarchaeology Laboratory of the University of Tehran (unpublished report).

de Mecquenem R. 1934 Fouilles de Suse 1929-1933. *Memoires de la Delegation en Perse* XXV: 25: 177-237.

Decruyenaere D, Rante R, Vuillien M, Mashkour M. 2024 Chapter 10. Animal remains of the Oasis of Bukhara: preliminary results. in *The Oasis of Bukhara, Vol. III*, Material Culture, Socio-territorial Features, Archaeozoology and Archaeometry(eds R. Rante, J. Mirzaakhmedov), Louvre Edition, Brill, Leiden/Boston, 364-370.

Dehpahlavan M, Alinezhad Z. 2022 The Cylinder Seals of Qareh Tepe in Sagzabad, Iron Age II and III. *Iran* 60: 1-15.

Dickens M. 2017 Syriac Inscriptions Near Urgut, Uzbekistan. *Studia Iranica* 46, 205- 260.

Didier A. 2017 Nouvelles recherches sur les débuts de la civilisation de l’Indus au Pakistan. Les fouilles de Chanhu-Daro (Sindh). *Comptes rendus des séances de l'Académie des Inscriptions et Belles-Lettres* 161(2): 969-1002.

Didier A, Castillo DS, Mongne P, & Shah SSA. 2017 Resuming excavations at Chanhu-daro, Sindh: First results of the 2015-2017 field-seasons. *Pakistan Archaeology* 30: 69-121.

Didier A, Marchand F, Sarmiento-Castillo D. 2024 Les lapidaires de Chanhu-daro (Sindh, Pakistan). Logiques productives et organisation socio-économique dans un centre urbain majeur de l’Âge du bronze en Asie du Sud. C. Cheval, O. Langlois, M. Lauwers, G. Palumbi, H. Procopiou (eds.)*, Acteurs techniques, acteurs sociaux. Des vestiges matériels à l’organisation sociale du travail, de la Préhistoire à nos jours*. 43es rencontres internationales d’archéologie et d’histoire – Nice Côte d’Azur, Éditions APDCA: 89-102.

Didier A. In press Chanhu-daro, a Major Urban Planned City for Better Understanding the Internal Development of the Indus Valley Civilization. *Sindh Antiquities*, [⟨halshs-04365840⟩](https://shs.hal.science/halshs-04365840v1)

Dyson RH, & Muscarella OW. 1989 Constructing the Chronology and Historical Implications of Hasanlu IV. *Iran* 27(1): 1–27.

Fages A, Hanghøj K, Khan N, Gaunitz C, Seguin-Orlando A, Leonardi M, Constantz CM, Gamba C, Al-Rasheid KA, Albizuri S, Alfarhan AH, et al. 2019 Tracking five millennia of horse management with extensive ancient genome time series. *Cell* *177*(6): 1419-1435. [10.1016/j.cell.2019.03.049](https://doi.org/10.1016/j.cell.2019.03.049)

Fazeli Nashli H, Coningham R, Marghussian A, Manuel M, Azizi Kharanaghi, MH, & Pollard M. 2013 Mapping the Neolithic occupation of the Kashan, Tehran and Qazvin plains. In *The Neolithsation of Iran, The Formation of New Societies*, R. Matthews and H. Fazeli Nashli (eds.), 114-135, British Association for Near Eastern Archaeology (BANEA), Oxford: Oxbow Books.

Fiddaman SR, Dimopoulos EA, Lebrasseur O, Du Plessis L, Vrancken B, Charlton S, Haruda AF, Tabbada K, Flammer PG, Dascalu S, and Marković N. 2023 Ancient chicken remains reveal the origins of virulence in Marek’s disease virus. *Science* *382*(6676):1276-1281. [DOI: 10.1126/science.adg2238](https://doi.org/10.1126/science.adg2238).

*Field Report on the Archaeological Excavations at Urgut-Suleiman-Tepa, Uzbekistan in 2022*. International Institute for Central Asian Studies, Ya. Gulyamov Samarkand Archaeological Institute, Society for the Exploration of Eurasia. IICAS: Samarkand. https://www.exploration-eurasia.com

*Field Report on the Archaeological Excavations at Urgut-Suleiman-Tepa, Uzbekistan in 2023*. International Institute for Central Asian Studies, Ya. Gulyamov Samarkand Archaeological Institute, Society for the Exploration of Eurasia. IICAS: Samarkand. https://www.exploration-eurasia.com

*Field Report on the Archaeological Excavations at Urgut-Suleiman-Tepa, Uzbekistan in 2024*. International Institute for Central Asian Studies, Ya. Gulyamov Samarkand Archaeological Institute, Society for the Exploration of Eurasia. IICAS: Samarkand. https://www.exploration-eurasia.com

Fouache E, Rante R, Mirzaakhmedov J, Ragala R, Dupays M, et al. 2016 The Role of Catastrophic Floods Generated by Collapse of Natural Dams Since the Neolithic in the Oases of Bukhara and Qaraqöl: Preliminary Results. *International Journal of Geohazards and Environment* 2(3), 150-165.

Fouache E, Cez L, Andrieu-Ponel V, Rante R. 2020 Environmental changes in Bactria and Sogdiana (Central Asia, Afghanistan and Uzbekistan) from the Neolithic to the Late Bronze Age: Interaction with the human occupation. In *The World of the Oxus Civilization* (eds B. Lyonnet & NA. Dubova), Routledge Worlds, 82-109.

Gagoshidze I, Rova E. 2016. 2013–2015 Activities of the Georgian-Italian Shida Kartli Archaeological Project at Aradetis Orgora (Georgia). In *Proceedings of the 10th International Congress on the Archaeology of the Ancient Near East: 25–29 April 2016, Vienna* (ed. B. Horejs), Wiesbaden, 497–510.

Gagoshidze I, Gobejishvili N, Gagoshidze D, Rova E, Bianchi F, Bertoldi F, Kvavdze E, Rasia P A. 2025 Collaborative Research on the Doghlauri Cemetery, in *Proceedings of the 13th International Congress on the Archaeology of the Ancient Near East, Copenhagen, 22–26 May 2023. Volume 2: Field and Lab Reports* (eds. S. Haddow, C. Mazzucato, I. Thuesen),Wiesbaden, Harrassowitz, 99-114.

Galdieri E. 1972 *Isfahān: Masğid-i Ğuma*. Vol. I, Roma: IsMEO.

Galdieri E. 1973 *Isfahān: Masğid-i Ğuma*. Vol. II, Roma: IsMEO.

Galdieri E. 1984 *Isfahān: Masğid-i Ğuma*. Vol. III, Roma: IsMEO.

Gambashidze I. 2021 Radiocarbon Dates from the Orchosani Settlement and the Chalcolithic/Early Bronze Age Chronology of Samtskhe (South-West Georgia). In: L. Giemsch and S. Hansen (eds). *The Caucasus, bridges between the urban centres in Mesopotamia and the Pontic steppes in the 4th and 3rd millenium BC. The transfer of knowledge and 229 technologies between East and West in the Bronze Age*. Schriften des Archäologischen Museums Frankfurt, Regensburg: Verlag Schnell & Steiner GmbH: 133–158.

Gangler A, Gaube H, Petruccioli A. 2004 Bukhara – The Eastern Dome of Islam: Urban Development, Urban Space, Architecture and Population. London.

Gasche H. 2009 SUSA i. EXCAVATIONS. *Encyclopaedia Iranica*.

Genito B. 1990 The Most Frequent Pottery Types at Dahan-i Gholaman (Sistan) and Their Spatial Variability. *South Asian Archaeology 1987*. Proceedings of the ninth international conference of the association of South-Asian Archaeologists in Western Europe, held in the fondazione Giorgio Cini, island of San Giorgio Maggiore, Venice. 2 vols. Rome: Istituto Italiano per il middle and far east, 1990. (Italian institute for the middle and far east, Serie Orientale Roma 66/1-2): 588-601.

Genito B, Saiedi-Anaraki F. (eds.). 2011 *ADAMJI Project From the Excavation (1972-1978) to the Archive (2033-2010) in the Masjed-e Jom’e Isfahan*. ICHTO, Embassy of ItalyTehran, IsIAO, Tehran.

Ghasemi P, and Watson G. 2014 The Khersan 3 Archaeological Survey: New Insights into Settlement Patterns in the Zagros Folding Zone from Hydrodam Project Archaeology. *Ancient Near Eastern Studies* 51:169–211. <https://doi.org/10.2143/ANES.51.0.3038718>.

Ghirshman R. 1939 *Fouilles de Sialk, près de Kashan, 1933, 1934, 1937*. Vols. 1–2, Librairie Orientaliste Paul Geuthner: Paris.

Ghirshman R. 1963 *Compte-rendu des séances de l’académie des inscriptions et elles-lettres, Fouille de Suse dans l’hiver 1962-63*: 187-196.

Gilbert S T, Voyakin D, Sorokin D. In press The Monks of Urgut: Discovery of a Monastic Cemetery in Uzbekistan. *The Syriac Christian Heritage in Central Asia and China: Continuities and Transformations* (Eds L. Tang, D. Winkler), Berlin: Lit Verlag.

Gilbert S T. 2024 Die Missionarsmönche Der Seidenstrasse: Wie die Archäologie eine verloren geglaubte christliche Geschichte in Zentralasien enthüllt. *Antike Welt* 5(24), 55-65.

Gintzburger G, Toderich K N, Mardonov B K, Mahmudov M M. 2003 *Rangelands of the arid and semi-arid zones in Uzbekistan*. CIRAD - ICARDA.

Gnoli G. 1993 Dahan-e Ḡolāmān. *Encyclopaedia Iranica*, Vol. VI, Fasc. 6: 582-585. <https://doi.org/10.1163/9789004693999>

Grenet F. 2020 The wooden panels from Kafir-kala: a group portrait of the Samarkand nāf (civic body). *Acta Asiatica: Bulletin of the Institute of Eastern Culture* 119, 21-42.

Gulyamova L. 2022. *The Geography of Uzbekistan, at the Crossroad of the Silk Road*. World Regional Geography Book Series, Springer. (doi.org/10.1007/978-3-031-07873-6)

Itahashi et al, 2024. Review of the PPN-PN Transition at the North Mesopotamia based on sequential radiocarbon dating of sediments. Poster presentation at the World Neolithic Congress, 4-8 Nov 2024- Sanliurfa, Türkiye.

Javeri M. 2021 Salvage Excavation in the Southern Part of . In: *Proceedings of 18th Annual Symposium on the Iranian Archaeology 2021*, R. Shirazi (ed.), Tehran: RICHT: 157-168 (in Persian).

Jebreili Y, Rafiei-Alavi B, Shojaee-Esfahani A. (in press) The Archaeological Landscape of the Western Gavkhoni Plains, the Study of the Pattern and Settlement System of the Site the Early Bronze Age II. *Documenta Praehistorica*.

Kakavand M, Rafiei-Alavi B, Emami MA, Kehl M. 2025 Evidence of Early Bronze Age Metallurgy from the Ancient Sites of Varzaneh Plain (Isfahan), Considering Mineralogical, Chemical, and Provenance Studies. *Metalla* 29(1): 3-32. DOI:10.46586/metalla.v29.2025.i1.3-32.

Kamjan S, Mashkour M, Mohaseb FA, Fathi H, Azizi Kharanaghi MH. 2018 Zooarchaeology of the Pre-Pottery and Pottery Neolithic site of Qasr-e Ahmad (Iran). In: C. Cakirlar, J. Chahoud, R. Berthon, S. Pilaar Birch (eds.), *Archaeozoology of the Near East XII, Proceeding of 12^th^ International Symposium of the ICAZ Archaeozoology of Southwest Asia and Adjacent Areas Working Group (ASWA)*, Groningen Institute of Archaeology, 14-15^th^ June 2015, University of Groningen, the Netherlands, Chapter 3: 27-40.

Kavosh HA, Vidale M, Fazeli Nashli H. (eds.). 2019 *Tappe Graziani, Sistan, Iran: stratigraphy, formation processes and chronology of a suburban site of Shahr-i Sokhta*. Prehistoric Sistan 2, ISMEO, Roma.

Kavosh H, & Oveisi-Keikha Z. 2024 Manufacturing architecture, evidence of pottery production from Tappeh Graziani, Sistan, Iran. *Historia I Świat* 13: 55–70. https://doi.org/10.34739/his.2024.13.04

Khazaeli R, Mashkour M, Mohaseb F, & Others. 2021 An Introduction to Archaeozoological Studies of Nishapur in the Islamic Period up to the 7th H./13th CE Century. *Pazhoheshha-Ye Bastan Shenasi Iran*, *11*(28), 111–133.

Khazaeli R, & Mashkour M. 2025 *Faunal Remains from Kākh-e Jahānnamā (Isfahan, Iran): A Preliminary Report*. Archive of the Bioarchaeology Laboratory of the University of Tehran (unpublished report).

Khojimatov O K, Bussmann R W, Gafforov Y. 2023 Uzbekistan – ecosystems, biodiversity, history and culture. in *Ethnobiology of Uzbekistan. Ethnobiology* (eds. O K Khojimatov, Y Gafforov & R W Bussmann ), Springer. (doi:10.1007/978-3-031-23031-8_1)

Laleh H, Mokarramifar A, and Lorzadeh Z. 2015 Le paysage urbain de Nishāpur. In *Greater Khorasan: History, Geography, Archaeology and Material Culture*, Rocco Rante (ed.), 115-124. Studies in the History and Culture of the Middle East Vol. 29. Berlin, München, Boston: De Gruyter.

Laleh H, and Lorzadeh Z. 2019 Archaeological Research in the Nishāpur Urban Historic Landscape (First Season). *Proceedings of the 17th Annual Symposium on the Iranian Archaeology 2018-2019*, pp. 1087–1096. Tehran: Research Center for Cultural Heritage and Tourism Publications (in Persian).

Le Breton L.1957 The Early Preiods at Susa, Mesopotamian Relatiosns. *Iraq* 19: 79-124.

Le Brun A. 1978 Suse, chantier ‘Acropole 1’. *Paleorient* 4: 177-192.

Lorzadeh Z, and Laleh H. 2018 Agricultural Landscape of Nishāpur and the Hinterland of a Metropole. *Journal of Islamic Archaeology* 5.1: 3-16.

Malek Shahmirzadi S. (ed.) 2003 *The silversmiths of Sialk*. “Sialk Reconsideration Project Monograph” 2, Iranian Center for Archaeological Research: Tehran (in Persian).

Malek Shahmirzadi S. (ed.) 2004 *The potters of Sialk*. “Sialk Reconsideration Project Monograph” 3, Iranian Center for Archaeological Research: Tehran (in Persian).

Malek Shahmirzadi S. (ed.) 2006 *Sialk, the oldest fortified village of Iran*. Iranian Center for Archaeological Research: Tehran (in Persian).

Mantellini S, Berdimurodov A. 2005 Archaeological Explorations in the Sogdian Fortress of Kafir Kala (Samarkand Region, Republic of Uzbekistan). *Ancient Civilizations from Scythia to Siberia* 11, 107-32.

Mantellini S. 2017 A city and its landscape across time: Samarkand in the ancient Sogdiana (Uzbekistan). *Archeologia e Calcolatori* (eds S. Garagnani, A. Gaucci), Proceedings of the KAINUA 2017 International Conference in Honour of Professor Giuseppe Sassatelli’s 70th Birthday (Bologna, 18-21 April 2017), 28.2, 333-342.

Mantellini S. 2018 Landscape Archaeology and Irrigation Systems in Central Asia: A View from Samarkand (Uzbekistan). In *Urbanized Landscapes in Early Syro-Mesopotamia and Prehispanic Mesoamerica* (eds D. Domenoci, N. Marchetti), Papers of a Cross-Cultural Seminar held in Honor of Robert McCormick Adams, Harrassowitz Verlag · Wiesbaden, 169-204.

Mariani L. 1977 Conservation Work on Building 3 at Dahan-e Ghulaman, Sistan. *South Asian Archaeology* 2: 737-754.

Marro C. 2022 The View from the North. *Paléorient* 48(1): 111-130. DOI: 10.4000/paleorient.1675.

Martin Y. 2023 *Etude archéozoologique d’une faune iranienne au Muséum National d’Histoire Naturell*e. Rapport de stage de deuxième année de cycle ingénieur AgroParisTech. UMR 7209 BioArch Laboratory MNHN/CNRS, Paris, France.

Mashkour M. 2001 *Chasse et élevage du Néolithique à l’Age du Fer dans la plaine de Qazvin (Iran). Etude archéozoologique des sites de Zagheh, Qabrestan et Sagzabad*. Thèse de Doctorat de l’Université de Paris I-Panthéon-Sorbonne, Paris.

Mashkour M. 2001 *Faunal Remains from Kulian Cave (Kermanshah, Iran): A Preliminary Report.* Tehran: Archive of National Museum of Iran (unpublished report).

Mashkour M. 2002 An introduction to the Archaeozoology of Sialk: A Preliminary report of the faunal remains from the first research campaign at Tepe Sialk of Kashan. In: Malek Shahmirzadi (ed.), *The Ziggurat of Sialk, Report N°1*, ICHTO: Pp.135-142. (in Persian).

Mashkour M. 2025 *Faunal Remains from Susa Complex (Khuzestan, Iran): A Preliminary Report*. Archive of the UMR 7209 CNRS/MNHN, Paris (unpublished report).

Mashkour M, Fontugne M, & Hatte C. 1999 Investigation on the evolution of subsistence economy in the Qazvin Plain (Iran) from the Neolithic to the Bronze Age. *Antiquity* 73: 65-76.

Mashkour M, Biglari F, Ghafouri N, Ahadi G, Amiri S, Beizaei Doust S, Davoudi H, Fathi H, Khazaeli R, Moradi B, Yashmi R. 2012 The Osteoarchaeological Project of the National Museum of Iran: An Interim Report. *Iranian Archaeology* 3: 72-76. https://sites.google.com/site/iranianarchaeology.

Mashkour M, & Mohaseb FA. 2012 *Preliminary report of the faunal remains of the Zilou Cave, Perau mountains, Northern Kermanshah*. Tehran: Archive of National Museum of Iran (unpublished report).

Mashkour M, Fathi H. 2015 *Faunal Remains from Tepe Forudgah (Lorestan, Iran): A Preliminary Report.* Tehran: Archive of the Bioarchaeology Laboratory of the University of Tehran (unpublished report).

Mashkour M, Fathi H, & Davoudi H. 2015 *Faunal Remains from Soha Chay Tepe (Zanjan, Iran): A Preliminary Report.* Tehran: Archive of the Bioarchaeology Laboratory of the University of Tehran (unpublished report).

Mashkour M, & Fathi H. 2016 *Faunal Remains from Dahan-e Gholaman Ancient City (Sistan and Baluchestan, Iran): A Preliminary Report.* Tehran: Archive of National Museum of Iran (unpublished report).

Mashkour M, Fathi H, Amiri S, & Davoudi H. 2016 *Faunal Remains from Tepe Chalow (North Khorasan, Iran): A Preliminary Report.* Tehran: Archive of the Bioarchaeology Laboratory of the University of Tehran (unpublished report).

Mashkour, M., Davoudi, H., Fathi, H., Amiri, S., Khazaeli, R., Beizaee Doost, S. 2018 *The Study of Faunal Remains Abdul Hosein Housed at the Osteology Department of the National Museum of Iran. A Neolithic Site in Central Zagros. 2018*. Tehran: Archive of National Museum of Iran (unpublished report).

Mashkour M, Fathi H, Davoudi H, Kavosh HA. 2019 Animals remains from Tappeh Graziani. In: HA Kavosh, M Vidale, H Fazeli Nashli (eds.), *Tappe Graziani, Sistan, Iran: stratigraphy, formation processes and chronology of a suburban site of Shahr-i Sokhta*, Prehistoric Sistan 2, ISMEO, Roma: 173-186.

Mashkour M, Fathi H. 2020 *Faunal Remains from Ali Tappeh Cave (Mazandaran, Iran): A Preliminary Report.* Tehran: Archive of National Museum of Iran (unpublished report).

Mashkour M, Fathi H, Davoudi H. 2020 Interaktionen zwischen Mensch und Tier im Salzbergwerk Douzlākh, Chehrābād. In: T. Stöllner, A. Aali and N. Bagherpour Kashani (eds.), [*Tod im Salz. Eine archäologische* *Ermittlung* *in Persien*. Begleitbuch, Katalog und Graphic Novel,](http://paperpile.com/b/MLp2xa/1lxf) Germany, Bochum: Deutsches Bergbau-Museum Bochum: 219-224.

Mashkour M, Amiri S, Fathi H, Khazaeli R, Debue K, Decruyenaere D, Beizaee Doost S, Clavel B, Kamjan S, Jajanidze R, and Sauer EW. 2020 Herding and Hunting in the highlands from the Sasanian to Late Medieval Periods. The Archaeozoology of the Dariali Gorge. In E.W. Sauer Ed. Dariali: The 'Caspian Gates' in the Caucasus from Antiquity to the Age of the Huns and the Middle Ages: The Joint Georgian-British Dariali Gorge Excavations and Surveys 2013-2016. Oxbow Books. Series: British Institute of Persian Studies Archaeological Monograph Series | Volume: 6, Pp. 729-788 + 174 p. Appendices.

Mashkour, M., Davoudi, H., Mohaseb, F. A., Beizaee Doost, S., Khazaeli, R., Amiri, S., Fathi, H. 2021. *Human and Animal Interactions in the Iranian Plateau: Research Conducted by the Osteology Department of Iran National Museum*. Bibliothèque iranienne 85, Tehran: Iran National Museum Publications and Institut Français de Recherche en Iran.

Mashkour M, Davoudi H, Mohaseb FA, Beizaee Doost S, Khazaeli R, Amiri S, Fathi H. 2021 *Human and Animal Interactions in the Iranian Plateau: Research Conducted by the Osteology Department of Iran National Museum*. Iran National Museum publications and Institut Français de Recherche en Iran. Musée National d’Iran. See <https://cnrs.hal.science/hal-03850516>.

Mashkour M, Laleh H, Shojaee Esfahani A, Fathi H, Khazaeli R, Fathi Z, Lorzadeh Z, Haji Alilou S, Kargar A. 2024a *Feeding Cities: A millennium of food traditions in Isfahan viewed from the faunal remains of the Great Mosque*. Final Scientific Report, Barakat Trust.

Mashkour M, Laleh H, Shojaee Esfahani A, Lorzadeh Z, Fathi H, Davoudi H, Khazaeli R, Fathi Z, Haji Alilou S, Kargar A, Allahyari H, Djamali M. 2024b Feeding Cities: A millennium of food traditions in Isfahan viewed from the faunal remains of the Great (Friday) Mosque. In: *Second ICAZ Medieval Period Working Group Meeting*, 2-6 October 2024, Sofia History Museum, Sofia, Bulgaria.

Mashkour M, Fathi H, Beizaee Doost S. 2025 *Faunal Remains from Varzaneh 013 and 051 Sites (Isfahan, Iran)*: *A Preliminary Report.* Archive of the Bioarchaeology Laboratory of the University of Tehran (unpublished report).

Mashkour M, Fathi H, Davoudi H. 2025 *Faunal Remains from Tepe Naderi (North Khorasan, Iran): A Preliminary Report*. Archive of the Bioarchaeology Laboratory of the University of Tehran (unpublished report).

Mashkour M., Tengberg M., Didier A. Sarmiento Castillo D. 2025 From Bioarcheological Research to Capacity Building in the Indus Basin. *10 YEARS OF PAKISTANI FRENCH ARCHAEOLOGICAL EXCAVATIONS AT CHANHU DARO, SINDH*, Ambassade de France au Pakistan; Taxila Institute of Archaeology and Civilizations; Mission Archéologique Française du Bassin de l'Indus, Oct 2025, Islamabad, Pakistan. [⟨hal-05430719⟩](https://hal.science/hal-05430719v1)

McBurney CBM, and Rosemary P. 1969 The Cave of Ali Tappeh and the Epi-Palaeolithic in NE Iran. In *Proceedings of the Prehistoric Society* 34: 385-413. <https://doi.org/10.1017/S0079497X00013955>.

Moghimi N. 2018 *Re-identifying the Socioeconomic Organizations of Human Societies in Tākestān Region during the Fifth and Fourth Millennia B.C.* PhD thesis, Department of Archaeology, Faculty of Literature and Humanities, University of Tehran (in Persian).

Moghimi N, Davoudi H. 2020 Evidence of Administrative Technology during the Fourth Millennium BC in the Qazvin Plain, Iran. *Journal of Archaeological Studies* 12(1): 221–241.

Muhamedzhanov A, Mirzaakhmedov J, Adylov Sh. 1982 Keramika nizhnikh sloev Bukhary (Opyt predvaritel’noj periodizatsii) (Ceramics of the Lower Layers of Bukhara (Experience of Preliminary Periodization)). *IMKU* 17, 81-97.

Muhamedzhanov A R, Adylov Sh T, Mirzaakhmedov J, Semenov G L. 1988 Gorodishche Pajkend. K probleme izuchenija srednevekovogo goroda Srednej Azii (Paykend Settlement. On the Problem of Studying a Medieval City of Central Asia). FAN, Tashkent.

Murakami T, Begmatov A, Teramura H, Uno T, Usami T, Berdimurodov A, Bogomolov G. 2021 Summary of Excavations at Kafir-kala in Uzbekistan up to 2020: Cultural Exchanges as Reflected in the Novel Discoveries. *Ancient Orient through Excavations in 2020, Proceedings of the 28th Annual Meeting of Excavations in West Asia. Japanese Society for West Asian Archaeology*, 80-85 (In Japanese).

Murakami T, Begmatov A, Teramura H, Berdimurodov A, Bogomolov G, Sandiboev A, Uno T, Usami T. 2022 Excavations at Kafir-kala in Uzbekistan in 2021 - The Residential Area (Shahristan) of the Sogdian Royal Residence. *Ancient Orient Revealed through Excavations in 2021, Proceedings of the 29th Annual Meeting of Excavations in West Asia . Japanese Society for West Asian Archaeology*, 18- 22 (In Japanese).

Murakami T, Begmatov A, Sandiboev A, Hujamov S, Mahamadiev G, Arsiev K, Uno T, Teramura H. 2023c Excavations at Kurgon-Tepa in Uzbekistan (2023). Investigating of Cities in Sogdiana.. *Proceedings of the 31st Annual Meeting of Excavations in West Asia. Japanese Society for West Asian Archaeology* 31, 150–154. (in Japanese).

Murakami T, Begmatov A, Sandiboev A, Mahamadiev G, Alimov N, Teramura H, Uno T. 2024 Exploring Urban Centers in Sogdiana: Excavations at Kurgon-Tepa in Uzbekistan (2023). *Proceedings of the 31st Annual Meeting of Excavations in West Asia. Japanese Society for West Asian Archaeology* 31, 150–154. (in Japanese).

Murakami T, Watanabe Y, Begmatov A, Berdimurodov A, Bogomolov G, Sandiboev A, Teramura H, Uno T. 2023a Timber and Flora from Kafir-kala in Samarkand: Unveiling Species and Practical Applications, *Archaeology of Uzbekistan* 2/29, 74-94. ([doi.org/10.71414/2181-032X-2023-2-29-74-94](http://doi.org/10.71414/2181-032X-2023-2-29-74-94))

Murakami T, Teramura H, Uno T, Begmatov A, Berdimurodov A, Bogomolov G, Sandiboev A. 2023b Excavations at Kafir-kala in Uzbekistan in 2022 – The Residential Area (Shahristan) of the Sogdian Royal Residence. *Ancient Orient Revealed through Excavations in 2022, Proceedings of the 30th Annual Meeting of Excavations in West Asia. Japanese Society for West Asian Archaeology*, 98–102 (In Japanese).

Murakami T, Begmatov A, Sandiboev A, Mahamadiev G, Berdimurodov A, Reutova M, Alimov N, Subkhonov F, Teramura H, Uno T, Suemori K, Oshikane H. 2025. Exploring Urban Centres in Sogdiana: Excavations at Kurgon-Tepa in Uzbekistan (2024). *Proceedings of the 32nd Annual Meeting of Excavations in West Asia. Japanese Society for West Asian Archaeology* 32, 111-115 (in Japanese).

Muscarella OW. 2006 The Excavation of Hasanlu: An Archaeological Evaluation. *Bulletin of the American Schools of Oriental Research. American Schools of Oriental Research*, *342*, 69–94.

Naseri R, and Khanipour M. 2025 Burial Practices of the Bronze Age, An Archaeological Study of the Deh Dumen Cemetery in Southern Zagros, Iran. *Near Eastern Archaeology* 88(4): 260-271.

Naymark A. 2003 Returning to Varaksha. *The Silk Road Newsletter* 1/2, available online: http://www.silk-road.com/newsletter/december/varakhsha.htm.

Negahban EO. 1976 Preliminary Report on the Excavation of Sagzabad. in *Memorial Volume of the Vth International Congress of Iranian Art and Archaeology, Oxford, 1972*, 247-72.

Negahban EO. 1977 “Preliminary Report of Qazvin Expedition: Excavation of Zagheh, Ghabristan, Sagzabad”. *Marlik*, Vol. II: 26-44.

Nekrasova EG. 1999 La citadelle de Bukhara de la fin du 9e siècle au début du 13e siècle. *Archéologie Islamique* 8-9, 37-54.

Nokandeh J. 2010 *Neue Untersuchungen zur Sialk III-Periode im zentraliranischen Hochland: auf der Grundlage der Ergebnisse des, Sialk Reconsideration Project*. dissertation. de-Verlag im Internet GmbH, Berlin.

Omelʹchenko A V, Mirzaakhmedov J. 2024 Chapter 3. The Bukhara Archaeological Expedition: The History of Research in Paykend. In The Oasis of Bukhara, Volume 3, Material Culture, Socio-territorial Features, Archaeozoology and Archaeometry (ed R. Rante), Brill, Leiden, 43-93.

Oudbashi O, Naseri R, Cultrone G, Egartner I, Arizzi A. 2021 The Pottery Production from the Deh Dumen Bronze Age Graveyard (South-western Iran): A Chemical, Mineralogical and Physical Study. *Heritage Science* 9: 83. https://doi. org/10.1186/s40494-021-00557-6.

Perrot J. 1978 *La séquence archéologique de Suse et du Sud-Ouest de l'Iran antérieurement à la période achéménide*. Actes de la Rencontre internationale de Suse, Iran, du 23 au 28 octobre 1977. Organisée par la Délégation archéologique française en Iran et par le Centre iranien de recherche archéologique. Paris: CNRS.

Perrot J. 2010 *Le palais de Darius à Suse: une résidence royale sur la route de Persépolis à Babylone*. Paris: [PUPS, Presses de l'université Paris-Sorbonne](https://www.google.de/search?hl=en&gbpv=1&bsq=%E2%80%A2+Jean+Perrot,+Le+palais+de+Darius+%C3%A0+Suse+:+la+grande+r%C3%A9sidence+royale+de+la+Perse+ach%C3%A9m%C3%A9nide&dq=%E2%80%A2+Jean+Perrot,+Le+palais+de+Darius+%C3%A0+Suse+:+la+grande+r%C3%A9sidence+royale+de+la+Perse+ach%C3%A9m%C3%A9nide&printsec=frontcover&q=inpublisher:%22PUPS,+Presses+de+l%27universit%C3%A9+Paris-Sorbonne%22&tbm=bks&sa=X&ved=2ahUKEwjGkoyy9JSSAxVVB9sEHa_6FP0QmxN6BAgNEAI&sxsrf=ANbL-n6WWyx8W_J2zHNccZOivTdUOljCAg:1768733135828).

Portero R, Arino E, Elorza M, Gurt J-M, Martinez Ferreras V, Pidaev S R. 2022. Zooarchaeology of ancient Termez (Uzbekistan): From the Greco-Bactrian period to Islamic times. *Archaeological Research in Asia* 31, 100384. ([doi.org/10.1016/j.ara.2022.100384](http://doi.org/10.1016/j.ara.2022.100384))

Pullar J. 1990. *Tepe Abdul Hosein: A Neolithic Site in Western Iran Excavations 1978*. Oxford: British Archaeological Reports.

Puschnigg G, Bruno J. 2024. Chapter 4 Pottery. In *The Oasis of Bukhara, Volume 3, Material Culture, Socio-territorial Features, Archaeozoology and Archaeometry* (ed R. Rante), Brill, Leiden, 94-235.

Rafiei-Alavi B, Shojaee-Esfahani A. 2020 Short Report on the Excavation in two Sites at the Eastern Part of the Zāyandeh-Rud Basin, Kafarved (Kafrood)-Varzaneh Plain, Summer 2018. In: *Proceedings of 17th Annual Symposium on the Iranian Archaeology 2018-2019*, R. Shirazi and Sh. Hurshid (eds.), Tehran: RICHT, Vol. 2: 517-526 (In Persian).

Rafiei-Alavi B, Shojaee Isfahani A, Jebraili Y, and Allah-Yari H. 2021a Short Report on the Archaeological Excavation at ZR. MS. 051 site in the Eastern Part of the Zāyandeh-Rud River Basin. In: *Proceedings of 18th Annual Symposium on the Iranian Archaeology 2021*, R. Shirazi (ed.), Tehran: RICHT: 353-362 (in Persian).

Rafiei-Alavi B, Shojaee Isfahani A, Jebreili Y, and Kehl M. 2021b From Gavkhuni to Jazmurian: an investigating of interactions between plains in the lower part of the Zayandehrud basin and southeast of Iran in Bronze Age. In: R. Naseri, J. Alaei-Moghaddam and H. Sarhadi-Dadian (eds.), *Abstracts 3rd International Conference on the Archaeology of Southeastern Iran*. University of Zabol. p.39.

Rahimi Sorkhani, R. & M. Eslami. 2018. Specialized pottery production in Dalma tradition; a statistical approach in pottery analysis from Soha Chay Tepe, Zanjan, Iran. *Journal of Archaeological Science: Reports* 17: 220–34. <https://doi.org/10.1016/j.jasrep.2017.11.007>.

Rante R. 2021 Evolution of the habitat of Paykend », dans The History and Culture of Iran and Central Asia in the First Millennium CE: From the Pre-Islamic to the Islamic Era. in *The History and Culture of Iran and Central Asia in the First Millennium CE: From the Pre-Islamic to the Islamic Era* (eds D. Tor, M. Inaba), University of Notre-Dame Press De Gruyter, Berlin.

Rante R. 2024 The Oasis of Bukhara, Volume 3. Material Culture, Socio-territorial Features, Archaeozoology and Archaeometry. Serie: *Art and Archaeology of the Islamic world* 21, Leiden, The Netherlands: Brill.Rante R, Raimkulov A. 2013 Les fouilles de Paykend : nouveaux éléments. *Cahiers d’Asie centrale* 21/22, 237-258.

Rante R, Fouache E, Mirzaakhmedov J. 2016 Dynamics of human settlements ensuing from river transformation and changes in commercial behaviour: The birth of the “North-eastern Silk Road. *Journal of Archaeological Science Reports* 9, 437-447. (doi.org/10.1016/j.jasrep.2016.08.008)

Rante R, Mirzaakhmedov J. 2019 The Oasis of Bukhara, Volume 1. Population, Depopulation and Settlement Evolution. Serie: *Art and Archaeology of the Islamic world* 12, Leiden, The Netherlands: Brill. (doi.org/10.1163/9789004396258)

Rante R, Schwarz F, Tronca L. 2022 The Oasis of Bukhara, Volume 2. An Archaeological, Sociological and Historical Study. Serie: *Art and Archaeology of the Islamic world* 17, Leiden, The Netherlands: Brill. (doi.org/10.1163/9789004513662)

Rasia P. A., Bertoldi F., Rova E., Bitadze L. 2021. The Bronze Age burials from Doghlauri (Georgia). Preliminary analysis of human remains reveals a change in burial custom, *Journal of Archaelogical Science Reports* 38, 103048.

Renette S. 2022 Defining Dalma: an Incipient Mountain Identity?. *Paléorient* 48(1): 111-130.

Renette S, Mohammadi Ghasrian S. 2020 The central and northern Zagros during the Late Chalcolithic: An updated ceramic chronology based on recent fieldwork results in western Iran. Paléorient 46(1): 109-132.

Richardin P, Rante R. 2024. Chapter 11 Radiocarbon dating in the oasis of Bukhara. In *The Oasis of Bukhara, Volume 3, Material Culture, Socio-territorial Features, Archaeozoology and Archaeometry* (ed R. Rante), Brill, Leiden, 371-381.

Rouse L M, Mirzaakhmedov J, Stark S, Mirzaakhmedov S, Rakhmanov H, Ammerman S, Bechter A, Bayani N, Erb-Satullo N L, Haruda A F, Ho J W I, Hubbard E T, Hunter S A, Luneau E, Pourmomeni S, Schreiber F, Silvia Z W, Specia G. 2026 Archaeological investigations at the late 2nd millennium BCE site of Kimirek-Kum 1, Uzbekistan: Fieldwork seasons in spring and autumn 2024 (third preliminary report). *Archaeological Research in Asia* 45: 100666. (doi.org/10.1016/j.ara.2025.100666)

Rova E, Puturidze M, Makharadze Z. 2010 The Georgian-Italian Shida Kartli Archaeological Project: a report on the first two field seasons 2009 and 2010. *Rivista di Archaeologia*, 5-42.

Rova E, Makharadze Z, Puturidze M. 2017 Khashuri Natsargora: New Research about the Kura-Araxes and Bedeni Cultures in Central Georgia, in E. Rova, M. Tonussi (eds.), *At the Northern Frontier of Near Eastern Archaeology: Recent Research on Caucasia and Anatolia in the Bronze Age / An der Nordgrenze der vorderasiatischen Archäologie: Neue Forschung über Kaukasien und Anatolien in der Bronzezeit (Publications of the Georgian-Italian Shida Kartli Archaeological Project, 2) (Proceedings of the international Humboldt-Kolleg Venice, January 9th -January 12th, 2013)* (Subartu 38), Turnhout: Brepols, 2017, 153-171.

Sajjaddi SMS. 2006 Dahane-y Ghulaman: A City of Achaemenian in Sistan, Second Part. *The Journal of Archaeology and History* 2: 43–52 (in Persian).

Sajjaddi SMS. 2019 *Dahan-e Gholaman: An Achaemenid City in Southestern Iran*. Tehran: Ariyaramnah Publications.

Sajadi A. 2015 First season of archaeological excavation in Tepe Forudgah, Khorramabad-Lorestan. *Proceedings of the 14th Annual Symposium on the Iranian Archaeology*, Iranian Center for Archaeological Research, Tehran, pp. 82–87 (in Persian).

Sandiboev A, Murakami T, Begmatov A, Khojamov S H, Arziev K, Makhamadiev G, Alimov N, Azzamov G. 2023. 2022-yilda Qoʻrg‘ontepa yodgorligida olib borilgan arxeologik tadqiqotlar (Archaeological investigations conducted in 2022 at the Qo‘rg‘ontepa site), *Oʻzbekistonda Arxeologik Tadqiqotlar* 15, 244–248.

Sauer EW, Chologauri L, Gabunia A, Hopper K, Lawrence D, MacDonald E, Mashkour M, Mowat FA, Naskidashvili D, Pitskhelauri K, Priestman SMN, Shumilovskikh L, Simpson SJ, Tiliakou AI, Abe Y, Batt CM, Gagoshidze G, Gilmour B, Greenwood DP, Pickard C, Poole I, Radu V, Snape L, Snyder JR, Stoetzel E, Amiribeirami S, Astolfi M, Bailiff I, Beizaee Doost S, Bonato E, Clavel B, Debue K, Decruyenaere D, Diana A, Fathi H, Jajanidze R, Kamjan S, Kainaat T, Khazaeli R, Kranioti E, Lemoine M, Philip G, Shupe C, Shikaku R, Stern B, and Stetkiewicz S. 2020 *Dariali: The "Caspian Gates" in the Caucasus from Antiquity to the age of the Huns and the Middle ages: The joint Georgian-British Dariali gorge excavations & surveys of 2013-2016.* (Vol. 1). Oxbow Books.

Savchenko A. 1996 Urgut Revisited. *ARAM. The Journal of the Society for Syro- Mesopotamian Studies* 8(1-2), 333-354.

Savchenko A. 2005 По поводу кхристианского селения Ургут” (On the Christian settlement of Urgut), *Zapiski Vostochnogo otdeleniya Rossiĭskogo arkheologicheskogo obshchestva* 2. 27, St. Petersburg.

Savchenko A. 2010 Östliche Urkirche in Usbekistan. *Antike Welt* 2(10), 73-82.

Scerrato U. 1962 A probable Achaemenid zone in Persian Sistan. *East and West* 13(2/3): 186–197.

Scerrato U. 1966 Excavations at Dahan-I Ghulaman (Sistan-Iran), First Preliminary Report (1962-1963). *East and West* 16(1/2): 9–30.

Serrone E, Maini E, Curci A, Mantellini S, Berdimuradov A. 2021. Animal Exploitation in the Samarkand Oasis (Uzbekistan) at the Time of the Arab Conquest: Zooarchaeological Evidence from the Excavations at Kafir Kala. in *Archaeozoology of Southwest Asia and Adjacent Areas XII* (eds. J. Daujat et al.), Proceedings of the Thirteenth International Symposium, University of Cyprus, Nicosia, Cyprus, June 7-10, 2017, Lockwood Press 3, 221–30.

Shishkin VA. 1963 Varakhsha. Akademii Nauk SSSR, Moskva.

Shojaee-Esfahani A. 2018 The Fortified Landscape of Isfahan. in: D. Durand-Guédy, R. Mottahedeh, and J. Paul (eds.), *Cities of Medieval Iran*, Brill, *Eurasian Studies* 16(1-2): 177-216. <https://doi.org/10.1163/24685623-12340053>

Shojaee-Esfahani A, Aarab A, Arab EA, Kalantar S, Hadi Z, Hashemi F. 2017 Excavations at the Jahānnamā complex: urban archaeology at Isfahan, Iran. *Antiquity* 91(355): e7. doi:10.15184/aqy.2016.230.

Shojaee-Esfahani A, Rafiei-Alavi B. 2020 Short Report on the Survey at the Eastern Part of the Zāyandeh-Rud Basin, Kafarved (Kafrood)-Varzaneh Plain, Summer 2018. In: R. Shirazi and Sh. Hurshid (eds.), *Proceedings of 17th Annual Symposium on the Iranian Archaeology 2018 – 2019*, RICHT, Vol. 2: 722-731 (in Persian).

Sołtysiak A, and Naseri R. 2017 Human remains from Deh Dumen, Iran, 2013-2016. *Bioarchaeology of the Near East* 11: 70–75.

Soraghi S, Zeynivand M. 2022 The Susa salvage project in 2019, southwestern Iran. Historia i Świat 11: 71-79. DOI:10.34739/his.2022.11.04.

Stampfli. 1983. H.R. The fauna of Jarmo, with notes on animal bones from Matarrah the Amuq and Karim Shahir. in L. Braidwood, R. Braidwood, B. Howe, C. Reed, P.J. Watson (Eds.), Prehistoric Archaeology Along the Zagros Flanks. Studies in Ancient Oriental Civilization 105, University of Chicago Oriental Institute, Chicago (1983), pp. 431-483

Stark S, Rouse L М, Mirzaakhmedov S J, Silvia Z, Hunter S А, Bek T, Rakhmanov H, Bayani N. 2024 The Final Bronze/Early Iron Age in the Old Zerafshan Delta, Uzbekistan: Pilot Investigations at Kimirek-kum-1. *Journal of Field Archaeology* 49(4), 243–263. (doi.org/10.1080/00934690.2024.2330201)

Stöllner T, Aali A, & Bagherpour Kashani N. (Eds.). 2020 *Tod im Salz. Eine archäologische Ermittlung in Persien*. Begleitbuch, Katalog und Graphic Novel. Veröffentlichung aus dem Deutschen Bergbau-Museum 246 (pp. 203–208). Nünnerich–Asmus Verlag Media.

Stöllner T, Gambashidze I, Al-Oumaoui I, Baldus T, Berthon R, Belošić A, Bode M, Boenke N, Broomand N, Bungardt J, Courcier A, Fehren-Schmitz L, Ghalichi A, Gogochuri G, Haak W, Kvavadze E, Jansen M, Löffler I, Mindiashvili G, Murvanidze B, Otkhvani N, Schapals F, Senczek S, Tshornaliani N, Vautrin A. 2023 Between Valleys and Mountains. The Dzedzvebi Plateau as an Intermediate Settlement Site of Chalcolithic and Early Bronze Age Communities in the Lesser Caucasus. *Archaeologia Austriaca* 107, 65-136.

Stöllner T, Aali A, Boenke N, Davoudi H, Draganits E, Fathi H, Franke KA, Herd R, Kosczinski K, Mashkour M, Mostafapour I, Nezafati N, Öhrström L, Rühli F, Saeidi S, Schapals F, Schimerl N, Sikorski B, Zifar H. 2024 Salt Mining and Salt Miners at Talkherud–Douzlākh, Northwestern Iran: From Landscape to Resource-Scape. *Journal of World Prehistory* 37: 61-140. [https://doi.org/10.1007/s10963-024-09183-z](https://d.docs.live.net/9631f441bde6656f/Desktop/%20https:/doi.org/10.1007/s10963-024-09183-z)).

Suleymanov R Kh. 1984. Rezul’taty predvaritel’nogo izucheniyja gorodishcha Romitan (Results of the Preliminary Study of the Ancient Settlement of Romitan). *IMKU* 19, 118–129.

Taheri K, Mashkour M, Biglari F, & Morad Nezhad AA. 2007 Zilou Cave: A Karstic Shaft with Animal Bones at Kermanshah Region, Western Iran. In *Proceedings of the 11th Symposium of the Geological Society of Iran* (pp-3340). Ferdowsi University of Mashhad, Mashhad.

Tavassoli MM, Sajjadi A, Barani-Nia S, Tavassoli M. 2016 A Bronze Age Grave Discovered at Tepe Foroudgah, Iran: An Anthropological and Palaeopathological Study. *Mediterranean Journal of Social Sciences* 7(9): 259-269.

Teramura H, Uno T, Murakami T, Begmatov A, Berdimurodov A, Bogomolov G, Sandiboev A. Suemori K, Oshikane H. 2024 Excavations at Kafir-kala in Uzbekistan in 2023 – The Residential Area (Shahristan) of the Sogdian Royal Residence. *Ancient Orient Revealed through Excavations in 2023, Proceedings of the 31th Annual Meeting of Excavations in West Asia. Japanese Society for West Asian Archaeology*, 117-121 (In Japanese).

Tsuneki A. et al. 2024. Preliminary report of the Charmo (Jarmo) prehistoric investigations 2023,. 45, pp. 1-47.Directorate of Antiquity of Sulaymaniyah

Uerpmann H.-P, and Frey W. 1981 Die Umgebung Gar-e Kamarband (Belt-Cave) und Gar-e Ali Tappe (Beh-Sahr, Mazandaran, N-Iran) heut und im Spatpleistozan. In *Beitrige zur Umweltgeschichte des Vorderen Orients: Contributions to the Environmental History of Southwest Asia* *(Tubinger Atlas Des Vorderen Orients)* 8, edited by Frey Wolfgang and Hans-Peter Uerpmann, 134-190. Wiesbaden: Dr Ludwig Reichert Verlag.

Usami T, Begmatov A, Uno T, Berdimuradov A, Bogomolov G. 2017 Archaeological Excavation and Documentation of Kafir Kala Fortress. *Studies in Digital Heritage* 1, 785–96. (doi.org/10.14434/sdh.v1i2.23267)

Vahdati AA, and Zhang L. 2019 Brief report on the second season of Joint Iranian-Chinese excavations at Tepe Naderi in the Upper Atrak Valley, northeast Iran. In: *16th annual symposiumon the Iranian Archaeology*, 5-6 March 2019, R. Shirazi (ed.), Tehran: RICHT: 526-529 (in Persian).

Vahdati AA, Biscione R, La Farina R, Mashkour M, Tengberg M, Fathi H, & Mohaseb FA. 2019 Preliminary report on the first season of excavations at Tepe Chalow: New GKC (BMAC) finds in the plain of Jajarm, NE Iran. In *The Iranian Plateau during the Bronze Age: Development of Urbanisation, Production and Trade* 1, MOM Éditions: 179–200.

Vahdati AA, Zhang L, Shui T, Liu B, Jia X, Fathi H, Tengberg M, and Mashkour M. 2024 Iran-China Joint Archaeological Excavations at Tepe Naderi, Shirvan, Northern Khorasan: Preliminary Report of the 2016 and 2018 Seasons. In: *New Voices In Iranian Archaeology*, K. Alizadeh and M. Cifarelli (eds.), Oxford: Oxbow Books: 57-74.

Vautrin A. 2025 From summer pasture to mountain village: an archaeozoological approach to the establishment of agro-pastoral communities in the Caucasus mountains (VIth-IInd mill. BCE). Muséum National d’Histoire Naturelle, Paris.

Vautrin A., Bosch D., Balasse M., Bray F., Bruguier O., Fiorillo D., Le Meillour L., Bakhshaliyev V., Marro C., Mashkour. M and Berthon R. (Accepted and reviewed). Caprine Management during the Neolithic in the South Caucasus: Insights into Zootechnical Practices and Pastoral Mobility through Archaeological and Isotopic Studies (δ^18^O, δ^13^C, ^87^Sr/^86^Sr) at Kültepe I (Nakhchivan, Azerbaijan). *Paléorient*

Zhang L, Wei L, Ziqi G, Yanchao Q, Jingxin Q, Tao S, and Vahdati AA. 2021 Brief report on the archaeological survey at Tepe Naderi, Iran. *Kaoguxue Jikan* 24: 215-227 (in Chinese with English Abstract).

**Website:**

<https://whc.unesco.org/en/list/1397/> (UNESCO World Heritage Nomination Dossier — *Masjed-e Jāmé of Isfahan*).

<https://whc.unesco.org/en/list/1455> (UNESCO World Heritage Nomination Dossier — *Susa*).

<https://whc.unesco.org/en/tentativelists/5275/> (UNESCO World Heritage Nomination Dossier — *Touran Biosphere Reserve*).
