# Supplementary material for: Bony labyrinth taxonomic identification and long-term evolution of livestock in the Southwest and Central Asia
Source: R Soc Open Sci. 2026 Oct 7;13(10):261493. doi: 10.1098/rsos.261493 (PMC13641910; doi:10.1098/rsos.261493)
Supplement: Supplementary file 1 [file rsos.261493.zip › rsos.261493/rsos261493_si_021.docx]

##### **SI_Text 4.** Morphometric identification of archaeological specimens detailed. Supervised (pLDA) and unsupervised (clustering) analyses were used to assign archaeological specimens to taxonomic groups based on modern reference datasets.

Predictive Linear Discriminant Analysis (pLDA) based on the modern sheep and goat specimens, achieved 100% cross-validation accuracy using the first six PCs. Archaeological specimens were identified using the first six PCs, selected for maximal group discrimination; all 100 resampled LDAs yielded CVPs of 100%. This pLDA trained on modern reference species (sheep, goat, Asiatic mouflon and urial) and modern non-reference species identified two subgroups among the archaeological specimens: - 173 specimens identified as caprines; - 54 specimens classified as non-caprines.

Among the 173 archaeological caprine specimens, the trained pLDA model assigned 105 specimens to goats and 68 to sheep*,* with 93 pLDA iterations having CVP above 90%. Both unsupervised (clustering) and supervised (pLDA) approaches yielded highly consistent identifications, with only six specimens (HHJM8-a, MMDD10, MMDG04, MMKK02, MMZA01b and NTS1292) showing discrepancies between the two methods. Focusing on the non-caprine specimens (archaeological and modern, excluding hominids), a first clustering analysis based on seven PCs identified seven distinct groups: - 31 archaeological specimens with the modern cattle and one modern roe deer; - six archaeological specimens with one modern roe deer, one red deer and the Arabian sand gazelle; - three archaeological specimens with one modern red deer and the modern cat, donkey, fallow deer, fox, horse and saiga antelopes; - seven archaeological specimens with modern wild boars and pigs; - three archaeological specimens and the modern dogs; - four archaeological specimens and the modern goitered gazelles; - the modern rabbit and hare.

A second clustering analysis focusing on the previous specimens (excepting suids and canids), using the first nine PCs, identified four distinct groups: - 33 archaeological specimens with the modern cattle; - six archaeological specimens with the modern cat, donkey, fallow deer, fox, horse, red deer and roe deer; - five archaeological specimens with the modern Arabian sand gazelle and goitered gazelles; - the modern saiga antelopes. Then, a third clustering analysis excluding gazelles and saiga antelopes, based on the first eight PCs, identified five distinct groups: - 33 archaeological specimens with the modern cattle; - six archaeological specimens with the modern cat, donkey, fox, horse, red deer and roe deer; - the modern fallow deer.

Using this combination of supervised and unsupervised approaches, 228 of the 234 archaeological specimens were morphometrically identified as goats (n = 105), sheep (n = 68), cattle (n = 33), suids (n = 7), hominids (n = 7), gazelles (n = 5) and canids (n = 3). Six specimens remained unassigned and were classified as a “mixed” group. Subgroup assignments were refined through iterative clustering and high-confidence pLDA classifications (SI_Table 1).

A total of 16 archaeological specimens showed discrepancies between morphometric and anatomical identifications (SI Table 1), including:

- Six specimens morphometrically assigned to sheep but previously identified as goats;
- Two specimens reassigned from goat to sheep;
- Two specimens reassigned from cattle to goat;
- One specimen reassigned from sheep to hominids.
